# Results from a cluster-randomized trial to evaluate a microfinance and peer health leadership intervention to prevent HIV and intimate partner violence among social networks of Tanzanian men

**DOI:** 10.1371/journal.pone.0230371

**Published:** 2020-03-20

**Authors:** Suzanne Maman, Marta I. Mulawa, Peter Balvanz, H. Luz McNaughton Reyes, Mrema N. Kilonzo, Thespina J. Yamanis, Basant Singh, Lusajo J. Kajula

**Affiliations:** 1 Department of Health Behavior, Gillings School of Global Public Health, University of North Carolina, Chapel Hill, NC, United States of America; 2 Duke Global Health Institute, Duke University, Durham, NC, United States of America; 3 Department of Psychiatry and Mental Health, Muhimbili University of Health and Allied Sciences, Dar es Salaam, Tanzania; 4 School of International Service, American University, Washington, DC, United States of America; 5 Department of Psychiatry and Behavioral Sciences, Medical University of South Carolina, Charleston, SC, United States of America; Brown University, UNITED STATES

## Abstract

Despite calls to engage men in HIV and intimate partner violence (IPV) prevention efforts, effective approaches to reach and engage men in low-resource, high-HIV prevalence settings are limited. We identified and engaged social networks of mostly young men in a study designed to evaluate the efficacy of a combined microfinance and peer health leadership intervention to prevent HIV and IPV. We conducted a cluster-randomized trial among 60 social networks locally referred to as “camps” within Dar es Salaam, Tanzania. Camps were randomly assigned (1:1) to a microfinance and peer health leadership intervention or a control condition that received a brief delayed intervention after the study’s conclusion. Allocation was not masked to participants or researchers. Behavioral assessments were conducted at baseline and 30-months post-intervention launch, with biological samples drawn at 30-months to test for sexually-transmitted infections (STIs). Primary outcomes included prevalence of STIs and past-year IPV perpetration. Secondary outcomes included STI sexual risk behaviors and past-year HIV testing. Proximal intervention targets included inequitable gender norm attitudes and hope. A modified Poisson regression approach was used to estimate intention-to-treat intervention effects on outcomes assessed at the 30-month follow-up. We enrolled 1,258 men within 60 camps. Of these men, 1,029 (81.8%) completed the 30-month follow-up. There were no differences by condition in STI prevalence, IPV perpetration, or sexual risk behaviors at the 30-month follow-up. Intervention participants reported greater levels of past-year HIV testing, controlling for baseline testing (aRR 1.13 95% CI 1.005–1.28). They also reported significantly lower levels of inequitable gender norm attitudes (adjusted effect -0.11, 95% CI -0.21–0.003). We successfully engaged and retained social networks of men in this multilevel intervention study. While we did not see an effect on the primary outcomes, our intervention successfully improved HIV testing and reduced inequitable gender norm attitudes.

## Introduction

Finding effective strategies to reach young men and mobilize them to reduce their HIV risk is critical, given men’s control over the terms and conditions of most sexual partnerships. Inequitable gender norms and the resulting unequal distributions of power in relationships have a devastating impact on women, who continue to carry the greater burden of HIV in sub-Saharan Africa [1, 2]. These norms have negative consequences for men as well, leading to increased risk of physical and mental health problems, substance use, and low uptake of health-related services including HIV testing [3–6]. Norms related to gender power imbalance also contribute to the high prevalence of intimate partner violence (IPV) reported by women globally [7, 8]. While the importance of working with men to prevent HIV and violence has been increasingly recognized [9], we continue to lack effective approaches to reach and engage men in prevention efforts. Effective interventions that address the normative determinants of IPV perpetration and HIV risk behaviors are especially needed.

Social networks cultivate norms related to IPV and HIV-risk behaviors; therefore interventions targeting these social networks have an opportunity to transform these norms and change behaviors [10]. Social network interventions that work with network leaders to promote behavior change have demonstrated significant changes in norms and substantial reductions in network members’ risk behaviors [11]. Network leader interventions have been used successfully to increase condom use and reduce injection-related risk behaviors among injection drug users in Taiwan [12], increase physical activity among youth in the U.S [13], reduce sexual risk behavior and HIV stigma among men who have sex with men in Peru [14], and increase HIV testing and decrease condom use among MSM in Taiwan [15].

Guided by formative research [16], we developed a combined microfinance and peer health leadership intervention [17], designed for social networks of young men in Tanzania. The goal of the intervention was to reduce sexually transmitted infection (STI) prevalence and the perpetration of IPV by transforming the gender norms and addressing the structural determinants driving these issues in this context. Since a lack of income and employment opportunities can lead to feelings of hopelessness and serve as a source of distress and interpersonal conflict among men [18], the microfinance component of this intervention (i.e., provision of small loans and business skills training) was hypothesized to reduce the perpetration of IPV and HIV risk behaviors by increasing men’s hope for the future. The peer health leadership component was hypothesized to reduce the perpetration of IPV and HIV risk behaviors by transforming men’s attitudes towards gender roles and making them more equitable. We aimed to evaluate the efficacy of this combined intervention on prevalence of STIs, perpetration of IPV, HIV testing, sexual risk behaviors, as well as proximal intervention targets, including inequitable gender norm attitudes and hope among social networks of men locally referred to as ‘camps.” We identified camps in our formative research through PLACE (Priorities for Local AIDS Control Efforts), a venue-based sampling approach that is designed to identify high-transmission venues [19]. We applied this methodology to identify venues where young men at high risk for HIV and IPV perpetration socialize. Through interviews with community informants, and a process of verifying and mapping high transmission venues, we identified ‘camps” as the most common venues where networks of young men socialize. Camps are public spaces that networks of men claim and identify as their own. These are well organized networks of men that have a governance structure including a chairperson, secretary and treasurer. Men typically belong to one camp for an average of more than two years, and they report socializing with their peers in camps for several hours each day. Camps give the men, who are not in school and not regularly employed, an identity and a space to hang out each day. We have described camps in more detail in previous publications [20, 21].

## Methods

### Study design

We conducted a cluster-randomized trial among 60 camp-based social networks within four wards of Kinondoni Municipality in Dar es Salaam, Tanzania. We used a cluster design because we were interested in evaluating an intervention delivered within social networks [22]. Camp-based social networks serve as important socialization environments for men, propagating norms that are associated with men’s sexual partner concurrency [23], IPV perpetration [24, 25], as well as HIV testing [26, 27].

The study was designed to assess whether men in camps randomized to the combined microfinance and peer health leadership intervention had fewer STIs and reported less past-year perpetration of physical and/or sexual IPV compared to men in the control camps, who received a brief delayed intervention after the study’s conclusion [22]. Secondary outcomes included STI-related sexual risk behaviors and past-year HIV testing, and proximal intervention targets (i.e., hypothesized mediators) included inequitable gender norm attitudes and hope. Sixty camps were enrolled in the trial; 30 were randomly assigned to the two-year intervention condition (n = 621 men) and 30 were assigned to the control (n = 637 men). The study was approved by the University of North Carolina at Chapel Hill Institutional Review Board as well as the Muhimbili University of Health and Allied Sciences (MUHAS) Senate Research and Publications Committee.

### Participants

Prior to the baseline assessment, we used PLACE to identify all camps in the four wards where this study was being implemented [28]. In accordance with this methodology, we conducted community informant interviews to enumerate all camps within the study area. We then interviewed representatives from each camp in operation to assess camp eligibility and randomly selected 60 eligible camps for inclusion in the trial. To be eligible, camps had to have been in existence for at least 1 year and had to have between 20 and 79 members. Small camps (>20 members) and very large camps (≥ 80 members) were excluded to minimize variability in cluster size, ensure camps were likely to be meaningful sites for socialization, and maximize power to detect intervention effects. Camps were also excluded if a weapon had been used in a fight in the past 6 months or if a research assistant felt unsafe while verifying eligibility. We also excluded camps that had participated in pilot studies for this intervention. 294 operational camps were identified, 172 were eligible, and we randomly selected 60 of them for our trial. Consent for each camp to participate was obtained from a camp representative during this process. The procedures involved in identifying and assessing these camps, in addition to the multi-step, geographically-informed, probability-based sampling methods used to randomly select eligible camps while reducing the risk of contamination are detailed in the study protocol paper [22].

**Fig 1:**
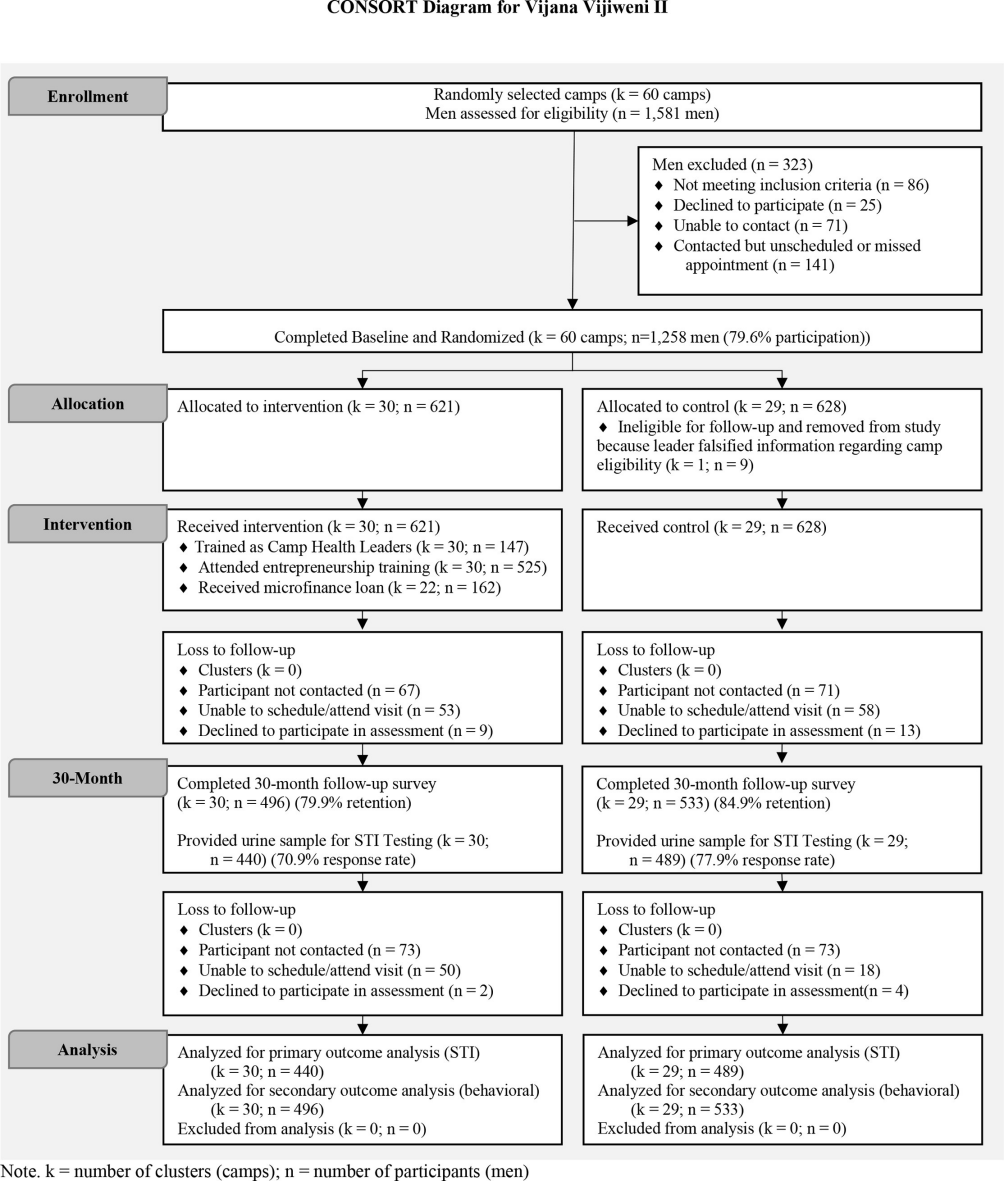
Trial profile.

After the 60 camps were selected, we conducted a census of the camps’ members by collecting camp rosters and contacting each camp member to confirm his or her eligibility. In order to be eligible, participants had to be at least 15 years old, have been a camp member for more than three months, visit the camp at least once a week, plan on residing in Dar es Salaam for the next 30 months, and be willing to provide contact information for a friend or family member for participant tracing purposes. Since many camps were comprised of both male and female members, we did not exclude women from participating in the study activities. Our *a priori* focus, however, was among the male participants. As indicated in Fig 1, of the 1,581 men identified in the census, we enrolled 1,258 men (79.6%) within 60 camps in the trial. Written informed consent was obtained from each participant.

### Randomisation and masking

To facilitate transparency within the community, the 60 camps were randomly assigned (1:1 ratio) to either the intervention or control condition, in which they received a brief delayed intervention after the study’s conclusion, during a meeting of our Community Advisory Board (CAB) using an allocation process implemented by one selected CAB representative. The random assignment to condition was conducted after the baseline assessment (described below). Details of the CAB and the process used to randomly assign camps to study condition can be found in the study protocol paper [22]. Due to the nature of the intervention, neither participants nor study staff could be blinded to condition assignment.

### Procedures

Both components of the intervention (microfinance and peer health leadership, described below) were implemented on an ongoing basis between March 2014 and March 2016.

#### Microfinance

All study participants who were members of camps assigned to the intervention condition were invited to attend a 5-day training on business development, entrepreneurship, and financing developed and led by a local microfinance institute (MFI). Individuals who completed the baseline survey, attended the training, deposited $5 into a savings account maintained by the MFI, paid loan fees, formed a solidarity group, and had their business plan approved by the group were eligible for an initial $100 loan at 18% (6 months) or 27% (9 month) interest. Repayment sessions were held weekly by a loan officer in a field location near the camps. Borrowers were expected to add $1.25 to savings in addition to paying their weekly due at each session. Individuals who repaid their loans in full were eligible for a second loan of $185, and then a third loan of $285. Booster business trainings were held every 6 months to share business challenges and successful strategies.

#### Peer health leadership

We recruited 20% of the membership in each intervention camp as peer health leaders. Leaders were nominated by their camp peers as individuals that exhibited the characteristics that they identified in a group meeting with our research staff as important attributes of leaders (e.g., someone who could be trusted with personal matters). The 20% of camp members who received the most nominations were invited to the training, which included education on leadership, gender-based violence and power, HIV and condom myths, safe sexual practices, communication and effective messaging. Peer health leaders were asked to use the skills in communication and influence to talk to their camp peers about reducing HIV risk and IPV perpetration as well as promoting HIV testing. Peer health leaders kept records of the number and types of conversations that they had with peers. One-day booster sessions were held every 6 months to review lessons and address challenges leaders faced in implementation.

#### Control condition

Camps randomized to the control condition received a brief, delayed intervention delivered after the final assessment. Men in the control camps were invited to attend the same training that the peer health leaders received which included information about HIV/AIDS, IPV and discussions about power and control within relationships. This training was facilitated by the same project team members and covered the same content and training strategies. Men in the control arm were also invited to attend a brief microfinance training implemented by our partner MFI organization which covered information about developing a business, marketing strategies and applying for a microfinance loan. Men who were interested in opening a microfinance loan with a group, were invited to meet with a loan officer from the MFI. The MFI opened a branch in one of the wards where the study was implemented, making it more accessible to all community members in the study area.

#### Assessments

Assessments were conducted at baseline, 12-months, and 30-months post-intervention launch, with biological samples drawn at baseline and 30-months to test for STIs. For the current study, we analyzed data collected from participants at two time points: (1) a baseline assessment administered between October 2013 and March 2014, and (2) a 30-month follow-up assessment administered between July 2016 and November 2016, approximately 30-months after the intervention launch. We examined intervention effects on outcomes assessed at the 30-month follow-up assessment because this was the only round of data collection that occurred once the intervention had been completed. Both the baseline and the 30-month follow-up assessments took place within study field offices located in the study area and were conducted using computer-assisted personal interviews (CAPI) on tablet devices.

### Outcomes

#### Sexually transmitted infections

To assess the prevalence of STIs at baseline and during the 30-month follow-up, we collected and tested urine samples using Multiplex PCR to detect both Chlamydia trachomatis (CT) and Neisseria gonorrhoea (NG). The Trichomonas vaginalis Real-TM test was used to detect Trichomonas vaginalis (TV). Study participants who were identified with NG, CT, or TV were considered to have an STI and were offered free treatment by a study clinician according to the standard of care in Tanzania.

#### IPV perpetration

The perpetration of past-year sexual and/or physical IPV was assessed using an adapted version of the World Health Organization violence against women instrument [8]. Participants were asked whether they had ever perpetrated 6 physically violent and 3 sexually violent acts against a current or former sexual partner. Participants who reported having perpetrated a specific act of violence were asked to report how many times they perpetrated that act in the last 12 months (never, once, 2–3 times, 4–10 times, and more than 10 times). The frequency of physical and sexual IPV perpetrated within the last 12 months was dichotomized as any vs. no sexual and/or physical IPV perpetrated within the last 12 months.

#### Sexual risk behaviors

We assessed inconsistent condom use, number of past-year sexual partners, as well as sexual partner concurrency among all sexually-active participants. *Inconsistent condom use* was assessed for men’s three most recent sexual partners by asking participants to report both the number of sexual acts over the most recent month of each relationship and the number of times that a condom was used during this time period for each partner. Using the percent of reported sex acts where condoms were used, condom use was dichotomized as “consistent condom use” (100% use) vs. “inconsistent condom use” (less than 100%). *Number of past-year sexual partners was assessed by asking* participants to state how many people they had sex within the last 12 months. Interviewers clarified that participants were to include people they had sex with only once and people they had sex with regularly (e.g., a spouse). If participants couldn’t recall the exact number, they were asked to provide a best guess. *Sexual partner concurrency* was evaluated by self-report of any overlapping sexual partnerships for men’s past three sexual partners. When enumerating current and past sexual relationships, participants were asked to report if they had sex with anyone else during any of these partnerships. Sexual partner concurrency was dichotomized as no overlapping sexual relationships vs. any instance of concurrent sexual relationships within any of the three most recent relationships within the past 12 months.

#### HIV testing

Past-year HIV testing was assessed by asking participants whether they have had a test for HIV in the last 12 months. At the baseline assessment, participants were also asked whether they had ever had a test for HIV.

#### Inequitable gender norm attitudes

To measure peer network gender norms, we used a 15-item adapted version of the Gender Equitable Men (GEM) scale [18]. Men were asked how strongly they agreed or disagreed with statements such as “it is the man who decides what type of sex to have.” Responses ranged from 1 = strongly disagree to 4 = strongly agree. We averaged the responses across the 15 items to create a composite scale for each individual (Cronbach’s α = .91).

#### Hope

To measure hope, we used Snyder’s State Hope Scale [29]. Men were asked how strongly they agreed with six statements, such as “there are lots of ways around any problem that I am facing right now.” Response ranged from 1 = strongly disagree to 4 = strongly agree. Responses were averaged to create a composite scale for each individual (Cronbach’s α = .69).

#### Demographics

We used responses from the baseline assessment to assess the following demographic characteristics: age (in years, categorized into four categories: 15–19, 20–24, 25–29, and 30 or more years), socioeconomic status (terciles of a wealth index assessing ownership of 10 different household assets), highest level of education obtained (categorized as primary school or less, some secondary school, or secondary school completed or greater), marital history (whether ever married), whether the participant had a sexual partner at baseline, and duration of camp membership (in years).

### Statistical analysis

First, we used descriptive statistics to summarize sociodemographic variables and baseline levels of each outcome variable. To establish baseline equivalency, we assessed differences between groups with the *t-test* for continuous variables and the *Χ*^2^ test for categorical outcomes. Second, we conducted attrition analysis using logistic regression adjusting for clustering within camps to examine whether treatment status predicted drop-out and to test for differential lost to follow-up across conditions. Third, to assess the effects of the intervention on primary and secondary outcomes, we used an intention-to-treat approach (i.e., all individual participants in camps that were randomized to intervention or control were included in analyses regardless of level of intervention exposure), with statistical models also accounting for clustering of participants within camps.

To examine intervention effects on our primary and secondary outcomes, we used a modified Poisson regression approach for estimating relative risks (RRs) from clustered prospective data using generalized estimating equations to account for clustering at the camp-level [30]. We also computed adjusted risk ratios (aRR), controlling for pre-specified covariates believed to have an important influence on the outcomes, including demographics (age, socioeconomic status, education, martial history, partner status), as well as duration of camp membership and baseline level of the outcome measures (when applicable). We used robust standard errors and an exchangeable working correlation matrix. To examine intervention effects on proximal intervention targets (i.e., levels of inequitable gender norm attitudes and hope), we used mixed linear models to compute estimates of the intervention’s effects while accounting for the clustering of participants within camps. Sensitivity analyses were conducted by restricting the analytic sample to those in a sexual relationship. Finally, we conducted supplementary moderation analysis to examine whether treatment group effects were moderated by ward, age group, SES, and/or levels of camp cohesion. To do this we used the same analytic approach used to assess intervention effects on primary and secondary outcomes and included the appropriate interaction terms. We conducted our analyses using SAS software Version 9.4. The study was powered to detect protective effects of the intervention of .57 for STI prevalence and .65 for perpetration of sexual or physical IPV perpetration with power equal to .80 and a 2-sided α equal to 0.05. This calculation was based on anticipated average camp sizes of 26.4 men, anticipated retention of 80%, and camp intraclass correlation (ICC) estimates between 0.00 and 0.01 STIs and 0.032 for behavioral measures. There was no data monitoring committee involved with this study. The trial is registered with ClinicalTrials.gov, number NCT01865383.

## Results

Between October 8, 2013 and March 23, 2014, we enrolled 1,258 men within 60 camps in the trial (Fig 1). Of the 60 camps, 1 camp (n = 9 men) was removed from the trial after random assignment because the leader falsified information regarding the camp’s eligibility for the study. Thus, 30 camps (n = 621 men) were allocated to the intervention condition and 29 camps (n = 628 men) to the control condition.

The baseline characteristics of the individual and camps enrolled in the trial were generally similar across study conditions (Table 1). Significant differences by condition were observed for the number of past-year sexual partners and the proportion of participants who reported having a current sexual partner. The mean age of enrolled participants was 26.0 years and the majority (56.8%) had completed only primary school education or less. Most participants (77.7%) had never been married though the vast majority (89.1%) had been sexually-active prior to the baseline assessment and more than half reported having a current sexual partner (57.7%).

**Table 1:** Sample characteristics at baseline assessment among male study participants (n = 1,249 men; k = 59 camps).

	**Intervention, (n = 621), No. (%) or Mean ±SD**	**Control, (n = 628), No. (%) or Mean ±SD**	***p*^a^**
Age in years			
15–19	114 (18.4)	119 (19.0)	.91
20–24	192 (30.9)	182 (29.0)	
25–29	162 (26.1)	168 (26.8)	
30+	153 (24.6)	159 (25.3)	
Education			
Primary school or less	356 (57.5)	351 (56.1)	.36
Some secondary school	65 (10.5)	82 (13.1)	
Secondary school completed or more	198 (32.0)	193 (30.8)	
SES			
Low	207 (33.3)	186 (29.7)	.37
Medium	217 (34.9)	229 (36.5)	
High	197 (31.7)	212 (33.8)	
Marital history			
Never married	478 (77.4)	490 (78.0)	.77
Ever married	140 (22.7)	138 (22.0)	
Ever had sex	552 (88.9)	561 (89.3)	.80
Number of sexual partners in last year			
0	70 (12.7)	78 (13.9)	.002
1	352 (63.8)	397 (70.8)	
2	77 (14.0)	40 (7.1)	
3+	53 (9.6)	46 (8.2)	
Current sexual partner	378 (60.9)	342 (54.5)	.02
Duration of camp membership (years)	6.0 **±**4.4	6.1 **±**4.3	.71
	**Intervention, (k = 30), Mean ±SD**	**Control, (k = 29), Mean ±SD**	***p***^a^
Camp size (# of members in camp)	26.0 **±**9.6	26.9 **±**11.5	.76
Number of participants enrolled in trial	20.7 **±**8.7	21.7 **±**9.3	.69
30-month response rate^b^	80.9 **±**13.1	83.4 **±**15.1	.50
Duration of existence (years)	3.9 **±**1.0	4.0 **±**0.9	.60

^a^ Between-condition differences for categorical variables and continuous variables were examined using *χ*2 tests and *t* tests, respectively.

^b^ Response rates calculated for each camp as the percent of participants enrolled in the trial who completed the 30-month assessment.

k = number of clusters (camps); n = number of participants (men)

Of 1,249 men allocated to the study conditions, 1,029 (82.4%) completed the 30-month follow-up assessment. At the follow-up, retention was higher in the control condition (84.9%) compared to the intervention (79.9%; p = .02). In analyses examining predictors of drop-out, having ever perpetrated IPV at baseline was associated with a *lower* odds of being lost-to-follow-up (AOR 0.66; 95% CI: 0.45–0.98). No other sociodemographic or outcome variables predicted loss to follow-up and predictors of drop-out did not differ for treatment and control groups, suggesting that attrition was not selective.

STI prevalence at 30-months was similar for both conditions (28.3% and 27.1% in intervention and control, respectively) (Table 2). There was no evidence that the intervention reduced STI prevalence (aRR 1.06, 95% CI 0.86–1.31). Overall proportions of past-year IPV perpetration increased over time for both study conditions, and no differences in past-year IPV perpetration or onset of IPV perpetration among never perpetrators at baseline by study condition were found at the 30-month follow-up (aRR 1.14, 95% CI 0.91–1.44 and aRR 1.13, 95% CI 0.85–1.51, respectively). Similar increases in sexual risk behaviors over time for both study conditions were observed for proportions of participants reporting inconsistent condom use, number of sexual partners, and sexual partner concurrency. No significant intervention effects were observed for these sexual risk behaviors at the follow-up assessment.

**Table 2:** Unadjusted and adjusted intervention effects on primary and secondary outcomes at 30-month follow-up.

		Intervention		Control		Unadjusted			Adjusted^c^		
		No. of Partic.	Mean or Prop.	No. of Partic.	Mean or Prop.	RR^a^ or IRR^b^	95% CI	P value	RR^a^ or IRR^b^	95% CI	P value
**STI**											
	30-month follow-up	441	28.3	487	27.1	1.06	(0.84, 1.33)	0.62	1.06	(0.86, 1.31)	0.57
**IPV Perpetration (Physical/Sexual)—Past Year**											
Any IPV perpetration											
	Baseline	621	16.4	628	15.9						
	30-month follow-up	496	23.2	533	19.9	1.17	(0.92, 1.47)	0.20	1.14	(0.91, 1.44)	0.26
Onset of IPV perpetration (among never perpetrators at baseline)											
	30-month follow-up	391	19.2	437	17.2	1.12	(0.84, 1.49)	0.45	1.13	(0.85, 1.51)	0.41
**Other HIV-Related Behaviors**											
Any unprotected sex acts (among sexually active)											
	Baseline	552	58.2	561	54.0						
	30-month follow-up	496	64.1	530	68.7	0.93	(0.86, 1.02)	0.12	0.96	(0.89, 1.05)	0.41
Number of sexual partners (among sexually active)											
	Baseline	552	1.4	561	1.3						
	30-month follow-up	496	2.2	528	2.0	1.11	(0.92, 1.34)	0.28	1.09	(0.89, 1.34)	0.41
Sexual Partner Concurrency (among sexually active)											
	Baseline	552	19.0	561	15.7						
	30-month follow-up	496	39.7	530	34.5	1.15	(0.98, 1.35)	0.09	1.14	(0.96, 1.34)	0.13
HIV Testing in last 12 months											
	Baseline	621	45.3	627	46.3						
	30-month follow-up	496	52.6	533	47.3	1.11	(0.98, 1.26)	0.09	1.13	(1, 1.28)	0.04

*p < .05

** *p* < .01

*** *p* < .001

^a^ Risk Ratios obtained using modified poisson regression (proc genmod).

^b^ IRR obtained using negative binomial regression (proc genmod) for number of sexual partners.

^c^ Adjusted models control for age, SES, education, marital history, whether partnered at baseline, number of sexual partners at baseline, duration of camp membership, and baseline level of the outcome. Note that STI prevalence and onset of IPV perpetration did not have baseline levels of outcome.

Men in the intervention condition reported greater levels of HIV testing compared to men in the control (aRR 1.13, 95% CI 1.005–1.28, p = .04) at the 30-month assessment, controlling for baseline HIV testing history.

While there was no difference in hope across condition (Table 3), men in the intervention arm reported significantly lower levels of inequitable gender norm attitudes at the 30-month follow-up (adjusted effect -0.11, 95% CI -0.21–0.003, p = .04).

**Table 3:** Unadjusted and adjusted intervention effects on mediators at 30-month follow-up.

		Intervention		Control		Unadjusted Intervention Effects			Adjusted Intervention Effects^b^		
		No. of Partic.	Mean	No. of Partic.	Mean	Effect^a^	95% CI	P value	Effect ^a^	95% CI	P value
*Inequitable gender norm attitudes* (Range 1–4; higher more inequitable)											
	Baseline	621	2.0	627	2.0						
	30-month follow-up	496	2.0	533	2.1	-0.1	(-0.2, 0.0)	0.06	-0.11	(-0.2, 0.0)	0.04*
Hope (Range 1–4)											
	Baseline	621	3.1	628	3.1						
	30-month follow-up	496	3.2	533	3.2	0.0	(-0.1, 0.1)	0.99	-0.0001	(-0.1 0.1)	1.00

*p < .05

** *p* < .01

*** *p* < .001

^a^ Intervention effects obtained using proc mixed.

^b^ Adjusted models control for age, SES, education, marital history, partnered at baseline, duration of camp membership, and baseline level of the mediator.

These results were consistent with the results of the sensitivity analyses conducted among men in a sexual relationship. Supplemental moderation analyses conducted to examine whether treatment effects differed as a function of ward, age group, SES, and/or levels of camp cohesion produced no evidence of differential treatment effects for any of the variables examined.

## Discussion

We evaluated a combined microfinance and peer health intervention among social networks of men in Tanzania. The intervention did not have a significant effect on the primary outcomes or secondary STI-related risk behavior outcomes. However, the intervention did lead to a significant increase in HIV testing as well as a reduction in inequitable gender norm attitudes.

We successfully engaged and retained social networks of men in this multi-component intervention study. Finding effective approaches to engage young men in HIV and IPV prevention interventions has remained a major challenge in the field. Our approach leveraged the fact that we were able to identify existing social networks of men and access them through camps. The prevalence of past-year IPV perpetration in our sample at baseline (16.4% intervention and 15.9% control) is difficult to compare with other global studies of IPV perpetration among men due to differences in sampling and measurement. Among adult, rural men in a cluster-randomized trial in Ghana, 23% reported past year perpetration of IPV [31]. In South Africa, nearly a third of rural, sexually experienced men 15–26 years (31.8%) reported lifetime violence against women [32]. In an international study of men’s perpetration of violence, 39% of adult men in Rwanda and 45% of adult men in the Democratic Republic of the Congo reported ever perpetrating IPV [33].

The fact that men in camps engaged in the intervention and we were able to follow up with them over time suggests that camp-based networks are viable targets for interventions. The data we collected on these social networks provided us with important insight on how young men’s social networks influence their HIV risk behaviors [25–27] and IPV [17].

The combined microfinance and peer health leadership intervention successfully improved HIV testing and reduced inequitable gender norm attitudes. The impact of the intervention on testing may be due to the fact that HIV testing is an individual behavior that we know is influenced by norms and behaviors of close friends [25–27], therefore this is an outcome that may be effectively addressed through the peer health leader component. Increasing HIV testing uptake, particularly among young men at risk for HIV, is an important emphasis of UNAIDS’ 90-90-90 strategy to control the HIV epidemic [34]. Men experience significantly higher rates of AIDS-related mortality compared to women in sub-Saharan Africa, in part due to their lower rates of HIV testing [35]. Our findings suggest that a social network approach to promote HIV testing can be effective at increasing HIV testing uptake among young men.

Our intervention resulted in significantly more equitable gender norms among men in the camps that were randomized to the intervention arm. We conceptualize inequitable gender norm attitudes as being on the pathway between the intervention and the HIV and IPV outcomes. It is possible that with more intervention exposure and follow-up time we would have seen subsequent changes in IPV and other HIV risk behaviors. The idea that extending our follow-up period may have enabled us to see significant effects is consistent with findings from other IPV prevention interventions. For example, Coker and colleagues found significant effects on reduction in IPV using a bystander intervention, however several of these effects were not identified until more than 3 years after the intervention launched [36]. There are few examples of rigorously evaluated IPV prevention interventions targeting men. One rigorously evaluated interventions shown to reduce men’s reported perpetration of violence is Stepping Stones [37]. This intervention which includes 50 hours of participatory learning workshops was evaluated in 70 rural villages in South Africa. While the intervention did not lead to a reduction in HIV-incidence (primary outcome), there was a significant intervention effect for men in their reported perpetration of violence and reduction in risk behaviors.

The combined intervention did not lead to significant changes in our primary outcomes, HIV risk and IPV perpetration. It is possible that the intervention we designed and implemented was not powerful enough to lead to behavioral changes. It is also possible that the intervention did not address important determinants of men’s IPV and HIV risk behaviors. For example, a significant proportion of men in our sample reported violence victimization and exposure to violence as a child and adolescent and men who reported sexual violence victimization before the age of 12 years had over three times the odds of perpetrating IPV in the last 12 months compared to men who did not report childhood sexual violence victimization [38]. It may be critical to address men’s own trauma before or at the same time as trying to change their use of violence against partners. It is also possible that the intervention was not the best fit for this population. Our motivation for incorporating a microfinance component to the intervention was driven by the fact that in the formative phase men said they wanted greater access to business training and capital for their businesses, and many camps were already engaging in business enterprise together. We assumed that since we were working with existing social networks, this would be a strength for the microfinance model. However, there may have been existing dynamics within these groups that made loan uptake more challenging. We were working with a population of young men who had little to no previous access to credit. While we provided the men with business training, it is possible that they need more preparation and training to be able to uptake and succeed with these type of loans. The interest rates for this MFI is consistent with interest rates offered by MFI in other settings. While it is possible that the high interest rates deterred some men from participating in the microfinance, when we explored the barriers to uptake of loans, the most common reasons cited included perceptions that they were not prepared to take a loan, lack of trust of their peers as loan group members, and a perception that the loan amounts were too small [39]. With regard to the peer leader component, it is possible that the criteria we used to select the leaders for the training did not match the qualities of what makes an effective change agent within these social networks. For example, while our approach was designed to identify popular individuals who were named most frequently as having leadership traits, subsequent analyses using our 12-month interim data found that health leaders with increasing levels of popularity reported having fewer conversations about HIV-related topics with their peers [40]. These analyses also found that health leaders positioned between sub-groups in their networks reported having more conversations about gender-based violence with their peers, suggesting that strategically recruiting and supporting these individuals in future research trials could maximize intervention effectiveness. Leaders also reported challenges engaging their peers in conversations about gender-based violence, power and control in their relationships. They found it easier to engage peers in conversations about HIV prevention strategies including condoms and HIV testing. During booster training sessions we addressed these challenges through role playing and additional skill building. We also provided the leaders tools that they could use to start these conversations. For example, with the help of the leaders, we developed slogans that we printed onto t-shirts worn by the leaders designed to spark conversations with their peers. Slogans such as “Naacha Ukatili wa Kijinsia,Wewe?” in Swahili, which translates to “Stopping IPV, You?” did help the leaders start conversations but they reported these conversations were still more difficult to sustain with their peers [41].

The study was not without limitations. Due to funding and resource constraints we were not able to conduct a four-armed trial to test the unique and combined effects of the two intervention components and identify specific mediation pathways. We also recruited a sample of men who were regular members of camp-based social networks. These networks are well organized, and often high functioning, for example engaging in business enterprise together. Men who were regular members of these camps may not be representative of all men in these wards. Behavioral outcomes were primarily assessed through self-reported measures. It is possible that men did not accurately report sensitive behaviors related to HIV and IPV. It is notable, however, that we achieved over 80% retention at 30-months follow-up which is remarkable given this is an urban, highly mobile population.

### Conclusion

Our team identified social networks of young men in Dar es Salaam, Tanzania and enrolled them in an intervention designed to reduce HIV and IPV risk through a combination of microfinance and peer health leadership. While we did not see an effect on the primary outcomes, our combined microfinance and peer health leadership intervention successfully improved HIV testing and reduced inequitable gender norm attitudes. To our knowledge, this was the first intervention trial targeting social networks of young men in Africa to demonstrate an effect on norms and behaviors. The results have implications for how to identify and engage young men in urban settings like Dar es Salaam in prevention interventions.

## Supporting information

S1 Checklist CONSORT 2010 checklist of information to include when reporting a randomised trial*. (DOC)

S1 Data (PDF)

S2 Data (PDF)

S3 Data (CSV)

S1 Protocol (DOCX)
