# Supplementary material for: Results from a cluster-randomized trial to evaluate a microfinance and peer health leadership intervention to prevent HIV and intimate partner violence among social networks of Tanzanian men
Source: PLoS One. 2020 Mar 20;15(3):e0230371. doi: 10.1371/journal.pone.0230371 (PMC7083321; doi:10.1371/journal.pone.0230371)
Supplement: S1 Data — (PDF) [file pone.0230371.s002.pdf]

THE UNIVERSITY of NORTH CAROLINA at CHAPEL HILL

OFFICE OF HUMAN RESEARCH ETHICS
105 Mason Farm Road
Medical Building #52
CB #7097
University of North Carolina at Chapel Hill
Chapel Hill, North Carolina 27599-7097
(919) 966-3113
Web site: ohre.unc.edu
Federalwide Assurance (FWA) #4801

To: Suzanne Maman
Health Behavior and Health Education

From: Non-Biomedical IRB

Approval Date: 1/28/2013
Expiration Date of Approval: 6/11/2013

RE: Notice of IRB Approval by Full Board Review
Submission Type: Initial
Study #: 12-1111

Study Title: Microfinance and Health Intervention Trial for Young Men in Dar es Salaam, Tanzania
Sponsors: National Institutes of Health (NIH)

This submission has been approved by the IRB for the period indicated.

Study Description:
Purpose: The purpose of this intervention trial is to assess the efficacy of an intervention that combines microfinance with health promotion on reducing the incidence of sexually transmitted infections among young men, age 15 and older who socialize in what are called "camps" in Dar es Salaam, Tanzania.

Participants: Trained study staff will enroll 2808 men 15 years and older who socialize in what are called "camps" across 4 wards (equivalent to US census tract) of Dar es Salaam, Tanzania. Camps are enduring social groups of mostly men that have elected leadership, paid membership fees, and physical space to meet.

Procedures (methods): This is a cluster randomized controlled trial to assess the efficacy of an intervention designed to reduce risk for sexually tranmitted infections and gender-based violence among young men who socialize in camps in Dar es Salaam, Tanzania. To accomplish our <u>first primary aim</u> to enumerate and characterize camps within four wards in Dar es Salaam, study staff in Tanzania will conduct a PLACE assessment in each ward to generate a pool of camps for inclusion in the trial. To achieve our <u>second primary aim,</u> to evaluate the efficacy of a combined microcredit and health leadership intervention we will train study staff in Tanzania tol conduct a trial among 2,808 men in 54 camps that are randomly selected proportionally across four wards. Male camp members 15 and older will be screened and evaluated prior to enrollment. They will be interviewed 12 months and 30 months after enrollment. The intervention will be implemented for two years, and the total duration of the trial will be 5 years.

Regulatory and other findings:
This research meets criteria for waiver of parental or guardian informed consent according to 45 CFR 46.116(d) and 45 CFR 46.408(c). Minors aged 15-17 are considered to be independent adults in the research setting of men's "camps" in Tanzania.

Investigator's Responsibilities:
Federal regulations require that all research be reviewed at least annually. It is the Principal Investigator's responsibility to submit for renewal and obtain approval before the expiration date. You may not continue any research activity beyond the expiration date without IRB approval. Failure to receive approval for continuation before the expiration date will result in automatic termination of the approval for this study on the expiration date.

Your approved consent forms and other documents are available online at http://apps.research.unc.edu/irb/irb_event.cfm?actn=info&irbid=12-1111.

You are required to obtain IRB approval for any changes to any aspect of this study before they can be implemented. Any unanticipated problem involving risks to subjects or others (including adverse events reportable under UNC-Chapel Hill policy) should be reported to the IRB using the web portal at http://irbis.unc.edu.

Researchers are reminded that additional approvals may be needed from relevant "gatekeepers" to access subjects (e.g., principals, facility directors, healthcare system).

This study was reviewed in accordance with federal regulations governing human subjects research, including those found at 45 CFR 46 (Common Rule), 45 CFR 164 (HIPAA), 21 CFR 50 & 56 (FDA), and 40 CFR 26 (EPA), where applicable.
