# Supplementary material for: Results from a cluster-randomized trial to evaluate a microfinance and peer health leadership intervention to prevent HIV and intimate partner violence among social networks of Tanzanian men
Source: PLoS One. 2020 Mar 20;15(3):e0230371. doi: 10.1371/journal.pone.0230371 (PMC7083321; doi:10.1371/journal.pone.0230371)
Supplement: S2 Data — (PDF) [file pone.0230371.s003.pdf]

Application Cover Memo

Cover memo prepared by Suzanne Maman on 05/18/2012 at 07:21 AM

Dear Committee Chair/Members,

We are submitting a new IRB application for an intervention trial that will be conducted with young men in Dar es Salaam, Tanzania. This intervention trial is developed based on formative and pilot work that we have already conducted in Dar es Salaam. We received IRB approval for the formative/pilot work. We are preparing the IRB application to submit for review to the IRB at Muhimbili University of Health and Allied Sciences (MUHAS). We will submit to the UNC IRB the IRB approval from MUHAS as soon as we receive that from them. We have developed the consent form in English, which is included in this application. Once the UNC IRB has approved the English consent form, we will then have it translated. We will submit the verification of translation once the consent frm has been translated.

Thank you for your review,

Suzanne Maman

General Information

1. General Information

1. Project Title

Microfinance and Health Intervention Trial for Young Men in Dar es Salaam, Tanzania

2. **Brief Summary**. Provide a **brief non-technical description** of the study, which will be used in IRB documentation as a description of the study. Typical summaries are 50-100 words. Please reply to each item below, retaining the subheading labels already in place, so that reviewers can readily identify the content. PLEASE NOTE: THIS SECTION MAY BE EDITED BY THE IRB FOR CLARITY OR LENGTH.

Purpose: The purpose of this intervention trial is to assess the efficacy of an intervention that combines microfinance with health promotion on reducing the incidence of sexually transmitted infections among young men, age 15 and older who socialize in what are called "camps" in Dar es Salaam, Tanzania.

Participants: Trained study staff will enroll 2808 men 15 years and older who socialize in what are called "camps" across 4 wards (equivalent to US census tract) of Dar es Salaam, Tanzania. Camps are enduring social groups of mostly men that have elected leadership, paid membership fees, and physical space to meet.

Procedures (methods): This is a cluster randomized controlled trial to assess the efficacy of an intervention designed to reduce risk for sexually tranmitted infections and gender-based violence among young men who socialize in camps in Dar es Salaam, Tanzania. To accomplish our first primary aim to enumerate and characterize camps within four wards in Dar es Salaam, study staff in Tanzania will conduct a PLACE assessment in each ward to generate a pool of camps for inclusion in the trial. To achieve our second primary aim, to evaluate the efficacy of a combined microcredit and health leadership intervention we will train study staff in Tanzania tol conduct a trial among 2,808 men in 54 camps that are randomly selected proportionally across four wards. Male camp members 15 and older will be screened and evaluated prior to enrollment. They will be interviewed 12 months and 30 months after enrollment. The intervention will be implemented for two years, and the total duration of the trial will be 5 years.

3. Is this new study similar or related to an application already approved by a UNC-Chapel Hill IRB? Knowing this will help the IRB in reviewing your new study.

Yes

If yes, provide IRB study number here (and explain in the COVER MEMO why this is relevant to the current study and why it would be useful for the IRB to know).

08-0502

2. Project Personnel

1. Will this project be led by a STUDENT (undergraduate, graduate) or TRAINEE (resident, fellow, postdoc), working in fulfillment of requirements for a University course, program or fellowship?

   No

2. List all project personnel beginning with principal investigator, followed by faculty advisor, co-investigators, study coordinators, and anyone else who has contact with subjects or identifiable data from subjects.

   - List ONLY those personnel for whom this IRB will be responsible; do NOT include collaborators who will remain under the oversight of another IRB **for this study**.
   - If this is Community Based Participatory Research (CBPR) or you are otherwise working with community partners (who are not functioning as researchers), you may not be required to list them here as project personnel; consult with your IRB.
   - If your extended research team includes multiple individuals with limited roles, you may not be required to list them here as project personnel; consult with your IRB.

   The table below will access campus directory information; if you do not find your name, your directory listing may need to be updated.
   If a change to the Principal Investigator is requested during the course of the study, a PI Change Form must be submitted.

Liaison	Last Name	First Name	Department Name	Role	Detail
University of North Carolina at Chapel Hill (UNC-CH)					
	Maman	Suzanne	Health Behavior Operations	Principal Investigator	view
	Bowling	James	Health Behavior Operations	Co-investigator	view
	Hobbs	Marcia	Department of Medicine	Co-investigator	view
	Leatherman	Sheila	Health Policy and Management Operations	Co-investigator	view

NOTE: The IRB database will link automatically to UNC Human Research Ethics Training database and the UNC Conflict of Interest (COI) database. Once the study is certified by the PI, all personnel listed (for whom we have email addresses) will receive separate instructions about COI disclosures. The IRB will communicate with the personnel listed above or the PI if further documentation is required.

3. If this research is based in a center, institute, or department (Administering Department) other than the one listed above for the PI, select here. Be aware that if you do not enter anything here, the PI's home department will be AUTOMATICALLY inserted when you save this page.

Department	Health Behavior Operations

3. Funding Sources

1. Is this project funded (or proposed to be funded) by a contract or grant from an organization EXTERNAL to UNC-Chapel Hill?

   Yes

Is UNC-CH the **direct** recipient of any Federal funding for this study? You should answer 'yes' *only* if you are the grantee. You should answer 'no' if you are the recipient of a sub-award or contractor under the grant.

No Answer Provided

Funding Source(s) and/or Sponsor(s)

Sponsor Name	UNC Ramses Number	Sponsor Type	Prime Sponsor Name	Prime Sponsor Type	Sponsor/Grant Number	Detail
National Institutes of Health (NIH)	12-2336	Federal				view

2. Is this study funded by UNC-CH (e.g., department funds, internal pilot grants, trust accounts)?

No

3. Is this research classified (e.g. requires governmental security clearance)?

No

4. Is there a master protocol, grant application, or other proposal supporting this submission (check all that apply)?

- ✔ Grant Application
- ✘ Industry/Federal Sponsor Master Protocol
- ✘ Student Dissertation or Thesis Proposal
- ✘ Investigator Initiated Master Protocol
- ✘ Other Study Protocol

4. Screening Questions

The following questions will help you determine if your project will require IRB review and approval.

The first question is whether this is RESEARCH (click for details)

1. Does your project involve a systematic investigation, including research development, testing and evaluation, which is designed to develop or contribute to generalizable knowledge? PLEASE NOTE: You should only answer yes if your activity meets all the above.

Yes

The next questions will determine if there are HUMAN SUBJECTS (click for details)

2. Will you be obtaining information or biospecimens through intervention or interaction with the individual, and use, study, or analysis of the information or biospecimens? This would include any communication or interpersonal contact between investigator and subject such as using in-person or online questionnaires/surveys, interviews, focus groups, observations, treatment interventions, etc. PLEASE NOTE: Merely obtaining information FROM an individual does not mean you should answer 'Yes,' unless the information is also ABOUT them.

Yes

3. Will you be obtaining, using, studying, analyzing, or generating identifiable private information or identifiable biospecimens collected through means other than direct interaction? This would include data, records or biological specimens that are currently existing or will be collected in the future for purposes other than this proposed research (e.g., medical records, ongoing collection of specimens for a tissue repository).
OR
Will you be using human specimens that are not individually identifiable for FDA-regulated in vitro diagnostic (IVD) device investigations?

No

The following questions will help build the remainder of your application.

4. Will subjects be studied in the Clinical and Translational Research Center (CTRC, previously known as the GCRC) or is the CTRC involved in any other way with the study? (If yes, this application will be reviewed by the CTRC and additional data will be collected.)

No

5. Does this study directly recruit participants through the UNC Health Care clinical settings for cancer patients **or** does this study have a focus on cancer or a focus on a risk factor for cancer (e.g. increased physical activity to reduce colon cancer incidence) **or** does this study receive funding from a cancer agency, foundation, or other cancer related group? (If yes, this application may require additional review by the Oncology Protocol Review Committee.)

No

6. Are any personnel, organizations, entities, facilities or locations in addition to UNC-Chapel Hill involved in this research (e.g., is this a multi-site study or does it otherwise involve locations outside UNC-CH, including foreign locations)? You should also click "Yes" if you are requesting reliance on an external IRB, or that UNC's IRB cover another site or individual. See guidance.

Yes

5. Multi-site Study Information

1. Are UNC-affiliated researchers involved in research conducted at any locations outside of the United States?

Yes

Research conducted in foreign countries requires knowledge of local customs and cultural norms. Please review the International Research Guidance and Worksheet. Attach the completed worksheet to your submission. The International Research Guidance and Worksheet is not required if you are relying on an external IRB. In the attachments section, click International Research Guidance and Worksheet and select the Not Yet Available / Not Applicable checkbox.

All UNC students, faculty, and staff traveling internationally for this study are required to submit their itinerary to the Global Travel Registry.

Will your research project involve the Galapagos Islands, Ecuador?

No

If yes, your application will be reviewed by the UNC Center for Galapagos Studies. This Center will be included in routing for approvals after you submit.

2. Is UNC-CH the Lead Site or Coordinating Center or Sponsor of a multicenter project?

No Answer Provided

3. Is UNC-CH taking or being asked to take responsibility for the oversight of research by individuals, groups or organizations outside of UNC-CH?

No

Are you requesting that UNC-CH rely on an external IRB for continuing review and approval of this study?

No

Researchers are reminded that additional approvals may be needed from relevant "gatekeepers" to access subject.

Exemptions

Request Exemption

Some research involving human subjects may be eligible for an exemption which would result in fewer application and review requirements. This would not apply in a study that involves drugs or devices, involves greater than minimal risk, or involves medical procedures or deception or minors, except in limited circumstances.

1. Would you like your application evaluated for a possible exemption?

No

Scientific Review

Scientific Review

All biomedical research conducted at the University of North Carolina at Chapel Hill involving procedures that pose greater than minimal risk must undergo scientific review. Scientific review is a process that evaluates the scientific merit of a protocol ensuring that only scientifically sound protocols are submitted for IRB review. Scientific review ensures that the research uses procedures consistent with sound research design and the research design is sound enough to reasonably expect the research to answer its proposed question.

For example, research that involves experimental drugs or devices or invasive procedures requires scientific review. Additional examples can be found here.

At UNC, the Protocol Review Committee provides scientific review for oncology studies. For all other studies, scientific review can be conducted:

1. *Externally, by an independent organization that has no conflict of interest with the submitted research activity or;*
2. *Internally by the UNC Scientific Review Committee (SRC).*

Examples:

1. *An investigator-initiated study (with greater than minimal risk) regardless of the funding source must undergo scientific review by the UNC SRC unless it has been reviewed and approved by a protocol review committee empaneled for this purpose by Federal-funding agency (e.g., NIH, CDC, DOD) or a national foundation.*
2. *A multicenter industry/foundation sponsored protocol does not require scientific review by the UNC SRC.*

Note: Study section or FDA IND/IDE review is not adequate review to supplant review by the UNC SRC.

1. Does your research study require review by the Scientific Review Committee?
If you are unsure if your project requires SRC review, please contact the OCT at 919-843-2698.

Respond “no” if your research methods are limited to:

- the collection and/or use of existing data, documents or specimens and/ or,
- administering surveys; conducting interviews or focus groups, and/or
- prospective collection of biological specimens by non-invasive means (hair and nail clippings, sweat, dental plaque and calculus, saliva, sputum).
- Collection of blood from healthy adults by finger stick.

No Answer Provided

Part A. Questions Common to All Studies

A.1. Background and Rationale

A.1.1. Provide a summary of the background and rationale for this study (i.e., why is the study needed?). If a complete background and literature review are in an accompanying grant application or other type of proposal, only provide a brief summary here. If there is no proposal, provide a more extensive background and literature review, including references.

Finding effective strategies to reach out to young men and mobilize them to reduce their risk for sexually transmitted infections is critical, given men's control over the terms and conditions of most sexual partnerships. Unequal power distribution in relationships has a devastating impact on women, leading to HIV prevalence among young women in some sub-Saharan African countries four to seven times higher than among young men the same age. Gender power differentials have negative consequences for men as well, leading to increased risk of physical and mental health problems, substance use, and low uptake of health-related services.

We need innovative approaches to address the structural and social determinants of young men's risk. Lack of economic opportunity is a key structural determinant of risk that has negative consequences for men and has been linked to poor health outcomes.The influence of social network members is a social determinant of men's risk for both sexually transmitted infections and gender-based violence that can be addressed through interventions designed to change network norms. Research suggests that microfinance combined with health promotion can lead to improvement in health outcomes, including reductions in risk for sexually transmitted infections and gender-based violence.However, few, if any well designed evaluations of microfinance and health programs with young men have been reported. Two specific aims of the proposed study are:

A.1.2. State the research question(s) (i.e., specific study aims and/or hypotheses).

Specific Aim 1: To enumerate and characterize camps where young men at risk for HIV acquisition and transmission socialize in Dar es Salaam using the PLACE (Priorities for Local AIDS Control Efforts) methodology, a venue-based sampling approach. **Overview:** Trained staff in Tanzania will map and characterize all camps within four wards (equivalent to US census tracts), and describe men in camps through surveys with camp leaders and members. The PLACE assessment will provide us with a pool of camps from which we can randomly select those for trial inclusion.

Specific Aim 2: To determine whether men in camps randomized to receive a microfinance and health leadership intervention have a lower incidence of sexually transmitted infections (NG, TV, CT, HSV-2) and report perpetrating less physical or sexual violence against female sexual partners as compared to men in camps not randomized to receive the intervention. **Aim 2.1**: In the event that program effects are identified, to assess the degree to which hope, future orientation, perceived social support, collective efficacy, and social network norms mediate the impact of the multi-level intervention on STI and gender-based violence. **Overview:** Fifty-four camps in Dar es Salaam will be randomized to receive two years of a combined microfinance and health leadership intervention or a control condition, with all men in those camps receiving either the intervention or the control condition. Trained study interviewers in Tanzania will conduct quantitative and qualitative assessments at baseline, 12 months and 30 months.

A.2. Subjects

A.2.1. Total number of subjects proposed across all sites by all investigators (provide exact number; if unlimited, enter 9999):

2808

A.2.2. Total number of subjects to be studied by the UNC-CH investigator(s) (provide exact number; if unlimited, enter 9999):

2808

A.2.3. If the above numbers include multiple groups, cohorts, or ranges or are dependent on unknown factors, or need any explanation, describe here:

No Answer Provided

A.2.4. Do you plan to enroll subjects from these vulnerable or select populations:
If you will include children, prisoners or nonviable neonates or neonates of uncertain viability, please check the appropriate category below and complete the additional sections.

You should check "Pregnant women" if you specifically intend to recruit women who are pregnant or are not excluding pregnant women in biomedical research that is greater than minimal risk. Do not check if you are conducting a survey of the general public or conducting secondary data analysis or chart review not aimed at pregnant women.

Only check UNC-CH Student athletes, athletic teams, or coaches if you have specific plans to enroll these subjects. This is not applicable for intramural or club sports. For definitions and guidance see SOP 1201: Vulnerable subjects in research.

- ✔ Children (under the age of majority for their location)

  Any minor subject who attains the age of majority during the course of the research study must provide consent as an adult, unless consent has been waived, which is requested in section D.3.1.
- ✘ Pregnant women
- ✘ Nonviable neonates or neonates of uncertain viability
- ✘ Prisoners, others involuntarily detained or incarcerated (this includes parolees held in treatment centers as a condition of their parole)

  If an enrolled participant becomes incarcerated during the course of the research, they must be removed from the research project until such time as the IRB (and OHRP for NIH funded projects) approves the study to include prisoners, unless there is an immediate risk to the participant from ending treatments under the protocol.
- ✘ UNC-CH Student athletes, athletic teams, or coaches

A.2.5. Based on your recruitment plan and target sample population, are you likely to include any of the following as subjects? Select all that apply. This is not applicable to secondary data analysis or chart review.

Based on your responses, the consent form builder will insert the required text into your consent form template.

- ✘ Decisionally impaired individuals

  (e.g., Mini mental state examination (MMSE), Montreal cognitive assessment (MOCA))
- ✘ Children who are wards of the State (Foster children)
- ✔ Non-English-speaking individuals
- ✘ UNC-CH Students
- ✘ UNC-CH Employees
- ✔ People, including children, who are likely to be involved in abusive relationships, either as perpetrator or victim.

  This would include studies that might uncover or expose child, elder or domestic abuse/neglect. (See SOP Appendix A)

A.2.6. If any of the above populations are checked (excluding 'Decisionally impaired individuals' and 'Children who are wards of the State (Foster children)'), please describe your plans to provide additional protections for these subjects.

As we did in the study that provides the preliminary evidence for this trial (IRB # 08-0502), we will request a waiver of parental consent to include men 15-21 years on the basis that under 45 CFR § 46.408 (c), an IRB has the authority to waive parental permission if it determines that "a research protocol is designed for conditions or a subject population for which parental or guardian permission is not a reasonable requirement to protect the subjects" and "an appropriate mechanism for protecting the children who will participate as research subjects is substituted" and "that the waiver is not inconsistent with Federal, State, or local law." This population has an evolving capacity for self-determination that is proportional to the level of risk inherent in this study. This population of youth and the conditions in which they live and in which we would access them make obtaining parental permission for their participation not practical. Adequate protection has been substituted in the anonymity of the interviews and the referrals for services if needed.

A.2.7. Age range of subjects:

Minimum age of subject enrolled	15
	years
Maximum age of subject enrolled	99
» If no maximum age limit, indicate 99	
	years

A.3. Inclusion/exclusion criteria

A.3.1. List required characteristics of potential subjects (i.e., inclusion and exclusion criteria). If not covered, list also characteristics that would preclude their involvement.

Study participants. Study participants will be male members of camps within the four wards. To verify that men are members of a camp we will collect camp rosters and check names and dates of birth for each individual. An individual has to be a registered member of a camp to participate. All camps have rosters. The age range of camp members from our previous assessment was 15-40 years. All registered male camp members will be included in this trial. We anticipate very little cross-over of camp members within a ward based on results from our previous research. Less than 2% of the men reported they belonged to more than one camp.

Screening inclusion and exclusion criteria. The project will be introduced to the camp as a whole after we have permission of camp leaders to work within the camp. Individual men within the camp will then be asked for his consent to participate. For those who are willing to participate, the interviewers will conduct written informed consent and complete a short eligibility screening form. Screening will be conducted in a private space in or near the camp. *Inclusion criteria:* To be eligible participants must meet the following criteria: 1) be a registered camp member for at least 3 months; 2) plan to reside in Dar for the next 30 months; 3) be 15 years or older; 4) be of male gender. *Exclusion criteria:* Individuals who meet the following criteria will be excluded: 1) unwilling to provide locator information; 2) unable to participate due to psychological disturbance, cognitive impairment or threatening behavior.

A.3.2. Justify any exclusion based on race, gender or ethnicity

Camps are primarily male organizations as we discovered from our NIMH R21 grant. Among the 73 camps we surveyed the proportion of camp members who were female ranged between 0 and

17%. We will only include men in this intervention trial for two reasons. First, the intervention is designed to address the structural and social determinants of risk among men, and our pilot work provides evidence only for men. Second, the small numbers of women in camps preclude our ability to achieve sufficient power to detect program effects among them.

A.3.3. Will pregnant women or women who become pregnant be excluded?

No

A.4. Study design, methods and procedures

Your response to the next question will help determine what further questions you will be asked in the following sections.

A.4.1. Will you be using any **methods or procedures commonly used in biomedical or clinical research** (this would include but not be limited to drawing blood, performing lab tests or biological monitoring, conducting physical exams, administering drugs, or conducting a clinical trial)?

Yes

A.4.2. Describe the study design. List and describe study procedures, including a sequential description of what subjects will be asked to do, when relevant.

Study Procedures

PLACE assessment. The first phase of this study will involve conducting a PLACE (Priorities for Local AIDS Control Efforts) assessment within four wards of Dar es Salaam to enumerate camps eligible for inclusion in the trial. We will be working in Kinondoni district, one of three districts in Dar es Salaam. We selected Kinondoni because it is the most populous (over 1 million) and most impoverished of the districts. 56 Kinondoni has 29 wards (analogous to U.S. census tracts). We have selected four wards characterized by high risk for HIV among young people. These four include Manzese (population 66,543), Tandale (population 44,853), Mwananyamala (population 44,344), and Mabibo (population 77,639). In all four wards over a third of the population are males aged 15-29 (range among four wards is 36-38%). 56 Illicit drug use, alcohol use, and commercial sex are common to the four wards. In one study of HIV infection among drug users, 66.7% of syringes from male injectors in Manzese and Tandale tested HIV positive and 40% of syringes from male injectors in Mwananyamala tested HIV positive. The PLACE assessment includes the following steps in these 4 wards: 1) Enumerate camps. Trained local staff will conduct brief structured interviews with community informants to identify the camps. The goal is to interview as many people as necessary within each ward until there is saturation in the venue lists. Based on previous PLACE assessments, investigators found that selecting 4 informants per 1,000 people was sufficient to develop saturated venue lists. The population of the four wards where we will work ranges from 44,000 to 77,000, so trained staff will interview between 176 and 308 informants per ward. Interviewers will record the name and address of each venue identified by the informants onto a Venue Reporting Form (Appendix A). 2) Characterize the camps within each ward. In this step of PLACE, the interviewers will complete the venue verification form with informants who are knowledgeable about each camp (Appendix B). In our pilot study this step included camp leaders and/or camp members. This form includes information on the gender and age of informants, and on the venues themselves. Interviewers will collect coordinates of camps using a global positioning device (GPS) so that we can position camps on a ward map to record the location and distance between camps.

Enrollment and randomization. After local staf have enumerated, mapped and verified all camps in the four wards, we will randomly select camps for inclusion in this trial. Trained interviewers will complete the enrollment and baseline visit with camp members in the selected camps prior to assignment of camps to study arms. Study interviewers will obtain informed consent, collect locator information on each camp member to help with tracing, and complete the baseline behavioral survey. These staff will be accompanied by trained nurses who will conduct the screening for

sexually transmitted infections. All these steps were carried out successfully in the R21-funded pilot study. Following enrollment and baseline surveys, the camps will be randomly assigned to one of the two study arms. To promote the transparency of the randomization process, we will hold a meeting of camp leaders from the 54 selected camps. We will prepare sealed envelopes with randomization assignment cards. One leader from each camp will be invited to come forward and select an envelope with the camp assignment.

Participation rates, retention and tracing procedures. In our previous work we had a very high participation rate of men from the camps. We were able to interview over 99% of the eligible men (670/675). We anticipate similar participation in this trial. At enrollment into this study, staff will record locator information (name, telephone number, typical place of residence or sleeping location) and names of contacts who usually know where to find the participant, with an assurance not to reveal the participant's involvement in the study. The following systems are in place at the site to maintain cohort retention: (1) A well-designed computerized follow-up program which we have used before will track all scheduled daily appointments and missed appointments together with contact information; (2) Locator information will be updated every study visit; (3) Study participants who miss scheduled appointments will be contacted within 24 hours by telephone or by home visit if they did not leave a telephone contact. If participants have changed their address, maximum information is gathered (while maintaining participant confidentiality) from the participant's named contacts for further contact. If participants are not able or do not wish to attend a study visit, reasons for non-attendance will be recorded. Participants are considered lost to follow-up if multiple telephone contacts and 5 home visits fail.

Pre-test survey. Prior to launching the baseline survey study interviewers will pre-test all survey items and Computer-Assisted Personal Interview (CAPI) procedures with fifty camp members who have similar demographic characteristics to our target population but are not members of an intervention or control camp. The reasons for this pre-test is to assess length of the survey, check response distribution, assess item comprehension, and pilot data collection procedures.

Baseline survey. Camp members in the 54 camps will participate in a survey assessing socio-demographics, outcome variables and mediating variables. See Appendix C for the survey we used in our pilot study. The questionnaires will be administered using CAPI by trained interviewers who are not intervention facilitators. The interview will last approximately 60 minutes. Our pilot work in camps demonstrated the feasibility of conducting interviews of this length. We had no camp member refuse the interviews because of the length. Participants who appear influenced by drugs will be asked to return for an interview the next day.

STI assessment. Study nurses will collect urine and blood specimens for diagnosis of all STIs including NG, TV, CT and HSV-2 at baseline and 30 months. They will identify a private space for physical exams and specimen collection at or near each camp.

Follow-up behavioral assessment. At 12 months and 30 months a follow-up behavioral assessment will be conducted among participants in order to assess outcome and mediating variables. Study interviewers will use the same procedures described for the baseline assessment.

Qualitative cohort. At baseline, 12 months and 30 months study interviewers will conduct qualitative interviews with a sub-sample of 2 men from each of the 27 camps in the intervention arm. At baseline they will ask men about their relationships, including sexual, family and camp member relationships, about their current employment situation and their aspirations for the future. At 12 and 30 months the interviewers will ask the same men about their experiences with the intervention including their business development, how they have used the money they have earned from the business, as well as conversations they have had with the health leaders in their camp. The interviewers will revisit their goals and hope for the future and follow up with questions related to their sexual, family and camp relationships.

Intervention implementation

Intervention components. Our intervention is a multi-level HIV/STI prevention trial that will be implemented over a two year period and jointly addresses both structural and social factors that contribute to HIV/STI risk and partner violence. **4.4.3.1 Microfinance.** We will partner with the Youth Self-Employment Foundation (YOSEFO), a microfinance institution in Dar es Salaam, to implement the microfinance component of the intervention (www.yosefo.org). YOSEFO partnered with us to implement the pilot for this trial. YOSEFO has been providing loans to young men and women in Tanzania since1996. As of 2011, the total number of YOSEFO clients was 18,200, with an outstanding loan amount of 3.9 billion Tanzanian Shillings ($2.3million USD). The standard principles and practices in microfinance that successfully guided our pilot study and will guide implementation of this study include: (1) Business skill training: Camp members will receive a 2-week business training workshop facilitated by YOSEFO, and two booster training sessions. The trainings will focus on how to generate viable business ideas, how to assess markets, how to start and scale up businesses and how to manage them once they are started. 2) Formation of loan groups: As is standard microfinance practice, camp members will be required to form groups of 5 for the purpose of receiving the loans. While loans are paid to individuals, the groups are accountable for repayment of all group members' loans, such that if one member misses any payment, the other members of the group must repay on his behalf. 3) Applications for loans: Individuals apply for a loan by completing a loan application form that includes details on the planned business, information on group members, and evidence that the individual has 12.5% of the loan in savings. 4) Distribution of loans. After successful completion of the application camp members will receive an initial loan of $100 USD. 5) Weekly loan repayment sessions: The camp members will meet weekly with a loan officer for repayment sessions. 6) Ongoing access to credit: Ongoing access to incrementally larger loan amounts is a key feature of microfinance. After successfully completing the first loan cycle, camp members will be eligible for a second loan in the amount of $200 USD. **4.4.3.2. Health Leadership.** The second component of the intervention is a health leadership intervention that will target peer leaders within their camps as health promoters for behavior change. This intervention component builds on the fact that it is possible to identify leaders within the camp who are respected and trusted by their peers. To implement this intervention component we will take the following steps: (1) *Nomination process* . To identify peer leaders we will use a process that we implemented during the pilot intervention. The Project Coordinator in Tanzania will hold an initial meeting with male camp members at each camp. He/she will ask them to identify qualities of leaders, and then to each identify through a confidential process up to 5 male members within the camp whom they feel best demonstrate these qualities. We will aggregate the data from the nomination forms so we identify 10 individuals in each camp who receive the most nominations. Once we have identified the leaders, we will approach those individuals and ask if they would be interested in being trained as peer leaders for their camp. Within the pilot none of the leaders that we invited to participate in the training refused. (2) *Training.* We have developed a training manual for peer leaders that we used in the pilot intervention that we will use in this trial (Appendix D). The manual focuses on building skills in effective communication and social influence. The training will provide camp leaders with knowledge and will address myths and misconceptions related to HIV transmission, condoms, violence, and multiple partnerships. The feedback leaders provided in the training evaluation during our pilot indicated that they learned new information and found the skill-building helpful. (3) *Implementation of strategies.* Once leaders have been trained, they will be asked to implement the communication and social influence strategies they learned. The leaders will record information about the number of times they engage in peer discussions about the issues discussed in training. They will record the different strategies they used, and reflections on what worked and what did not work for them. 4) Conducting booster training sessions. The Intervention Coordinator in Tanzania will conduct follow-up training sessions for the leaders at 6 and 12 months after the intervention has launched so the leaders across camps can share experiences and continue to build their skills.

Control condition. Twenty-seven camps will be randomized to a control condition. The control condition will focus on nutrition and physical activity. We will follow WHO recommendations for the promotion of physical activity and nutrition in developing country settings. Staff in Tanzania will use print material to raise awareness of the importance of regular physical activity and

a healthy diet. The print material will be distributed to camp members at baseline and at 6 and 12 months when booster trainings are being conducted for health leaders in intervention camps.

A.4.3. If subjects are assigned or randomized to study "arms" or groups, describe how they are assigned.

- *Describe the methods of computing the randomization schedule (if any) and maintaining blinding (if any).*
- *Who will perform these computations?*
- *How will you verify each subject's eligibility prior to randomization?*

This is a cluster-randomized trials, and as such camps will be randomized to the intervention or the control arm. Following enrollment and baseline surveys, the camps will be randomly assigned to one of the two study arms. To promote the transparency of the randomization process, the local PI in Tanzania will hold a meeting of camp leaders from the 54 selected camps. We will prepare sealed envelopes with randomization assignment cards. One leader from each camp will be invited to come forward and select an envelope with the camp assignment.

A.4.4. Describe any follow up procedures.

Follow-up behavioral assessment. At 12 months and 30 months a follow-up behavioral assessment will be conducted among participants in order to assess outcome and mediating variables. Trained study interviewers in Tanzaia will use the same procedures described for the baseline assessment.

Qualitative cohort. At baseline, 12 months and 30 months study interviewers will conduct qualitative interviews with a sub-sample of 2 men from each of the 27 camps in the intervention arm. At baseline interviewers will ask men about their relationships, including sexual, family and camp member relationships, about their current employment situation and their aspirations for the future. At 12 and 30 months interviewers will ask the same men about their experiences with the intervention including their business development, how they have used the money they have earned from the business, as well as conversations they have had with the health leaders in their camp. Interviewers will revisit their goals and hope for the future and follow up with questions related to their sexual, family and camp relationships.

A.4.5. Once this study has been approved by the IRB, for how many months or years will this study be active (you are collecting data or have access to identifiers)?

The entire study will last 5 years.

A.4.6. Will this study use any of the following methods?

✔	Audio Recording
✘	Video Recording
✘	Behavioral observation - (e.g., Participant, naturalistic, experimental, and other observational methods typically used in social science research)
✘	Pencil and paper questionnaires or surveys
✘	Electronic questionnaires or surveys
✘	Telephone questionnaires or surveys
✔	Interview questionnaires or surveys
✘	Other questionnaires or surveys

- ✗ Focus groups
- ✗ Diaries or journals
- ✗ Photovoice
- ✗ Still photography

A.4.7. If there are procedures or methods that require specialized training, describe who (role/qualifications) will be involved and how they will be trained.

Trained nurses will work with the study interviewers during the period of baseline and post-intervention data collection to collect the blood and urine specimens from the young men. The nurses we select will be trained in proper specimen collection and storage.

A.4.8. Are there cultural issues, concerns or implications for the methods to be used with this study population?

No

A.4.A. Biomedical methods and procedures

A.4.A.1. Is this an interventional study?

Yes

Distinguish what is being done specifically for this research from procedures that would be done anyway for clinical care:

Treatment that will be provided for the STIs will be according to the standard of care for Tanzania.

A.4.A.2. Is this a Clinical Study?
Check YES if this study involves research using human volunteers that is intended to add to medical knowledge. There are two main types of clinical studies: clinical trials and observational studies. Do NOT check yes merely because you are conducting research in a clinical setting or using clinical data.
Click here for additional definition of "Clinical Study"

No

A.4.A.3. If the study involves the use of placebo control, provide justification

The study is a cluster-randomized trial. The unit of randomization are camps, which are semi-formal organizations of youth. Camps will be randomized to the intervention or a control arm. The intervention arm consists of a combination of microfinance and health leadership promotion for men in camps. The control condition consists of an intervention to promote physical activity and nutrition. The use of a control condition is justified in the case of this trial because we do not know whether the proposed intervention will be any more efficacious than the control condition at affecting the primary outcomes we are interested in measuring for this study.

A.4.A.4. Will this study involve drugs, biologics or other substances (such as a botanical or dietary supplement)?
For guidance on dietary supplements, see Section VI, C FDA guidance document UCM229175.pdf

No

A.4.A.5. Is there an Investigational New Drug application (IND) for this study?

No Answer Provided

A.4.A.6. When the intent of a clinical investigation is to collect information about the safety or effectiveness of a device, the need for an Investigational Device Exemption (IDE) must be evaluated. Please review the Investigational Device Guidance document prior to completing this section. Your response to the following questions will determine if an IDE is needed.

A. Select the response that best describes your investigation:

No Answer Provided

A.4.A.7. Does your study involve any of the following? (check all that apply)

- ✗ Embryonic stem cells
- ✗ Fetal tissue
- ✗ Genetic testing (see GINA and GWAS)
- ✗ Clinical laboratory tests

If McLendon Labs will do the testing, you must complete the appropriate form found at UNC Health Care and submit to them for review.

- ✗ Testing for communicable diseases that have mandated reporting requirements (link to state guidance)
- ✓ Point of Care Testing (POCT), which is CLIA-approved testing done at the "bedside" or site of care by hospital or clinic personnel (not by subject). Examples include urine pregnancy testing, glucose monitoring, etc.

If McLendon Labs will do the testing, you must complete the POCT form found at UNC Health Care and submit to them for review.

- ✗ If your study utilizes **radiopharmaceuticals** to address basic science questions, an IND is not necessary.
  Instead, your study will be reviewed/approved by the Radioactive Drug Research Committee (RDRC); approval by the Radiation Safety Subcommittee (RSS) is not required.
  If you have questions about the RDRC approval process, please contact Dede Corvinus.
- ✗ Diagnostic or therapeutic ionizing radiation, or radioactive isotopes (not covered under 21 CFR 361.1), which subjects would not receive otherwise if not participating in this research study. Do not check if all radiation is administered as standard of care. Do check if your study includes views/scans that represent no greater than minimal risk as determined by the Radiation Safety Sub-committee.
  Application for Human Use of Radiation in Research.
- ✗ Gadolinium administered as a contrast agent
- ✗ Recombinant DNA or gene transfer to human subjects
- ✗ Any research activities conducted in the UNCHC Perioperative areas. This includes Pre-care, Pre-op, Operating room and PACU.
  You must complete the Checklist for Perioperative Services and return it to moe_lim@med.unc.edu
- ✗ Any form of medical imaging (ultrasound, MRI, CT, X-ray, PET-CT, PET-MRI)

A.4.A.8. Will your study involve storage of specimens for future unspecified research?

No

A.5. Benefits to subjects and/or society

A.5.1. Describe how this study will contribute to generalizable knowledge that will benefit society.

Participants may benefit from knowing that the information learned from this study will help to improve the situation of youth like themselves. The participants may also benefit from the testing and treatment of sexually transmitted infections that will be offered at baseline and in the post-intervention assessment. Any participant who is found to be positive for any STI will be offered treatment at no cost. The youth may also benefit from talking with the trained peer leaders about social concerns that they face. In terms of social benefits, if the trial is successful there are potentially enormous social benefits that can be gained from the study. We expect this intervention to be effective, cost-effective, sustainable and easily disseminated in other developing country settings. Youth in sub-Saharan Africa and elsewhere continue to be disproportionately affected by the HIV epidemic, and there are limited strategies that have proven effective at reaching youth. If proven efficacious, we feel this multi-level approach to reaching young men at risk for HIV transmission and acquisition will have widespread application in other settings.

A.5.2. Does this study have the potential for direct benefit to individual subjects in this study?

Yes

Consider the nature, magnitude, and likelihood of any direct benefit to subjects. If there is no direct benefit to the individual subject, say so here and in the consent form, if there is a consent form. Do not cite monetary payment or other compensation as a benefit.

Explain

Yes, the communication of information about sexually transmitted infection results is a benefit to the participants. In addition, men who test positive for any STI will be provided with treatment free of charge through our study.

A.5.3. Are there plans to communicate the results of the research OR results of any clinical tests administered for the research back to the subjects?

Yes

If yes, describe

We plan to establish a community advisory board for this project. We will work through the community advisory board to establish the best procedures to communicate study progress and study results back to the community.

A.6. Risks and measures to minimize risks

For each of the following categories of risk you will be asked to describe any items checked and what will be done to minimize the risks.

A.6.1. Psychological

- ✔ Emotional distress
- ✔ Embarrassment
- ✔ Consequences of breach of confidentiality (Check and describe only once on this page)
- ✘ Other

A.6.2. Describe any potential psychological risks checked above and what will be done to minimize these risks

We believe the risk of participants experiencing these negative psychological events as a result of participation in the study will be infrequent. It is possible that some participants may experience these outcomes as a result of responding to the survey questions or participation in the health promotion componment of the intervention trial. We will train our interviewers in techniques of interviewing to help minimize the embassessment and emotional distress that men may feel responding to person questions about their relationsihps. Health leaders from camps will also be trained in techniques to help minimize the embarrassment that men may feel as a result of participating in the health promotion component of the study. It is also possible that men may experience some social or psychological risks if they learn they are positive for a sexually transmitted infection. Nurses who will take the specimens and provide the results to the men will be trained in appropriate counseling procedures to communicate and discuss results with the men. If men have social or psychological concerns beyond what nurses feel they are capable of responding to the nurses will be traned to refer these men to the site PI, Lusajo Kajula, who is a trained psychologist with experience working with adolescents.

A.6.3. Social

- ✔ Loss of reputation or standing within the community
- ✘ Harms to a larger group or community beyond the subjects of the study (e.g., stigmatization)
- ✔ Consequences of breach of confidentiality (Check and describe only once on this page)
- ✘ Other

A.6.4. Describe any potential social risks checked above and what will be done to minimize these risks

It is possible, though we feel it will be rare, that men may experience some negative social outcomes as a result of participating in this study. To minimize these social risks we will engage a community advisory board who will help us inform the community of the purpose of our study, and address any rumors or negative social interactions that may occur in the community related to the trial. We also will not use any identifying information on study data. All men will be assigned a unique study identification number. These numbers will be used on all study documents. The list that we keep that links men's names to these study identification numbers will be kept locked in a filing cabinet in the Project Director's office.

A.6.5. Economic

- ✘ Loss of income
- ✘ Loss of employment or insurability
- ✘ Loss of professional standing or reputation
- ✘ Loss of standing within the community
- ✘ Consequences of breach of confidentiality (Check and describe only once on this page)
- ✘ Other

A.6.6. Describe any potential economic risks checked above and what will be done to minimize these risks.

No Answer Provided

A.6.7. Legal

- ✗ Disclosure of illegal activity
- ✗ Disclosure of negligence
- ✗ Consequences of breach of confidentiality (Check and describe only once on this page)
- ✗ Other

A.6.8. Describe any potential legal risks checked above and what will be done to minimize these risks

No Answer Provided

A.6.9. Physical

- ✗ Medication side effects
- ✓ Pain
- ✗ Discomfort
- ✓ Injury
- ✗ To a nursing child or a fetus (either through mother or father)

A.6.10. Describe any potential physical risks checked above, including the category of likelihood and severity, and what will be done to minimize these risks. Where possible, describe the likelihood of the risks occurring, using the following terms:

- Very Common (approximate incidence > 50%)
- Common (approximate incidence > 25 - 50%)
- Likely (approximate incidence of > 10 - 25%)
- Infrequent (approximate incidence of > 1 - 10%)
- Rare (approximate incidence < 1%)

Describe severity of risks using the following grading scale:

- Mild- No disruption to the subject's ability to perform daily activities; may include non-prescription intervention only
- Moderate- Temporary interference with daily activities; may include prescription intervention
- Severe- Interference with daily activities; medically significant but not life threatening
- Life threatening

Examples:

Rare (< 1%) and Severe: blindness
Rare (< 1%) and Mild: dry skin, dry mouth, transient headache

If you are using these terms differently than described above, please provide your study-specific definitions.

Phase 1 trials: Due to limited experience, incidence may be better described as the number of events that have occurred in the total number of animals/humans studied.

There may be risks of pain or injury associated with the blood draws. We will work with nurses who are trained in phlebotomy in order to minimize these risks.

A.6.11. Unless already addressed above, describe procedures for referring subjects who are found, during the course of this study, to be in need of medical follow-up or psychological counseling

Men who test positive for any STI will be offered treatment at no cost through our study. If men in our study require psychological counseling we will have procedures in place to insure that they get the services that they require. Our collaborators in Tanzania are faculty based in the Department of Psychiatry at Muhimbili University of Health and Allided Sciences. They are trained in psycholgoical and psychatric medicine. Thus if any interviewer or study staff member assesses that a participant requires psychological counseling they will be referred directly to our study PI, who is a trained Psychologist. If a participant requires medical attention, our PI and co-Invesigators have colleagues the hospital where the university is based who will make themselves available to assist the partiicpant at no cost.

A.6.12. Are there plans to withdraw or follow subjects (or partners of subjects) who become pregnant while enrolled in this study?

No

A.7. Data and safety monitoring

A.7.1. When appropriate, describe the plan for monitoring the data to ensure the safety of participants. These plans could range from the investigator monitoring subject data for any safety concerns to a sponsor-based data and safety monitoring board or committee (DSMB, DSMC, DMC), depending on the study. For studies that do not raise obvious safety concerns, you may still describe your plans for monitoring the study as it progresses.

Data Safety and Monitoring Plan: Our data safety and monitoring plan involves the following steps:

1) Event reporting: All serious adverse events associated with the procedures will be appropriately reported to the UNC IRB and the MUHAS IRB. Field staff in Tanzania will be trained to complete descriptions of adverse events that will then be sent electronically to both the U.S. PI and the Tanzanian PI. Thus, adverse events will be monitored at three levels; by the Principal Investigators, by the UNC IRB and by the MUHAS IRB. Of course, efforts will be taken to minimize the potential for adverse events. Intervention and research staff training will stress ensuring confidentiality and avoidance of negative events, and quality assurance/quality control (QA/QC) will be performed regularly to ensure adherence to proper counseling techniques. Two types of reports will be made involving the conduct of the study.

Adverse Events Reports will be made using standard forms available from the relevant IRBs for adverse events associated with the study procedures or subject participation. Serious medical adverse events are unlikely as the only medical procedure that will be conducted as part of the assessment is the diagnosis for sexually transmitted infections.

Incident Reports will also be made of any incidents involving the conduct of the trial (eg, enrolling a participant who did not meet eligibility criteria, needle stick injuries of staff, etc.) These reports will be made in the form of a letter or memo to the Chairs of the relevant IRBs signed by the Principal Investigators.

Copies of adverse event reports will be stored at UNC and in the study offices at the Muhimbili University of Health and Allied Sciences. Adverse events or incident reporting in this trial has been considered in the context of several important characteristics of the study. First, this is a minimal-risk study as defined in federal regulations—"the probability and magnitude of harm or discomfort anticipated in the research are not greater in and of themselves than those ordinarily encountered in daily life or during the performance of routine physical or psychological examinations or tests." Second, although no clear guidance is currently provided in regulation for adverse events reporting in behavioral studies such as this one, as opposed to clinical trials of drugs or devices, we are taking the steps outlined above within the context of our study.

2) Contact information for participants: We will provide participants with a palm card containing

information on how to contact the local research staff to report such events as breaches of confidentiality, HIV-related disruption of families, acts of discrimination, and physical harm. We will ask participants to return to the research site or otherwise contact research staff in order to make such reports as well as receive referrals to mitigate potential harm. Palm cards will not include identifying information about the study or references to HIV or HIV testing, so that the cards will not have the potential to jeopardize the confidentiality of participants.

3) Protection for staff: If staff members experiences occupational exposure to HIV, the incident will be reported and a protocol will be followed to minimize their risk of being infected with HIV. All staff at risk for occupational exposure to HIV will be trained on universal precautions and on the post-exposure prophylaxis protocol. All staff performing HIV tests will require training and certification on performing the test and on quality assurance.

4) Human subjects training for staff: All study staff/volunteers having potential contact with study participants or data will receive human subjects training. All staff will complete the required online human subjects training for certification as well.

A.7.2. If not already addressed above, describe the plans for aggregate review of unanticipated problems (including but not limited to adverse events) across all sites, in order to monitor subject safety.

See above.

A.7.3. What are the criteria that will be used to withdraw an INDIVIDUAL SUBJECT from this study or halt the research intervention (e.g., abnormal lab tests, allergic reactions, failure or inability to comply with study procedures, etc.)?

If a participant fails to attend study appointments despite repeated attempts by our study staff to remind them of this apointment, they may be exited from the study. If a participant becomes disruptive either duringa study interview or during an intervention activity, to the point where it disrupts the activity for other participants and puts study staff and/or other participants at potential harm, they will be exited from the study.

A.7.4. Are there criteria that will be used to stop the ENTIRE STUDY prematurely (e.g., safety, efficacy, unexpected adverse events, inability to recruit sufficient number of subjects, etc.)?

Yes

Please explain

We will monitor the adverse events and in consultation with the IRB, we will stop the study prematurely if there is evidence that the study has lead to the adverse events.

A.7.5. Will this study involve a data and safety monitoring board or committee?

No

A.8. Data analysis

A.8.1. Describe the analytical methods to be used (qualitative or quantitative)

Analytical Techniques. We will compare odds ratios across group at the 12 month assessment for behavioral outcomes and at 30 months for both behavioral and STI outcomes using SAS SURVEYLOGISTIC version 9.2 to incorporate both the complex nature of sample selection and weighting. Controls for covariates will also be introduced where necessary. We will estimate path coefficients in multiple mediator models and generate bootstrapped confidence intervals for total and specific indirect effects of one or more mediator models using the Mplus program Version 6.11.

We will assess simple and multiple mediation in the relationships between the microfinance intervention condition, intervention mediators including hope, future orientation, perceived social support, collective efficacy, and social network norms for partner violence, condom use, and concurrency and intervention outcomes of: STI risk, HIV testing uptake, condom use, # partners, sexual concurrency, and physical and sexual violence.

Qualitative data analysis: All in-depth interviews with men from camps will be audio-taped, transcribed, translated, coded and computerized for analysis. Analysis will begin during data collection so that topics for further exploration can be incorporated into ongoing fieldwork. Qualitative data analysis consists of searching for patterns in data and conceptualizing ideas that help explain the presence of those patterns.87 Analysis of textual data will involve five steps: (1) reading for content; 2) deductive and inductive coding; 3) data display to identify emerging themes; 4) data reduction; and 5) interpretation.

A.8.2. Explain how the sample size is sufficient to achieve the study aims. This might include a formal power calculation or an explanation of why a small sample is sufficient (e.g., qualitative research, pilot studies)

Sample Size, Power, and Effect Size. The choice of 54 camps randomized into two groups is based upon evidence generated in our previous study and likely incidence estimates available from published studies with similar populations in Tanzania and other countries in Africa. We estimate average camp sizes of 36.2 sexually active males and 52 total males. STI measures of ulcer, discharge, any symptom, and any STI were used as possible sexual outcome measures, with condom use and sexual and/or physical violence perpetration our behavioral outcome measures. Based upon our previous study we computed intraclass correlation (ICC) estimates between 0.00 and 0.01 for sexually transmitted diseases and 0.00-0.032 for behavioral measures. Given average camp sizes and ICC estimates we have inflated sample size estimates assuming simple random sampling with design effects ranging from 1.4 to 2.3. All sample estimates assumed an attrition of 20%. Given a sample size of 1955 sexually active males and 2808 total males in 54 camps we will have 80% power (2-sided, α=0.05) to detect the protective effect of the intervention (OR) of 0.32 for ulcer, 0.59 for discharge, 0.61 for any STI symptom, 0.50 for syphilis, and 0.66 for any STI. We will be able to detect protective effects of 0.74, 0.68 and 0.65 in condom use for those sexually active, for all males, and for sexual or physical violence perpetration in all males, respectively. Statistical power to detect mediation is expected to be high based on the simulation results of Fritz and MacKinnon where a total sample size of 539 was sufficient for .80 power to detect the most conservative simulated mediation effect.

A.9. Identifiers

A.9.1. Check which of the following identifiers you already have or will be receiving, or select "None of the above."

- ✔ Names (this would include names/signatures on consent forms)
- ✔ Telephone numbers
- ✔ Any elements of dates (other than year) for dates directly related to an individual, including birth date, admission date, discharge date, date of death. For ages over 89: all elements of dates (including year) indicative of such age, except that such ages and elements may be aggregated into a single category of age 90 and older
- ✔ Any geographic subdivisions smaller than a State, including street address, city, county, precinct, zip code and their equivalent geocodes (e.g. GPS coordinates), except for the initial three digits of a zip code
- ✘ Fax numbers

- ✗ Electronic mail addresses
- ✗ Social Security numbers
- ✗ Medical record numbers
- ✗ Health plan beneficiary numbers
- ✗ Account numbers
- ✗ Certificate/license numbers
- ✗ Vehicle identifiers and serial numbers (VIN), including license plate numbers
- ✗ Device identifiers and serial numbers (e.g., implanted medical device)
- ✗ Web universal resource locators (URLs)
- ✗ Internet protocol (IP) address numbers
- ✗ Biometric identifiers, including finger and voice prints
- ✗ Full face photographic images and any comparable images
- ✗ Any other unique identifying number, code, or characteristic, other than dummy identifiers that are not derived from actual identifiers and for which the re-identification key is maintained by the health care provider and not disclosed to the researcher
- ✗ None of the above

A.9.2. For any identifiers checked, how will these identifiers be stored in relationship to the research data?

- ✗ with the research data (i.e., in the same data set and/or physical location)
- ✓ separate from the research data (i.e., coded with a linkage file stored in a different physical location)

Provide details about the option you selected above:
We will keep a separate list that links the unique ID number with the personally identifying information. This list will be stored in a locked filing cabinet in the Project Director's office.

A.9.3. Are you collecting Social Security Numbers to be used as a unique identifier for study tracking purposes for national registry or database? (Do not check yes if collecting SSN *only* for payment purposes; this will be addressed later.)

No

A.10. Confidentiality of the data

A.10.1. Describe procedures for maintaining confidentiality of the data you will collect or will receive (e.g., coding, anonymous responses, use of pseudonyms, etc.).

Confidentiality: Successful implementation of the trial will require that strict confidentiality of all study participants be maintained. For the survey and qualitative data collected as part of the study, participants will be assigned a unique identification number. All data and information collected will be kept in locked filing cabinets. Counselors, interviewers, data entry clerks, data manager and tracers will all be trained on procedures to maintain confidentiality and will be required to sign an oath of confidentiality prior to study implementation. We will ask participants to return to the research site as well as provide them with a card that has contact information for research staff to report such incidents as HIV-related disruption of family, as well as physical and social harms. This will enable research staff to make appropriate referrals to community-based agencies for ongoing support.

A.10.2. Describe how data will be transmitted among research team (i.e., personnel listed on this application).

Data will regularly be transmitted electronically through files that will be encrypted.

A.10.3. Are you collecting sensitive information such as sexual behavior, HIV status, recreational drug use, illegal behaviors, child/physical abuse, immigration status, etc?

Yes

If yes, describe the sensitive data being collected

Both survey and qualitative data will include sensitive information related to sexual behavior and gender-based violence.

A.10.4. Do you plan to obtain a federal Certificate of Confidentiality for this study? Please note that all ongoing or new research funded by NIH as of December 13, 2016 that is collecting or using identifiable information is automatically issued a Certificate of Confidentiality (CoC). You should also select “Yes” if your study is NIH funded and has been issued a CoC under this updated NIH policy.

No

A.10.5. If this study is limited to data collection by survey or interview, discuss the potential for deductive disclosure (i.e., directly identifying subjects from a combination of indirect IDs).

We don't believe that there will be a risk of deductive disclosure.

A.10.6. Will any of the groupings or subgroupings used in analysis be small enough to allow individuals to be identified?

No

A.11. Data sharing and transmission

A.11.1. Check all of the following who will receive **identifiable data** (contains any of the 18 identifiers listed above) outside the immediate research team (i.e., not listed as personnel on this application)? *

- ✔ No one
- ✘ Coordinating Center
- ✘ Statisticians
- ✘ Consultants
- ✘ Other researchers
- ✘ Registries
- ✘ Sponsor and/or its designee(s)
- ✘ External labs for additional testing
- ✘ Journals
- ✘ Publicly available dataset
- ✘ Other

If other, please specify.
There is nobody outside of our research team who will have this identifiable information. Members of the intervention team (such as the microfinance officers) may have access to men's phone numbers and details such as their dates of birth as part of the loan application and repayment process, but they will not have other information such as their physical addresses.

A.11.2. For any recipients checked above, explain the confidentiality measures to be taken

No Answer Provided

A.12. Post-study disposition of identifiable data or human biological materials

A.12.1. Describe your plans for disposition of data or human biological specimens that are identifiable in any way (directly or via indirect codes) once the study has ended. If you plan to destroy linkage codes or identifiers, describe how and when this will be done.

Study data will be stored electronically until all study analyses are completed. Biological specimens will be stored only until we are sure that all specimens have been processed, and data are clean.

Part B. Direct Interaction

B.1. Methods of recruiting

B.1.1. Check all the following means/methods of subject recruitment to be used:*

- ✔ In person
- ✘ Join the Conquest
- ✘ Participant pools
- ✘ Presentation to classes or other groups
- ✘ Letters
- ✘ Flyers
- ✘ Radio, TV recruitment ads
- ✘ Newspaper recruitment ads
- ✘ Website recruitment ads
- ✘ Telephone script
- ✘ Email or listserv announcements
- ✘ Follow up to initial contact (e.g., email, script, letter)
- ✘ Other

B.1.2. Describe how subjects will be identified

Recruitment. In cooperation with our Community Advisory Board the local PI in Tanzania will conduct meetings within each ward to introduce the project and present our plans. Following the ward meetings, trained study staff in Tanzania will conduct a PLACE assessment within each ward to identify, map and characterize all of the camps. We will use the camp lists generated through the PLACE assessment to randomly select camps proportional to the number of camps in that ward for inclusion in this trial. Once we have selected the camps for the trial study interviewers will return to

them to complete baseline interviews with each camp member.

B.1.3. Select any of the following procedures solely conducted for screening, recruiting, or determining the eligibility of prospective human subjects. (Note: you should only collect the minimal information needed for these purposes.)

- ✘ Obtain information through oral or written communication with the prospective subject or legally authorized representative
  This includes online, telephone, or in-person screening questionnaires or interviews.
- ✘ Obtain already collected identifiable private information or records
  Examples include review of medical charts, data repositories, and administrative records.
- ✘ Reviewing/testing identifiable biospecimens by accessing stored biospecimens and related information
- ✘ None of the above

B.1.4. For any selections made, please describe the procedures. (Respond “N/A” if “None of the above” is selected.)

No Answer Provided

B.1.5. For any information collected for these purposes, please describe when and how you will destroy the data if the participant declines to participate or is not eligible. (Respond “N/A” if “None of the above” is selected.)

No Answer Provided

B.1.6. Describe how and where subjects will be recruited and address the likelihood that you will have access to the projected number of subjects identified in A.2.

Participants will be identified through the camp rosters that we select for participation in the trial. Each camp maintains a roster of membership. Study interviewers will use these membership rosters to recruit partipants. We conducted a 2-year intervention development grant within one of the wards where we will be working. Based on the results of this intervention development work, we feel confident that we will be able to recruit the number of particpants we aim to recruit within our timeframe.

B.1.7. Describe how you will protect the privacy of potential subjects during recruitment

Membership rosters will be used for recruitment. If any individual member of a selected camp does not want to participate, this decision will be kept confidential. Camp leaders will not be informed which members within the camp are and are not particpating. Non-particpating camp members will not be contacted to participate in either the microfinance or the heatlh promotion intervention.

B.1.8. Describe how subjects will be contacted, if not addressed above

This has been addressed above.

B.1.9. Describe who (by role) will do the recruiting

We will have trained study staff conduct the recruitment. The same staff who will be conducting the baseline surveys will be responsible for elibility screening and recruitment. These staff (not yet hired) will be employed through Muhimbili University of Health and Allied Sciences (MUHAS), and thus their work will fall under the ethical oversity of the MUHAS IRB>

B.1.10. Describe efforts to ensure equal access to participation among women and minorities

Camps are primarily men's organizations. In our intervention development work, we found some camps had female members, but they were in the miniority. This interventon is designed to influence the atitudes and behaviors of men, and as such we will only be recruiting mae camp members to participate.

B.2. Protected Health Information (PHI)

Protected Health Information (PHI) is any identifiable information about the subject's health that relates to their participation in this research and is obtained from sources other than the subject, such as medical records, health care providers, insurance plans, etc. more

B.2.1. Are you requesting a limited waiver of HIPAA authorization?
If you need to access Protected Health Information (PHI) to identify potential subjects who will then be contacted, you will need a limited waiver of HIPAA authorization (see SOP 1801, 2.3). This does not apply to situations where you will never contact subjects directly (e.g., retrospective chart review), in which case you should request a full waiver under section D.

No

B.2.2. Will you need ongoing access to PHI (e.g., medical records) to conduct the study, beyond the identification of potential subjects as addressed above? In this case you will need to obtain a signed HIPAA Authorization from each subject.

No

B.3. Subject Contact, Duration and Privacy

B.3.1. Number of contacts per subject (contacts includes in-person, telephone, email, mailings, etc.)

3

B.3.2. Duration of each contact. If multiple contacts, provide the range or average time for each contact.

At baseline participants will be screened for eligibility which is expected to last approximately 5- 10 minutes. If indivduals are eligible, tey will be consented which may last 30 minutes. One men have been consented then they will be interviewed which may take 1 hour, and their specmens (blood and urine) will be taken which may take about 30 minutes. The interview and the specimens collection will be repeated at the post intervention assessment, and is expected to last the same legth of time at that point.

B.3.3. Total duration of individual subject's participation, including follow up evaluation, if applicable

Participants will be enrolled for a period of 30 months.

B.3.4. Where are you studying subjects or obtaining their data?

No Answer Provided

B.3.5. Provide more information about the location(s) where research will be conducted (e.g., if UNC Medical Center is checked in #4 above and study visits will be conducted in the CTRC, enter “CTRC” here.)

All study contact will happen in the community where participants are living in Dar es Salaam, Tanzania. Assessments and intervention activities will happen in/around the camps where the men are members.

B.3.6. Describe procedures that will ensure privacy of the subjects in this study. Examples include the setting for interviews, phone conversations, or physical examinations; communication methods or mailed materials (e.g., mailings should not indicate disease status or focus of study on the envelope)

As we did in the intervention development grant that we conducted prior to this trial, we will identify private spaces in or near the camps where we recruit the men. These spaces will be used for all assessments. These spaces may include empty school classrooms, vacated businesses, and other rented rooms.

B.4. Incentives for participation

B.4.1. Are there incentives (monetary or non-monetary) for subjects to participate or are you reimbursing subjects for study-related costs (e.g., travel, parking, hotel accommodations or childcare)?

No

B.4.2. Are you collecting Social Security numbers or ITIN for payment and/or tax-related purposes?

No

B.5. Costs to be borne by subjects

B.5.1. Will there be any costs that subjects will incur related to participation in the study? Do not include costs for standard care for which patients would be billed if they were not in this study. Also do not include the time spent participating in the study.

No

Part C. Existing Data, Records, Specimens

C.1. Data Sources

C.1.1. What existing records, data or human biological specimens will you be using? (Indicate all that apply or select 'None of the above'):

- ✗ Medical records in any format.

  ALERT: You must check both boxes: 1) Medical records in any format and 2) Electronic medical record using Epic, or you/your study team will not be granted access to Epic for research purposes.

- ✗ Electronic medical records using Epic, WebCIS or other electronic system
- ✗ Carolina Data Warehouse for Health (CDW-H) (for UNC and its affiliates only)
- ✗ Carolinas Collaborative Data Request and Review Committee (DRRC)
- ✗ Paper medical records

If you access the medical records of fewer than 50 patients under a full or limited waiver of HIPAA, submit a copy of your IRB approval letter and a completed Research Disclosure Form to Health Information Management (HIM). Do not submit this information to the IRB. For additional information about this process, you should contact HIM directly at : 919-595-5591 or 919-966-1225 or 919-595-5580.

- ✗ Data already collected from another research study

Were the investigators for the current application involved in the original collection?	--

- ✗ Patient specimens (tissues, blood, serum, surgical discards, etc.)

Has the clinical purpose for which they were collected been met before removal of any excess?	--

- ✗ Data already collected for administrative purposes
- ✗ Student records (You will need to satisfy FERPA requirements: see SOP 3101, section 3.1 for guidance)

✗ UNC Dental Records

✗ Data coming directly from a health plan, health care clearinghouse, or health care provider?

✗ Publicly available data

✗ Other

✗ None of the above

For EACH data source checked above, provide a description of the data, proposed use, how data were collected (including consent procedures), and where data currently reside.

None existing data will be accessed.

C.1.2. Describe your plans for obtaining permission from the custodians of the data, records or specimens (e.g., pathology dept, tissue bank, original researcher):

Not applicable.

C.1.3. Do the custodians of the data, records or specimens require a data use agreement?

No

C.2. Coding and Data Use Agreements

C.2.1. When you receive these data, records or human biological specimens will they be coded? Coded means identifying information that would enable the research team to readily ascertain the individual's identity has been replaced with a number, letter, symbol, or combination thereof (i.e., a code). If you will not be using existing materials, check "No."

No

Part D. The Consent Process

D.1. Obtaining informed consent from subjects

The standard consent process is for all subjects to sign a document containing all the elements of informed consent, as specified in the federal regulations. Some or all of the elements of consent, including signatures, may be altered or waived under certain circumstances. If you will be requesting a waiver answer "not applicable" for any of the following questions that will not pertain to this study. You will be asked to provide relevant information in the section below on waivers.

D.1.1. Will children under the age of majority in their locale (18 years in NC) be enrolled? (Note: Any minor subject who attains the age of majority during the course of the research study must provide consent as an adult, unless consent has been waived, which is requested in section D.3.1.)

Yes

Explain the process for obtaining consent from the subject, parental permission and/or minor assent as applicable (unless a waiver of permission will be requested later) in the sections below. The informed consent process should include the following:

- Provide the participant/parent/LAR with:
  - Information about the study in a language they understand
  - An opportunity to ask questions and have their questions answered
  - Adequate time to consider study participation
  - A signed copy of the consent form (a copy is acceptable)
- Avoid exculpatory language and undue influence.
- Document the consent process in the research record; if consent takes place on the same day as study procedures, document that informed consent was obtained prior to initiating any research-related procedures.

When explaining the process for obtaining consent/assent below, please incorporate the above information. (e.g., do not simply state that the participant will sign the form). The assent process should be developmentally appropriate and provide opportunities for children to discuss their willingness or unwillingness to participate. If assent is required, a child's dissent (unwillingness to participate) MUST be honored.

We are requesting a waiver of parental consent.

Check the characteristics of children to be enrolled: *

- ✗ 0 - 6 years
- ✗ 7 - 14 years
- ✓ 15 - 17 years

Explain the process for obtaining the assent of the child (unless waiver of assent will be requested, in which case you should provide justification here).

Waiver of parental consent will be requested.

D.1.2. Will adult subjects be enrolled in your study?

Yes

Explain the process for obtaining consent from the subject.

The informed consent procedures for this study have been designed to maximize understanding of potential risks. All consent forms will be translated into Kiswahili and back-translated into English to ensure correct use of language. Consent forms will be read aloud to participants by study interviewers. After reading the consent forms prior to seeing a signature, interviewers will ask participants to summarize the study and explain the reasons why they want to participate. At this point any misunderstandings regarding procedures, risk or benefits can be clarified. If there are cultural, literacy or political reasons why a signature is not appropriate, individuals will be allowed to mark the consent form with an 'X'.

D.1.3. Will decisionally-impaired subjects be enrolled in your study? (includes unconscious patients, some psychiatric disorders, others who lack the capacity to give consent)

No

D.1.4. Are you planning to obtain consent from any Non-English speaking subjects?

Yes

Click here to obtain the Translation Verification form, which should be completed and uploaded with Attachments at the end of the application.

If you will be obtaining consent in Spanish, consent form templates are provided on the OHRE website. If you will be obtaining consent in other languages, you will need to upload translations of the English consent form(s) once

approved by the IRB.

Describe how consent in the native language will be obtained. Address both written translation of the consent and the availability of oral interpretation. It is expected that the information in the consent document(s) will be communicated to participants or their legally authorized representative (LAR).

All consent forms will be translated into Kiswahili for use within this study. Study consent forms will be read aloud to all participants.

D.1.5. Describe who (by role) will be obtaining consent or parental permission.

Study interviewers will be trained to adminiser the consent form to all eligible participants prior to their enrollment.

D.1.6. Discuss the potential for influencing the subject's decision to participate. Describe steps that will be taken to minimize undue influence during the consent process. These might include a waiting period between the initial consent discussion and obtaining consent, or obtaining consent by someone other than a person with perceived authority (e.g., professor, employer, treating physician).

If men would like more time to decide to participate or not to participate in this trial, the staff who are responsible for enrollment and consent will offer them the opportunity to think about their decision, and scheule an appointment within the next 1-2 days to meet with them again and determine their interest in enrollment.

D.1.7. Has the sponsor of this study provided a model consent form?

No

D.2. Waiver of written documentation of informed consent

The default is for subjects to sign a written document that contains all the elements of informed consent. Under limited circumstances, the requirement for a signed consent form may be waived by the IRB. For example, this might occur for phone or internet surveys, when a signed consent form is either impractical or unnecessary, or in circumstances where a signed consent form creates a risk for the subject.

D.2.1. Are you requesting a waiver of any aspect of written (signed) documentation?

No

D.3. Full or partial waiver of consent

The default is for subjects to give informed consent. A waiver might be requested for research involving only existing data or human biological specimens. More rarely, it might be requested when the research design requires withholding some study details at the outset (e.g., behavioral research involving deception). In limited circumstances, parental permission may be waived. This section should also be completed for a waiver of HIPAA authorization if research involves Protected Health Information (PHI) subject to HIPAA regulation, such as patient records.

D.3.1. Are you requesting any of the following:

- ✗ a waiver of informed consent in its entirety
- ✓ a waiver or alteration of some of the elements of informed consent
- ✗ a waiver of HIPAA authorization (If you are accessing patient records for this research, you must also request a waiver of HIPAA authorization)

Describe which elements you wish to waive or alter

We are requesting a waiver of parental consent for the young men 15-17 whom we will enroll in this study. As we did in the intervention development grant (IRB #08-0502) that provides the preliminary evidence for this trial, we will request a waiver of parental consent to include men

15-17 years on the basis that under 45 CFR § 46.408 (c), an IRB has the authority to waive parental permission if it determines that “a research protocol is designed for conditions or a subject population for which parental or guardian permission is not a reasonable requirement to protect the subjects” and “an appropriate mechanism for protecting the children who will participate as research subjects is substituted” and “that the waiver is not inconsistent with Federal, State, or local law.” This population has an evolving capacity for self-determination that is proportional to the level of risk inherent in this study. This population of youth and the conditions in which they live and in which we would access them make obtaining parental permission for their participation not practical. Adequate protection has been substituted in the anonymity of the interviews and the referrals for services if needed.

To justify a waiver of the requirement for informed consent, you must affirm, by checking each of the following items that apply to this study. Provide an explanation.

Explain how the research involves no greater than minimal risk to subjects or to their privacy

The risks that particpiants may face do not constitute more than minimal risks.

Explain how the waiver will not adversely affect the rights and welfare of subjects (Consider the right of privacy and possible risk of breach of confidentiality in light of the information you wish to gather.)

The young men (15-17 years) whom we will enroll in this trial are enrolled members of the camps. They have made the decision to enroll and partiicpate in camp activities. As such they demonstrate an evolving capacity for self-determination.

Please explain why it would not be possible to conduct the study with only de-identified data (i.e. without any identifiers listed in A.9.)

--

Explain how the requirement to obtain consent would make the research impracticable, e.g., most of the subjects are lost to follow-up or are deceased.

It woul be impractiable to obtain parental consent for each camp member who is 15-17 years. When we conducted the intervention development grant that preceded this trial, we conducted a series of meetings in the community with leaders and parents of youth who wree members of camps. The parents of the camp members were aware and supportive of their participation in camp activities, and in he activities that we conducted as part of our intervention development work. We will work wtih our community advisory board to inform the community (including parents of youth in camps) of our activities within the camps.

D.3.2. If your request for a waiver applies to some but not all of your subject groups and/or consent forms, please describe and justify

We are requesting a waiver of parental consent for the men who are 15-17 years in the camps.

D.3.3. Does this request for waiver support a study design that involves deception or withholding of information?

No

Consent Forms

This submission requires the following consent forms

Template Type
Adult Consent Form
Assent Form Ages 15-17<br>**I am not using this template because:** Full waiver of consent requested in application (Section D.3.)

Parental Permission Form
I am not using this template because: Full waiver of consent requested in application (Section D.3.)

This submission includes the following consent forms

File Name	Document Type
Adult Consent Form.docx	Adult Consent Form

view consent forms

Attachments

This submission requires the following attachments

Document Type
Grant Application
International Research Guidance and Worksheet
Interview Questionnaire Survey
Translation Verification

This submission includes the following attachments

File Name	Document Type
Maman_R01_ApplicationPackage_12-2011.pdf	Grant Application
Swahili Recruitment_Script_to_be_used_with_Camp_Leaders.doc	Script for In-person Recruitment
Recruitment Script to be used with Camp Leaders.docx	Other Materials for Recruitment
Translation Verification Form Signed.pdf	Translation Verification
GILS_baseline_survey_10 11 2010_kisw1_corrected_skips_final.docx	Interview Questionnaire Survey
Interview Guide_Community Informant.doc	Interview Questionnaire Survey
Microfinance and Health Baseline Survey.docx	Interview Questionnaire Survey
Venue Verification Form_Yamanis.docx	Interview Questionnaire Survey
Venue and Event Report for Community Informants.doc	Interview Questionnaire Survey
CLEARENCE FORM.pdf	External IRB Approval Letter
AA_Mens_Brochure.pdf	Other
FORM_A_B_English_and_Swahili_together_7 2 2011.doc	Other
Swahili 2 Tanzania_MFI_and_Health_Consent_Form_Aug0912.doc	Other

view attachments

Addenda

Data Security Requirements

view addenda

If Principal Investigator of this study is a Student or Trainee Investigator, the Faculty Advisor certifies the following:

I accept ultimate responsibility for ensuring that this study complies with all the obligations listed above for the PI.

By certifying below, the Principal Investigator affirms the following:

I will personally conduct or supervise this research study. I will ensure that this study is performed in compliance with all applicable laws, regulations and University policies regarding human subjects research. I will obtain IRB approval before making any changes or additions to the project. I will notify the IRB of any other changes in the information provided in this application. I will provide progress reports to the IRB at least annually, or as requested. I will report promptly to the IRB all unanticipated problems or serious adverse events involving risk to human subjects. I will follow the IRB approved consent process for all subjects. I will ensure that all collaborators, students and employees assisting in this research study are informed about these obligations. All information given in this form is accurate and complete.

This study proposes research that has been determined to include Security Level 3 data security requirements. I agree to accept responsibility for managing these risks appropriately in consultation with departmental and/or campus security personnel. The Data Security Requirements addendum can be reviewed here.

Certifying Signatures:

Signature: Electronic Signature Received **Date:** 5/18/2012 07:32:09 AM

Suzanne Maman

The expectation is that this approval is being given on behalf of the head of the Department, Division, or Center. If the chair or director is an investigator on this project or otherwise conflicted in approving it, the Vice-Chair or Chair's designee should review it. By approving, you are certifying the following on behalf of your department, division or center:

- This research is appropriate for this Investigator and our department
- The investigator(s) are qualified to conduct the research
- There are adequate resources (including financial, support and facilities) available
- For units that have a local review committee for pre-IRB review, this requirement has been satisfied
- I support this application, and hereby submit it for further review

This study proposes research that has been determined to include Security Level 3 data security requirements. I agree to accept responsibility for managing these risks appropriately in consultation with departmental and/or campus security personnel. The Data Security Requirements addendum can be reviewed here.

If you are approving for other purposes (e.g., CTRC, DSMB, IBC, PRC, RSC, or other review committees), you affirm the following:

- The proposed submission is approved and may be forwarded for IRB review.

This study proposes research that has been determined to include Security Level 3 data security requirements. I agree to accept responsibility for managing these risks appropriately in consultation with departmental and/or campus security personnel. The Data Security Requirements addendum can be reviewed here.

Department Approval Signatures:

By signing in the appropriate space, the Department Chairperson(s) is indicating only that he/she has seen and reviewed this submission

Department: Health Behavior Operations

Signature: Electronic Signature Received **Date:** 5/21/2012 02:55:54 PM

Name & Title: Cheryl Gerringer, Business Officer
