# Supplementary material for: Results from a cluster-randomized trial to evaluate a microfinance and peer health leadership intervention to prevent HIV and intimate partner violence among social networks of Tanzanian men
Source: PLoS One. 2020 Mar 20;15(3):e0230371. doi: 10.1371/journal.pone.0230371 (PMC7083321; doi:10.1371/journal.pone.0230371)
Supplement: S1 Protocol — (DOCX) [file pone.0230371.s005.docx]

A Multilevel Intervention to Reduce HIV Risk among Networks of Men in Tanzania

Sponsored by:

The National Institute of Mental Health

University of North Carolina – Chapel Hill

331 Rosenau, 135 Dauer Drive, Campus Box 7440

Chapel Hill, NC 27599-7440

(Tel) +1- 919-966-3901 / (Fax) +1-919-966-2921

Version 1.0

March 20, 2014

Contents

[KEY ROLES AND CONTACT INFORMATION 5](#_Toc476835735)

[*Sponsoring Agencies:* 5](#_Toc476835736)

[*Operational Coordinating Center*: 5](#_Toc476835737)

[*Intervention Coordinating Center:* 5](#_Toc476835738)

[NIMH Microfinance and Health Intervention Study Group 6](#_Toc476835739)

[LIST OF ABBREVIATIONS AND ACRONYMS 6](#_Toc476835740)

[PROTOCOL SUMMARY 7](#_Toc476835741)

[1. INTRODUCTION 8](#_Toc476835742)

[1.1 Overview 8](#_Toc476835743)

[1.2 Significance 9](#_Toc476835744)

[1.3 Innovation 10](#_Toc476835745)

[1.4 Theoretical Underpinnings 11](#_Toc476835746)

[1.4.1 Theory relating to microfinance 11](#_Toc476835747)

[1.4.2 Theory relating to health leadership 12](#_Toc476835748)

[1.5 Rationale for Microfinance Intervention Component 12](#_Toc476835749)

[1.6 Rationale for Health Leadership Intervention Component 13](#_Toc476835750)

[1.7 Rationale for Working with Camps 14](#_Toc476835751)

[2. STUDY OBJECTIVES 15](#_Toc476835752)

[2.1 Primary Aim 1 15](#_Toc476835753)

[2.2 Primary Aim 2 15](#_Toc476835754)

[2.3 Secondary Aim 15](#_Toc476835755)

[3. STUDY DESIGN 15](#_Toc476835756)

[3.1 STUDY SCHEDULE 15](#_Toc476835757)

[3.2 Trial Design 16](#_Toc476835758)

[3.2.1 Overview 16](#_Toc476835759)

[3.2.2 First Primary Aim 16](#_Toc476835760)

[3.2.3 Second Primary Aim 17](#_Toc476835761)

[3.3 Study Duration and Endpoints 17](#_Toc476835762)

[4. STUDY ENROLLMENT 18](#_Toc476835763)

[4.1 Camp Enrollment 18](#_Toc476835764)

[4.1.1 Definition of Camps 18](#_Toc476835765)

[4.1.2 Camp Recruitment and Screening 18](#_Toc476835766)

[4.1.3 Camp Eligibility Criteria 18](#_Toc476835767)

[4.1.4 Camp Random Selection 19](#_Toc476835768)

[4.1.5 Replacing Ineligible Camps 19](#_Toc476835769)

[4.2 Camp Treatment Assignment Procedures 19](#_Toc476835770)

[4.3 Participant Screening and Enrollment 20](#_Toc476835771)

[4.3.1 Participant Eligibility Criteria 20](#_Toc476835772)

[4.3.2 Participant Exclusion Criteria 20](#_Toc476835773)

[4.4 Participant Retention 20](#_Toc476835774)

[4.5 Participant Withdrawal 21](#_Toc476835775)

[5. INTERVENTION COMPONENTS 21](#_Toc476835776)

[5.1 Overview 21](#_Toc476835777)

[5.2 Microfinance Implementation 22](#_Toc476835778)

[5.2.1 Business skill training 22](#_Toc476835779)

[5.2.2 Loan Centers and Groups 23](#_Toc476835780)

[5.2.3 Loan Financing 23](#_Toc476835781)

[5.2.4 Loan Application 23](#_Toc476835782)

[5.2.5 Weekly Loan Repayment Sessions 24](#_Toc476835783)

[5.2.6 Ongoing Access to Credit 24](#_Toc476835784)

[5.3 Camp Health Leadership Implementation 25](#_Toc476835785)

[5.3.1 Nomination Process 25](#_Toc476835786)

[5.3.2 Training 25](#_Toc476835787)

[5.3.3 Implementation of Strategies with Camps 25](#_Toc476835788)

[5.3.4 Booster Training Sessions 25](#_Toc476835789)

[5.4 Control Condition 25](#_Toc476835790)

[6. STUDY PROCEDURES AND ADVERSE EVENTS 26](#_Toc476835791)

[6.1 Overview 26](#_Toc476835792)

[6.2 Pre-test Survey 26](#_Toc476835793)

[6.3 Baseline Behavioral Survey 26](#_Toc476835794)

[6.4 Baseline STI Assessment 26](#_Toc476835795)

[6.5 Follow-up Behavioral Assessments 26](#_Toc476835796)

[6.6 Follow-up STI Assessment 26](#_Toc476835797)

[6.7 Qualitative Assessment 26](#_Toc476835798)

[6.8 Process Evaluation 27](#_Toc476835799)

[6.8.1 Intervention Training 27](#_Toc476835800)

[6.8.2 Microfinance Repayment Monitoring 27](#_Toc476835801)

[6.8.3 Camp Health Leaders Monitoring 27](#_Toc476835802)

[6.9 Language of Interviews 27](#_Toc476835803)

[6.10 Quality Assurance 27](#_Toc476835804)

[6.11 Adverse Events and Social Harms 28](#_Toc476835805)

[6.12 Security and Confidentiality 29](#_Toc476835806)

[7. EVALUATION OF OUTCOMES 29](#_Toc476835807)

[7.1 Overview 29](#_Toc476835808)

[7.2 Primary Endpoint - STI Prevalence 29](#_Toc476835809)

[7.2.1 Training, Quality Assurance, and Project Expertise for Lab 30](#_Toc476835810)

[7.2.2 Specimen Collection 30](#_Toc476835811)

[7.2.3 Specimen Handling, Storage, and Shipping 30](#_Toc476835812)

[7.3 Primary Endpoint - Gender-based Violence Perpetration 30](#_Toc476835813)

[7.4 Secondary Outcomes - Behavioral Outcomes 31](#_Toc476835814)

[7.4.1 HIV risk behaviors 31](#_Toc476835815)

[7.5 Mediating Variables/Outcomes 33](#_Toc476835816)

[7.6 Process Outcomes 36](#_Toc476835817)

[7.6.1 Microfinance Process Outcomes 36](#_Toc476835818)

[7.6.2 Microfinance Monitoring 36](#_Toc476835819)

[7.6.3 Camp Health Leadership Process Outcomes 37](#_Toc476835820)

[7.6.4 Camp Health Leaders Monitoring 37](#_Toc476835821)

[7.7 Qual*i*tative Outcomes 38](#_Toc476835822)

[8. DATA COLLECTION AND ADVERSE EVENT REPORTING 38](#_Toc476835823)

[8.1 Data Management Center 38](#_Toc476835824)

[8.2 Quantitative Data Management Plan 38](#_Toc476835825)

[8.2.1 Data Security 38](#_Toc476835826)

[8.3 Qualitative Data Management Plan 41](#_Toc476835827)

[9. STATISTICAL CONSIDERATIONS 41](#_Toc476835828)

[9.1 Quantitative Statistical Analysis Plan 41](#_Toc476835829)

[9.1.1 Sample Size, Power Calculations and Effect Size 41](#_Toc476835830)

[9.1.2 Aim 1 Sampling and Analytical Technique 41](#_Toc476835831)

[9.1.3 Aim 2 Sampling and Analytical Technique 42](#_Toc476835832)

[9.1.4 Mediation Sampling and Analytical Technique 42](#_Toc476835833)

[9.1.5 Treatment of Missing Data 42](#_Toc476835834)

[9.2 Qualitative Analysis Plan 43](#_Toc476835835)

[10. ETHICS AND PROTECTION OF HUMAN SUBJECTS 44](#_Toc476835836)

[10.1 Institutional Review 44](#_Toc476835837)

[10.2 Informed Consent 44](#_Toc476835838)

[10.3 Confidentiality 45](#_Toc476835839)

[10.3.1 Local Protections 45](#_Toc476835840)

[10.3.2 Statistical and Data Management Protections 46](#_Toc476835841)

[10.4 Benefits 46](#_Toc476835842)

[10.4.2 Community Benefits 46](#_Toc476835843)

[10.4.3 Benefits to Humanity 47](#_Toc476835844)

[10.5 Risks 47](#_Toc476835845)

[10.5.1 Individual Psychosocial Risks 47](#_Toc476835846)

[10.5.2 Individual Physical/Health Risks 47](#_Toc476835847)

[10.5.3 Community Risks 48](#_Toc476835848)

[10.6 Adverse Event Reporting 48](#_Toc476835849)

[10.7 Study Withdrawal and Discontinuation 48](#_Toc476835850)

[10.8 Incentives for Participation 48](#_Toc476835851)

[10.9 Linkages to Care 49](#_Toc476835852)

[10.10 Inclusion of Women 49](#_Toc476835853)

[10.11 Inclusion of Minorities 49](#_Toc476835854)

[10.12 Inclusion of Children 49](#_Toc476835855)

[11. LABORATORY SPECIMENS AND BIOHAZARD CONTAINMENT 50](#_Toc476835856)

[11.1 HIV Testing 50](#_Toc476835857)

[12. ADMINISTRATIVE PROCEDURES 51](#_Toc476835858)

[12.1 Community Preparedness and Involvement 51](#_Toc476835859)

[12.2 Study Coordination 51](#_Toc476835860)

[12.3 Statistical and Data Management Coordinating Centers 52](#_Toc476835861)

[12.4 Study Site Monitoring 52](#_Toc476835862)

[12.4.1 Intervention 52](#_Toc476835863)

[12.4.2 Data Management 52](#_Toc476835864)

[12.4.3 Documentation 52](#_Toc476835865)

[12.5 Protocol Compliance 52](#_Toc476835866)

[12.6 Minimizing Risks to Staff 52](#_Toc476835867)

[12.7 Investigator Records 53](#_Toc476835868)

[12.8 Policy on Data Sharing 53](#_Toc476835869)

[12.9 Dissemination of Study Results 53](#_Toc476835870)

# KEY ROLES AND CONTACT INFORMATION

# *Sponsoring Agencies:*

National Institutes of Health: National Institute of Mental Health

Institutions

University of North Carolina – Chapel Hill

Gillings School of Global Public Health

331 Rosenau, 135 Dauer Drive, Campus Box 7440, Chapel Hill, NC 27599-7440

*Suzanne Maman, PhD, Principal Investigator*

*Phone: (919) 966-3901*

*Email: maman@email.unc.edu*

Muhimbili University of Health and Allied Sciences

United Nations Rd, Dar es Salaam, Tanzania

*Lusajo Kajula-Maonga, MPhil, Principal Investigator*

*Phone: +255652874465*

*Email:* *sajokm@gmail.com*

American University

School of International Service

4400 Massachusetts Avenue, NW, Washington, DC 20016

*Nina Yamanis, PhD, Investigator*

*Phone: (202) 885-6562*

*Email:Yamanis@american.edu*

# *Operational Coordinating Center*:

University of North Carolina – Chapel Hill (*Suzanne Maman, PhD, Principal Investigator*)

Gillings School of Global Public Health

331 Rosenau, 135 Dauer Drive, Campus Box 7440, Chapel Hill, NC 27599-7440

# *Intervention Coordinating Center:*

Muhimbili University of Health and Allied Sciences

United Nations Rd, Dar es Salaam, Tanzania

***Statistical and Data Management Coordinating Center:***

University of North Carolina – Chapel Hill (*Suzanne Maman, PhD, Principal Investigator*)

Gillings School of Global Public Health

331 Rosenau, 135 Dauer Drive, Campus Box 7440, Chapel Hill, NC 27599-7440

***Research Site:***

**Dar es Salaam, Tanzania** *(Lusajo Kajula-Maonga, MPhil, Principal Investigator)*

Muhimbili University of Health and Allied Sciences

United Nations Rd, Dar es Salaam, Tanzania

# NIMH Microfinance and Health Intervention Study Group

Suzanne Maman, PhD^1^ *Principal Investigator*

Lusajo Kajula-Maonga MPhil^2^ *Principal Investigator*

Nina Yamanis, PhD*^3^*

J. Michael Boling, PhD^1^

Luz McNaughton Reyes, PhD^1^

Marcia Hobbs, PhD^1^

Sheila Leatherman, PhD^3^

Marta Mulawa, MHS^2^

Mrema Kilonzo, MS^2^

Peter Balvanz, MPH^1^

Stephane DeLong, MPH^1^

Donaldson Conserve, PhD^1^

Lauren Hill, MPH^1^

---------------------------------------------------------------------------------------------------------------------

*^1^ University of North Carolina at Chapel Hill*

*^2^ Muhimbili University of Health and Allied Sciences*

*^3^ American University*

This project is funded by the National Institute of Mental Health (NIMH) and provided to The Gillings School of Global Public Health at The University of North Carolina – Chapel Hill. The Muhumbili University of Health and Allied Sciences in Dar es Salaam, Tanzania and American University in Washington, D.C. will serve on the subcontract. The host country institution is Muhimbili University of Health and Allied Sciences

Each institution collaborating on the study holds a Federalwide Assurance, listed below.

*Institution: Assurance #:*

American University FWA00002262

Muhimbili University of Health and Allied Sciences FWA00004301

National Institute of Mental Health M1000

University of North Carolina at Chapel Hill FWA00004801

# LIST OF ABBREVIATIONS AND ACRONYMS

AIDS Acquired immunodeficiency syndrome

AU American University

CAB Community Advisory Board

CAPI Computer Assisted Personal Interviewing

CDC Center for Disease Control

CFR Code of Federal Regulations

CHL Camp Health Leader

CI Confidence interval

CT Chlamydia trachomatis

GBV Gender Based Violence

GCP Good Clinical Practice

GEMS Gender Equitable Men’s Scale

GPS Global Positioning System

HIV Human immunodeficiency virus

HSCL Hopkins Symptoms Checklist

ICC Interclass Correlation

ICT Immunochromatographic Test

IDI In Depth Interview

IMAGE Intervention for Microfinance and Gender Equity

IPV Intimate Partner Violence

IRB Institutional Review Board

LOT-R Life Orientation Test - Revised

MUHAS Muhimbili University of Health and Allied Sciences

NAAT Nucleic Acid Amplification Test

NIH National Institutes of Health (U.S.)

NIMH National Institute of Mental Health (U.S.)

NIMR National Institute of Medical Research (Tanzania)

NG Neisseria gonorrhea

OR Odds Ratio

OSHA Occupational Safety and Health Administration (U.S.)

PCR Polymerase Chain Reaction

PEP Post-exposure prophylaxis

PLACE Priorities for Local AIDS Control Efforts

PI Principal Investigator

QA/QC Quality assurance/quality control

SOP Standard operating procedure(s)

STI Sexually transmitted infection

TNPS Tanzania National Panel Survey

TSH Tanzanian Shillings

TV Trichomonas vaginalis

UNC University of North Carolina at Chapel Hill

UNAIDS Joint United Nations Programme on HIV/AIDS

USAID United States Agency for International Development

WHO World Health Organization

YOSEFO Youth Self-Employment Foundation

# PROTOCOL SUMMARY

**Purpose:** The purpose of this study is to determine whether men in camps randomized to receive a microfinance and health leadership intervention have a lower incidence of sexually transmitted infections (NG, TV, CT) and report perpetrating less physical or sexual violence against female sexual partners as compared to men in camps not randomized to receive the intervention.

**Design:** Cluster-randomized trial.

**Study Population:** Young men ages 15 or older that are members of camps (social groups) in Dar es Salaam, Tanzania.

**Study Size:** 1249 male camp members

**Intervention:** 60 eligible camps will be randomized in to either a microfinance and health leadership intervention or a control condition. Eligible members in the intervention camps will be able to participate in intervention activities.

**Duration and Follow-up:** This a 5 year cluster-randomized study. Behavioral data will be collected at baseline, 12 months, and 30 months after intervention. Biological data will be collected at baseline and 30 months.

**Primary Objectives:** To determine whether men in camps randomized to receive a microfinance and health leadership intervention have a lower prevalence of sexually transmitted infections (NG, TV, CT) and report perpetrating less physical or sexual violence against female sexual partners as compared to men in camps not randomized to receive the intervention.

**Primary Endpoints:** Incidence of STIs (NG, TV, CT) and perpetration of sexual violence against female sexual partners.

**Secondary Endpoints:** HIV risk and protective behaviors including: gender equity, alcohol and drug use, sexual relations and risk behavior, uptake of HIV counseling and testing; unprotected sexual intercourse; consistent condom use; delay of sexual debut; number of sexual partners, and sexual concurrency.

**Mediators**: In the event that program effects are identified, the degree to which the following mediate the multi-level intervention on STI and gender-based violence: hope, future orientation, social cohesion, collective efficacy, social support, emotional distress, sexual networks, social support and social influence.

**Qualitative:** Qualitative interviews with study participants will add contextual data for understanding various aspects of participant beliefs, attitudes, and behaviors towards endpoints measured.

# 1. INTRODUCTION

## 1.1 Overview

Finding effective strategies to reach out to young men and mobilize them to reduce their HIV risk is critical, given men’s control over the terms and conditions of most sexual partnerships. Unequal power distribution in relationships has a devastating impact on women, leading to HIV prevalence among young women in some sub-Saharan African countries four to seven times higher than among young men the same age.([AIDS, 2006](#_ENREF_2); [Initiative](#_ENREF_32)) Gender power differentials have negative consequences for men as well, leading to increased risk of physical and mental health problems, substance use, and low uptake of health-related services.([J. Pulerwitz & G. Barker, 2008](#_ENREF_70)) We need innovative approaches to address the structural and social determinants of young men’s risk. Lack of economic opportunity is a key structural determinant of risk that has negative consequences for men and has been linked to poor health outcomes.([Dodd, Munck, Organization, Bank, & Poor, 2002](#_ENREF_19); [Narayan-Parker, Patel, & Bank, 2000](#_ENREF_60)) The influence of social network members is a social determinant of men’s risk for both HIV and gender-based violence that can be addressed through interventions designed to change network norms.([Helleringer & Kohler, 2005](#_ENREF_30); [Latkin, Forman, Knowlton, & Sherman, 2003](#_ENREF_46))

For the past 12 years our group has conducted research in Dar es Salaam, Tanzania on HIV and gender-based violence. With support from NIMH we identified networks of young men who socialize in what are called “camps” and we successfully piloted a microfinance and health leadership intervention with men in camps like the one proposed in this application (R21 MH080577). Camps are enduring social groups of mostly men that have elected leadership, paid membership fees, and physical space to meet. The equivalent of a camp in US culture may be a cross between a club and a gang. Camps appear to be an urban phenomenon in Tanzania and our group is the first to have published data describing them.([Yamanis, Maman, Mbwambo, Earp, & Kajula, 2010](#_ENREF_100)) Men in camps engage in HIV risk behavior and in gender-based violence that put them and their partners at risk for HIV. Research suggests that microfinance combined with health promotion can lead to improvement in health outcomes, including reductions in HIV risk and gender-based violence.([Leatherman & Dunford, 2010](#_ENREF_47); [Morduch & Haley, 2001](#_ENREF_59); [P. M. Pronyk et al., 2006](#_ENREF_67)) However, few, if any well designed evaluations of microfinance and health programs with young men have been reported. This cluster randomized controlled trial will assess the efficacy of a combined microfinance and health leadership intervention designed to reduce HIV risk and gender-based violence among young men who socialize in camps in Dar es Salaam, Tanzania.

## 1.2 Significance

**Young men are important targets for HIV prevention interventions; however we continue to lack knowledge of how to access and intervene with men at risk for HIV.** HIV/AIDS is eliminating generations of youth in sub-Saharan African settings such as Tanzania. Sixty-percent of new infections in Tanzania occur among 16-24 year olds.([UNFPA, 2001](#_ENREF_96)) An estimated 2.7% of females and 1.2% of males aged 15-24 years are HIV-infected in Tanzania and 6.3% of females and 4.0% of males in this age range are HIV-infected in Dar es Salaam.([TACAIDS & NBS OCGS, 2013](#_ENREF_93)) Dar es Salaam, the commercial capital of Tanzania, is one of the regions most heavily impacted by HIV/AIDS, with an average of 6.9% of its population infected. ([TACAIDS & NBS OCGS, 2013](#_ENREF_93)) The development of HIV prevention interventions for youth is a priority for sub-Saharan Africa.([Caldwell, 2000](#_ENREF_12); [Dowsett et al., 1998](#_ENREF_20)) Existing gender norms encourage men to engage in high risk sexual behaviors, condone violence against women, and grant men the power to control the terms and conditions of their sexual relationships with women.([Dunkle et al., 2006](#_ENREF_22); [Jewkes et al., 2006](#_ENREF_34); [Martin et al., 1999](#_ENREF_52); [Noar & Morokoff, 2002](#_ENREF_61); [Raj et al., 2006](#_ENREF_72)) Consequences of the power imbalances include increased violence and HIV risk, often co-occurring in the same relationships. The power imbalance characteristic of gender relations among men and women has many of its roots in adolescence.([Dunkle et al., 2004](#_ENREF_21); [Maman et al., 2002](#_ENREF_50)) For some years now, UNAIDS and other organizations have called for research to engage men in interventions to change norms and behaviors that promote HIV risk and violence.([UNAIDS, 2005](#_ENREF_95)) Yet we still lack well-designed strategies to identify high risk networks of men and evidence-based interventions to reduce risk in these networks. Our proposed research is an innovative approach to do just that.

**Lack of economic opportunities for men has destructive health effects for men and their partners.** In Dar es Salaam, a city of 4.3 million people, unemployment among the general population is estimated to be between 13 to 40 percent and potentially higher among youth, many of whom, if they work, work unskilled and short term jobs.([Perullo, 2005](#_ENREF_64)) Opportunities for professional development for men without a secondary or tertiary school degree are extremely limited. Unemployment has destructive physical and mental health effects on men;([Bartley & Ferrie, 2010](#_ENREF_8)) stress from men’s inability to meet their role expectations as economic providers may lead to violence between partners.([Krishnan et al., 2010](#_ENREF_45); [Sivaram, Latkin, Solomon, & Celentano, 2006](#_ENREF_86)) Similarly lack of economic opportunities can lead to feelings of hopelessness, distress and interpersonal conflict for men.([J. Pulerwitz & G. Barker, 2008](#_ENREF_70))

## 1.3 Innovation

Our proposed intervention aims to reduce HIV risk and perpetration of violence in a population of high risk young men and women using a combination of intervention approaches that address important structural and social determinants of risk. Our approach is innovative in the following three ways:

**1) Addressing structural and social determinants of men’s risk through a multi-level intervention:** We propose providing business training and small loans to male and female members of camps with the goal of giving them access to resources and a skill set that will enable them to build a successful business enterprise (structural factors). Substantial evidence exists that men’s economic marginalization has negative effects on their health and the health of their families. Unemployment is one of the most salient issues that young men in Tanzania face. The loss of hope and agency from lack of resources may be as significant as material poverty in motivating high risk behavior.([J. Kim, Pronyk, Barnett, & Watts, 2008](#_ENREF_43)) We believe that engaging men in successful business enterprises may help to shift their goal orientation and time horizon, encouraging them to focus on longer term goals in place of behaviors that may lead to immediate gratification but result in substantial health risks.([Robbins & Bryan, 2004](#_ENREF_73)) This structural level intervention will be complemented by health promotion training we have developed for leaders. The leadership training is designed to change norms and behaviors related to HIV and gender-based violence (social factors). The health leadership component of the intervention capitalizes on the close social networks within camps among men who share norms and behaviors that carry substantial health risks. We have had success in engaging the natural leaders within these networks as health promoters.

**2**) **Leveraging social networks within camps for microfinance and health leadership training:** A key challenge to engaging men at high risk for HIV in health promotion efforts has been to identify networks of young men who can be reached through programs. Interventions designed not only to change individual behavior but also to incorporate a focus on social networks and communities are likely to result in more sustained behavior change.([DiClemente, 2003](#_ENREF_17); [JEMMOTT & Jemmott, 2000](#_ENREF_33); [Kelly, 1999](#_ENREF_36)) We identified close networks of young men that engage collectively in health promoting and health risk behaviors. The social networks within camps are critical for the success of the microfinance and the health leadership training. Group accountability is a key feature of many microfinance programs and one of the reasons these programs succeed. Such programs require individuals to form small groups and remain accountable to these groups. All group members must approve the business plan of a group member to be eligible for a microfinance loan. The close networks that exist within the camps in Dar es Salaam can be leveraged to reinforce the group accountability of loans, as we demonstrated in our pilot research. In addition, the camp networks include peer leaders whom we have already shown can be trained and mobilized to engage their peers in discussions about condom use and reducing multiple and concurrent partnerships, substance use, and partner violence**.**

**3)** **Reaching young men in venues that are amendable to interventions**. One of the challenges of engaging men in HIV and gender based violence prevention has been to identify the venues in which they can be reached effectively through programs. In our preliminary studies we found that camps are enduring structures, with fixed meeting spaces and common leadership and membership requirements, critical attributes for the success of the proposed intervention trial.

## 1.4 Theoretical Underpinnings

### 1.4.1 Theory relating to microfinance

The goal of microfinance is to reach the poor, provide them with training and ongoing access to credit to lift them out of poverty. In the case of this project, while poverty alleviation is important, we believe that the pathway through which individuals achieve that goal will have a greater influence on their health behaviors. *Hope and future orientation.* One theoretically important pathway through which microfinance might influence young men’s health behaviors is by providing them with hope. Snyder has defined hope as, “goal directed thinking in which a person appraises his or her perceived capability to produce workable routes to goals… as well as the potential to initiate and sustain movement along the pathways.”([C. Snyder, Cheavens, & Sympson, 1997](#_ENREF_87)) As Snyder explains, the individual who is hopeful or who can envision a hopeful future is more likely to engage in positive behaviors. Hope is a pivotal concept linking individual behaviors to social and economic contexts. ([Bernays, Rhodes, & Barnett, 2007](#_ENREF_10)) People with hope for the future and plans for achieving future goals are less likely to engage in activities in the present that put them at risk or jeopardize their future. By contrast, those without hope are more likely to place a lower value on the future. Without future goals there is little reason to avoid actions that may cause future harm but do not do so in the present. *Collective Efficacy:* Collective efficacy is another important theoretical construct in the pathway between microfinance and healthy behaviors for young men. Collective efficacy is defined as “the capacity of residents to achieve social control over the environment and to engage in collective action for the common good.” ([R.J. Sampson, 2003](#_ENREF_76)) It is the extent to which groups of individuals feel they can solve mutual problems together. Our preliminary work indicates that the networks we have tapped into through the camps are cohesive and enduring. A core principle of microfinance is accountability to a group. While individuals receive loans, they must form groups of approximately 5 individuals. An individual loan application requires approval by all group members. Groups are to attend repayment sessions with loan officers at a recurring day and time each week. If groups mount successful microfinanced projects, theory suggests doing so will lead to greater collective group efficacy. In turn, greater collective efficacy will have a positive influence on health behavior, leading men to reduce their risk for HIV by giving them the sense that they have some control over their environment.

### 1.4.2 Theory relating to health leadership

Working within social networks to influence change through peers is an approach guided by Diffusion of Innovation Theory. Rogers posited that behavior change in a population can be initiated and then will “diffuse” to others if enough influential members within the population visibly endorse, support and adopt an behavior.([Rogers, 1995](#_ENREF_74)) This theory proposes that peers are the source of influence most likely to influence behavior.([Kelly, 2004](#_ENREF_37)) While behavior occurs within a larger socio-cultural context, it is the norms communicated and reinforced by peers that are most likely to strongly influence behavior. The influence of peers on risk behavior is pronounced during adolescence. Peers have consistently been shown to influence adolescent behavior related to smoking, substance use and sex.([Almodovar, Tomaka, Thompson, Mckinnon, & O'Rourke, 2006](#_ENREF_3); [D'Amico & McCarthy, 2006](#_ENREF_14); [Sieving, Eisenberg, Pettingell, & Skay, 2006](#_ENREF_84)) Working with leaders to adopt and promote messages that promote health has been an effective HIV prevention strategy in other settings.([Kelly, 2004](#_ENREF_37); [Kelly et al., 1997](#_ENREF_38); [Kelly et al., 1992](#_ENREF_40))Through a nomination process, the men will identify leaders within the networks who are respected and trusted (see Section 5.3.1 below). These leaders will be invited to participate in training workshops to improve their communication strategies and bolster their social influence around reduction of HIV risk behavior and violence.

## 1.5 Rationale for Microfinance Intervention Component

**Microfinance combined with health interventions reduces poverty and leads to significant improvement in health outcomes. Yet men have been overlooked in microfinance.** Microfinance, the provision of small loans targeting low income individuals to spur entrepreneurship, is an increasingly common strategy to promote economic growth.([Karlan & Zinman, 2011](#_ENREF_35)) Multiple studies document the effectiveness of microfinance and its impact on poverty.([Khandker, 2005](#_ENREF_41)) A growing literature suggests that beyond poverty alleviation, microfinance programs can lead to positive health outcomes.([Dowsett et al., 1998](#_ENREF_20); [Dunkle et al., 2006](#_ENREF_22); [Leatherman & Dunford, 2010](#_ENREF_47); [Morduch & Haley, 2001](#_ENREF_59); [P. M. Pronyk et al., 2006](#_ENREF_67)) The concept of ‘tie-ins’ that link credit with skills building and education has been around for a long time and with respect to HIV has received increasing support.([P. Pronyk et al., 2005](#_ENREF_66)) A survey of 22 microfinance institutions in 14 African countries noted that 43% provide some form of health information to clients.([P. Pronyk et al., 2005](#_ENREF_66)) The most striking example of positive health effects resulting from a combined microfinance and health intervention is from the Intervention for Microfinance and Gender Equity (IMAGE) study in South Africa. In this cluster-randomized trial, women in the intervention arm, as compared to those in the control arm, who received small loans and attended biweekly health sessions were 55% less likely to report domestic violence, 15% more likely to have communicated with their partner about HIV and 20% less likely to have experienced controlling behaviors from their partner in the past six months. ([P. M. Pronyk et al., 2006](#_ENREF_67)) IMAGE was in part successful because it enhanced collective efficacy among the social networks participating in the microfinance intervention.([J. C. Kim et al., 2007](#_ENREF_44); [P. M. Pronyk et al., 2008](#_ENREF_68)) The vast majority of microfinance programs around the world target women. Microfinance organizations are beginning to make more loans to men and research has demonstrated positive health effects of microfinance with men. ([Accion.](#_ENREF_1); [T. W. Bank, 1998](#_ENREF_6); [Fernald, Hamad, Karlan, Ozer, & Zinman, 2008](#_ENREF_24)) To date, however, no intervention trials to evaluate a combined microfinance and health intervention for young men have been mounted.

## 1.6 Rationale for Health Leadership Intervention Component

**Social networks can have a powerful influence on behavior change; however, finding venues to work with male networks is a challenge.** Interventions designed not only to change the behavior of individuals but also to work with social networks to promote risk reduction are likely to result in sustained behavior change.([DiClemente, 2003](#_ENREF_17); [JEMMOTT & Jemmott, 2000](#_ENREF_33); [Kelly, 1999](#_ENREF_36)) Social networks provide important environments for shared peer norms and social influence processes to occur naturally.([Amirkhanian et al., 2005](#_ENREF_4)) Adolescents, in particular, are influenced by the behaviors and norms of their peers.([Andrews, Tildesley, Hops, & Li, 2002](#_ENREF_5); [Li, Barrera, Hops, & Fisher, 2002](#_ENREF_49)) Several studies from Tanzania have described how youth communicate mostly with their same-gender peers about sex.([Dilger, 2003](#_ENREF_18); [Matasha et al., 1998](#_ENREF_54); [Setel, 1999](#_ENREF_82)) The relationships and interactions that occur within social networks are considered critical to understanding sexual behavior and HIV risk.([Bond, Valente, & Kendall, 1999](#_ENREF_11); [D. & Knowlton, 2005](#_ENREF_15); [Friedman & Aral, 2001](#_ENREF_26); [Youm & Laumann, 2002](#_ENREF_101)) Interventions that strive to alter social network norms to promote HIV protective behaviors have been proposed as a sustainable way to change behavior, since they tap into naturally existing social structures.([Latkin et al., 2003](#_ENREF_46)) Social network interventions that work with network leaders to promote behavior change have shown a 30% reduction in network members’ risk behaviors.([Kelly, 2004](#_ENREF_37); [Kelly et al., 1997](#_ENREF_38); [Kelly et al., 1991](#_ENREF_39); [Kelly et al., 1992](#_ENREF_40)) Networks often function in places that provide settings for social interaction. Shared meeting space is particularly important for youth, who seek the company of their peers but often have no space of their own to socially interact. Reaching youth at risk in youth-dominated venues is challenging because school-based interventions often miss youth most at risk, and youth in settings such as Dar es Salaam are often not in worksites because of high unemployment.([Stroeken et al., 2011](#_ENREF_92))

## 1.7 Rationale for Working with Camps

**Through an NIMH intervention development grant (R21 MH080577) our group identified viable locations and feasible strategies for intervening with high risk networks of men.** Using the PLACE methodology we identified and characterized 73 venues, locally named “camps,” where young men socialize within one ward of Dar es Salaam. While some health risk behaviors occur among camp members, camps are primarily constituted by entrepreneurial youth who use them as meeting spaces to provide one another with social support. Camps we surveyed had existed for an average of 8 years (range 4-13 years), had an average of 52 members between the ages of 15-40 years, and were formed by men who wanted to claim a space where they could engage in social activities such as soccer or playing pool. The 670 men in camps whom we interviewed reported substantial risk behaviors: 47% reported 2 or more sex partners in the past year; 47% of those who had ever had sex reported one or more concurrent sexual partnerships in the past six months; 21% had at least one symptom of an STI in the past 4 weeks; and 41% reported ever perpetrating physical or sexual violence against a female partner. The majority of men (73%) socialized in their camp daily for several hours, and over half (53%) were unemployed. Our qualitative research described how youth developed a democratic system for running the camps that minimally included a chairperson, a secretary and a treasurer.([Yamanis et al., 2010](#_ENREF_100)) Members paid dues to belong, and all camps maintained written membership records. In the second year of the grant, which was recently completed in August, 2011, we partnered with a microfinance organization to pilot a microfinance and health leadership intervention with men in three camps. Nineteen men received a two-week business training course, received loans of $100 each, participated in groups to guarantee loan repayment, and had weekly repayment sessions over 6 months. We also recruited 30 peer-nominated leaders from the three camps to participate in health leadership training that emphasized communication skills and strategies to influence peer behavior. Results of the pilot demonstrate the feasibility and acceptability of this multi-level approach. Only one of the 19 men who received a loan defaulted on his loan. We learned important strategies to motivate and support men through the repayment process, such as having more frequent repayment sessions, building ongoing business skill training into the regular repayment sessions, and providing more explicit instructions on record keeping. The twenty leaders who completed the initial two week training course on communication and social influence remained engaged in the pilot intervention for 9 months. Two additional booster training sessions, and maintenance of regular records of their communications with their peers about HIV and violence prevention were also part of the intervention.

# 2. STUDY OBJECTIVES

## 2.1 Primary Aim 1

Enumerate and characterize camps where young men at risk for HIV acquisition and transmission socialize in four wards of Dar es Salaam.

## 2.2 Primary Aim 2

To determine whether men in camps randomized to receive a microfinance and health leadership intervention have a lower prevalence of sexually transmitted infections (NG, TV, CT), and report perpetrating less physical or sexual violence against female sexual partners as compared to men in camps not randomized to receive the intervention.

## 2.3 Secondary Aim

To determine whether men in camps randomized to receive a microfinance and health leadership intervention report less risky and greater protective behaviors related to HIV acquisition including: gender equity, alcohol and drug use, sexual relations and risk behavior, uptake of HIV counseling and testing; unprotected sexual intercourse; consistent condom use; delay of sexual debut; number of sexual partners, and sexual concurrency.

In the event that program effects are identified, a secondary objective is to assess the degree to which the following mediate the impact of the multi-level intervention on STI and gender-based violence: Hope, Future Orientation, Social Cohesion, Social Support, Collective Efficacy, Emotional Distress, and Sexual Networks

# 3. STUDY DESIGN

## 3.1 STUDY SCHEDULE

This study, from planning through data analysis, is scheduled to last 5 years. The intervention component of the study will last 2 years. A timeline for the project is presented below.

| Activity | Year1 | | | | Year 2 | | | | Year 3 | | | | Year 4 | | | | Year 5 | | | |
| --- | --- | --- | --- | --- | --- | --- | --- | --- | --- | --- | --- | --- | --- | --- | --- | --- | --- | --- | --- | --- |
|  | 1 | 2 | 3 | 4 | 1 | 2 | 3 | 4 | 1 | 2 | 3 | 4 | 1 | 2 | 3 | 4 | 1 | 2 | 3 | 4 |
| Planning and obtaining approvals |  |  |  |  |  |  |  |  |  |  |  |  |  |  |  |  |  |  |  |  |
| Developing protocols |  |  |  |  |  |  |  |  |  |  |  |  |  |  |  |  |  |  |  |  |
| Staff recruitment and training |  |  |  |  |  |  |  |  |  |  |  |  |  |  |  |  |  |  |  |  |
| Identification and enumeration of camps |  |  |  |  |  |  |  |  |  |  |  |  |  |  |  |  |  |  |  |  |
| Screening and enrollment |  |  |  |  |  |  |  |  |  |  |  |  |  |  |  |  |  |  |  |  |
| Baseline assessment |  |  |  |  |  |  |  |  |  |  |  |  |  |  |  |  |  |  |  |  |
| Randomization |  |  |  |  |  |  |  |  |  |  |  |  |  |  |  |  |  |  |  |  |
| Intervention |  |  |  |  |  |  |  |  |  |  |  |  |  |  |  |  |  |  |  |  |
| Qualitative cohort interviews |  |  |  |  |  |  |  |  |  |  |  |  |  |  |  |  |  |  |  |  |
| Follow-up assessment |  |  |  |  |  |  |  |  |  |  |  |  |  |  |  |  |  |  |  |  |
| Data cleaning and analysis |  |  |  |  |  |  |  |  |  |  |  |  |  |  |  |  |  |  |  |  |
| Dissemination |  |  |  |  |  |  |  |  |  |  |  |  |  |  |  |  |  |  |  |  |

## 3.2 Trial Design

### 3.2.1 Overview

This is a cluster randomized controlled trial to assess the efficacy of an intervention designed to reduce HIV risk and gender-based violence among young men who socialize in camps in Dar es Salaam, Tanzania. Sixty camps in four economically disadvantaged wards within Dar es Salaam will be randomized to receive either two years of a combined microfinance and health leadership intervention or a control condition. We will conduct quantitative assessments at baseline, 12 months and 30 months, and three qualitative assessments through the life of the study.

### 3.2.2 First Primary Aim

To accomplish the first primary aim to enumerate and characterize camps within four wards in Dar es Salaam, a PLACE (Priorities for Local AIDS Control Efforts) assessment will be conducted. PLACE is venue-based sampling methodology that was developed to identify venues where individuals at high risk for HIV transmission and acquisition meet new sexual partners.([Weir et al., 2003](#_ENREF_97)) The PLACE assessment strategies to be used for this study include community informant and camp verification interviews.

#### 3.2.2.1. Community Informant Interviews

Brief, structured interviews will be conducted with community informants to identify the camps. The goal is to interview as many people as necessary within each ward until there is saturation in the venue lists. The PLACE method recommends interviewing 4 community informants per 1,000 people in catchment area to reach saturation of locations high in HIV risky behaviors. Since camps have already been decided to be the intervention locations, we have estimated that 2 community informants per 1,000 people will be sufficient to develop a saturated list of camps.

Prior to beginning interviews, study staff will inform local government officials as to the purpose and process of the study. Interviewers will then systematically proceed through each *mtaa* (similar to street) in all four wards to complete community informant interviews. Community members familiar with the area and at least 15 years old will be interviewed. Interviewers will approach people encountered in the *mtaa*, and if willing to be interviewed will record they type of community informant for each interview from a pre-developed list (i.e. street vendor, mechanic, security guard, student). Community Informants will then be asked to provide the names of all camps they know of in the area as well as directions to the camp. This phase of data collection is expected to be completed in 2 months.

#### 3.2.2.2 Camp Verification Interviews

Using the list of camps generated during the Community Informant interviews, interviewers will verify the geographical location of camps in the four wards of this study and record camp characteristics using the Camp Verification form. Following directions provided in the Community Informant interviews, research staff will record whether or not they found the camp, refine directions if found, record physical characteristics of the camp, and record GPS coordinates to map and view relative location of camps. The interviewer will then seek a camp leader for the Camp Verification interview, and if not available, other camp members. Camp leaders willing to participate and at least 15 years old will be asked other specific characteristics of the camp such as years of operation, number of members, whether dues are required, activities at the camp, time and day when most busy, and general safety among others. The camp verification component will generate a pool of camps for selection in to the trial. This phase of data collection is expected to be completed in 2 months.

### 3.2.3 Second Primary Aim

To achieve our second primary aim, to evaluate the efficacy of a combined microfinance and health leadership intervention we will conduct a trial among 1252 men in 60 camps that are randomly selected proportionally across four wards. Our primary hypothesis is that the combined microfinance and health leadership intervention will be associated with a lower incidence of sexually transmitted infections and other HIV risk behaviors and less perpetration of violence against female sexual partners over time among men in camps randomized to the intervention as compared to men in camps randomized to the control condition. We also hypothesize that men’s hope for, and orientation towards, the future, as well as the support, social influence and the collective efficacy of their networks, will mediate the effect of the intervention on these outcomes.

## 3.3 Study Duration and Endpoints

The intervention will be implemented for two years, and the total duration of the trial will be 5 years. **The primary endpoints** are sexually transmitted infections including Neisseria gonorrhea (NG), Trichomonas vaginalis (TV), Chlamydia trachomatis (CT), and perpetration of physical or sexual violence against a sexual partner. A random sample of 50% of participating men will be flagged for an STI test at baseline to assess relative prevalence, and all men will be tested at endline. A test of relative prevalence at baseline will aid in establishing equivalency in the two arms; any lack of equivalency then will be controlled for appropriately at endline. All participants will complete a behavioral assessment at baseline, 12 months and 30 months to assess secondary objectives. **Secondary endpoints** are HIV risk and protective behaviors including: gender equity, alcohol and drug use, sexual relations and risk behavior, uptake of HIV counseling and testing; unprotected sexual intercourse; consistent condom use; delay of sexual debut; number of sexual partners, and sexual concurrency. **Key mediators** are hope, future orientation, social cohesion, collective efficacy, emotional distress, sexual networks, social support and social influence.

# 4. STUDY ENROLLMENT

This study will take place in Kinondoni district, the most populous (over 1 million) and most impoverished of the three districts in Dar es Salaam, Tanzania.([Statistics, 2002](#_ENREF_90)) Four wards in Kinondoni characterized by high risk for HIV among young people have been selected for recruitment. These four wards include Manzese (population 66,543), Tandale (population 44,853), Mwananyamala (population 44,344), and Mabibo (population 77,639). In all four wards over a third of the population are males aged 15-29 (range among four wards is 36-38%).([Statistics, 2002](#_ENREF_90)) Illicit drug use, alcohol use, and commercial sex are common to the four wards. In one study of HIV infection among drug users, 66.7% of syringes from male injectors in Manzese and Tandale tested HIV positive and 40% of syringes from male injectors in Mwananyamala tested HIV positive.([McCurdy, Ross, Kilonzo, Leshabari, & Williams, 2006](#_ENREF_55)) Social venues called camps, where young men socialize, have been identified as locations of focus for this study.

## 4.1 Camp Enrollment

### 4.1.1 Definition of Camps

Camps are fixed venues where young men socialize in Dar es Salaam, Tanzania. Camps generally have a democratic system that minimally includes a chairperson, a secretary, and a treasurer. Members often pay dues to belong and maintain written membership records. Camps will serve as the unit of analysis for this trial.

### 4.1.2 Camp Recruitment and Screening

Camps will be identified through the PLACE-based community informant and camp verification interviews. Approximately 2 community informants per 1,000 person population in each of the four participating study wards will be recruited for interviews, or between 400 and 500 individuals. A list of potential camps in the four study wards, and directions to these camps will be developed through Community Informant interviews. All camps identified through Community Informant interviews will then be visited for Camp Verification interviews to confirm existence and current operation, record GPS coordinates, and record characteristics of the camp through interviews with camp leadership. Camps included in the sampling frame will be those confirmed to exist and be in operation, of which GPS coordinates were recorded, and for which at least half of the camp verification interview questions were completed.

### 4.1.3 Camp Eligibility Criteria

Camps must meet a number of requirements to be eligible for selection in to the study. Camps not meeting criteria will be eliminated from the sampling frame. Eligibility criteria include:

- Was not part of the pilot study
- Has 20 members or more
- Has less than 80 members
- Has been in existence at least one year
- Reported no violent incidents during which a weapon was used within the past 6 months
- Research Assistant did not feel unsafe during the verification interview

### 4.1.4 Camp Random Selection

60 camps will be randomly selected in to the study, and later assigned in to one of two conditions – intervention or control. Due to the density of camps within wards and the contiguous nature of some camps, measures will be taken to minimize contamination of potential intervention and control camps before random assignment. The following steps will be taken during the random selection of camps.

1. Local staff will group geographically contiguous camps posing a threat to potential contamination. Eligible camps will be mapped by GPS coordinates on a street layout of the four wards to facilitate grouping. Groups will contain up to 6 camps, individual isolated camps will not need to be put in a group.
2. Groups will be selected for inclusion in the study using probability proportionate to size. Thus, groups with numerous camps of large membership have the potential to be selected more than once.
3. Simple random selection of camp(s) will be completed among camps in each group selected in to the study. Camps from the same group randomly selected in to the study will necessarily receive the same condition during the condition assignment phase.

### 4.1.5 Replacing Ineligible Camps

Following random selection, camp rosters will be collected from leaders of all selected camps. Selected camps will be replaced if current rosters do not meet size eligibility requirements reported during camp verification, or camps refuse to participate. To replace ineligible or non-interested camps, another random selection using probability proportionate to size will be conducted from all eligible camp groups. The group selected in the same numeric order as the group with the camp to be replaced (e.g. 15^th^ group selected) will have a camp selected in to the study through simple random selection within the group. This process will be repeated until the sample contains 60 eligible and consenting camps.

## 4.2 Camp Treatment Assignment Procedures

Following random selection, camps will be randomly assigned to a condition 1 or 2, not yet linked to intervention or control. Following up on the grouping procedure used during random selection to minimize potential contamination, groups with single camps selected in the study will first be randomly assigned to condition 1 or 2. Groups with two camps selected in to the study will next be randomly assigned to condition 1 or 2, followed by groups with three camps.

Treatment assignment for the 60 randomly selected and eligible camps will be conducted during a CAB meeting. At the meeting an individual representing condition 1 or 2 will be presented with 5 balloons, each with a folded paper enclosed. In three of the balloons the paper will read “Try again,” one paper will contain the number 1 and one paper will contain the number 2. The individual will be asked to pop one balloon and read the enclosed paper. A “try again” message will prompt the individual to select and pop another balloon. The first number encountered upon popping will represent the group to receive the intervention condition. For example, if the number 2 was encountered first, all camps in Condition 2 are assigned to the intervention condition.

## 4.3 Participant Screening and Enrollment

All members of camps selected in to the trial are preliminarily eligible for inclusion in the study. Following random selection, camp leaders from camps selected in to the trial will be given a camp roster template to complete. The camp leader will be responsible for recording the name of every camp member as well as birthdate, gender, phone numbers, length of time as camp member, whether or not the member intends to stay in the city for the next 30 months, and contact numbers for 2 friends or family members. Participants not meeting eligibility criteria listed below will not be entered in to the study.

### 4.3.1 Participant Eligibility Criteria

Study participants will be male members of camps in Dar es Salaam, Tanzania. Camps are predominately male social groups where members meet in fixed locations. Eligibility criteria for camp members to be able to participate in the study include:

- Participant is at least 15 years old
- Has been member of camp for at least 3 months
- Visits camp at least once a week
- Intends to live in Dar es Salaam for next 30 months
- Willing to give contact information of self and relative or friend

### 4.3.2 Participant Exclusion Criteria

Individuals will be excluded from the baseline behavioral assessment if they meet any of the following criteria:

- Does not meet all inclusion criteria
- Unable to participate due to psychological disturbance, cognitive impairment or threatening behavior

## 4.4 Participant Retention

At enrollment into this study, staff will record locator information (name, telephone number, typical place of residence or sleeping location) and names of contacts who usually know where to find the participant, with an assurance not to reveal the participant’s involvement in the study.

The following systems are in place at the site for cohort retention:

- **Participant scheduling:** All scheduled appointments and missed appointments will be tracked on both physical contact forms as well as entered in to Computer-Assisted Personal Interviewing (CAPI) tablets.
- **Participant Follow-up:** Study participants who miss scheduled appointments will be contacted within 24 hours by telephone or by home visit if they did not leave a telephone contact.
  - If unsuccessful in contacting participants, we will follow up with contacts they provided of friends or relatives. If participants are not able or do not wish to attend a study visit, reasons for non-attendance will be recorded. Participants are considered lost to follow-up if unable to be contacted through 5 attempts.
    - **Travel Expense Compensation:** Participants will receive monetary compensation of TSH 10,000 for travel expenses to the site where interviews take place.
- **Camp Roster Updates:** After each wave of data collection participant contact information will be updated on physical files.
- **Community Engagement:** Community engagement through CAB meetings will facilitate community support needed to track and follow up with participants. CAB meetings will occur two times per year through the life of the study.
- **Interpersonal Relationship Building:** Appealing to participants interpersonally has been an effective strategy to retain participants. Our staff will have the opportunity to interact with participants during each wave of data collection, and meetings with the CAB and camp leaders.
- **Intervention Activities:** Intervention activities including microfinance repayment and camp health leader conversation documentation offer opportunities to check-in with members about the state of their camp and membership.

## 4.5 Participant Withdrawal

Participants may voluntarily withdraw from the study at any time. If a participant chooses to withdraw during an assessment period they can choose not to have their assessment responses submitted to the study team.

Participants also may be withdrawn if the principal investigator, study sponsor, government or regulatory authorities, or site institutional review boards (IRBs) terminate the study prior to its planned end date. In such cases the Principal Investigator will review the reasons for withdrawal with the IRB and Protocol Biostatistician prior to participant notification. The Principal Investigator may decide to include data collected prior to participant withdrawal in study analyses.

Every reasonable effort will be made to complete a final assessment of participants who leave the study prior to the planned termination date. Study staff will record the reason(s) for all withdrawals from the study in participants’ study records.

# 5. INTERVENTION COMPONENTS

## 5.1 Overview

This study employs a combined microfinance and health leadership behavioral intervention. Prior research has demonstrated that microfinance combined with health interventions reduces poverty and can lead to significant improvement in health outcomes, however these interventions have not been evaluated among young men. The microfinance component provides small loans and business skills training for participants to begin building viable businesses, encouraging increased income. The health leadership component uses nomination of public opinion leaders in camps and health leadership training to inform and encourage leaders to talk with others about HIV risk behaviors and GBV.

## 5.2 Microfinance Implementation

Youth Self-Employment Foundation (YOSEFO), a microfinance institution in Dar es Salaam, will implement the microfinance component of the intervention (www.yosefo.org). YOSEFO has been providing loans to young men and women in Tanzania since1996, and partnered with our team for a pilot of this trial. As of 2011, the total number of YOSEFO clients was 18,200, with an outstanding loan amount of 3.9 billion Tanzanian Shillings ($2.3million USD). The standard principles and practices in microfinance that successfully guided our pilot study and will guide implementation of this study include:

### 5.2.1 Business skill training

Camp members will receive one week training on entrepreneurship skills and loan financing facilitated by YOSEFO. The trainings will focus on how to generate viable business ideas, how to assess markets, how to start and scale up businesses, how to manage businesses, and loan term and repayment (Appendix A).

**5.2.1.1 Entrepreneurship**

Entrepreneurship training will take place during the first four days of the one week training. Lessons will include: Business environment; entrepreneurship and the entrepreneur; marketing, sales and customer service; techniques for business growth and expansion; costs, sales and prices in business; and effects of HIV/AIDS, business and family.

**5.2.1.2 Loan Financing**

Participants will learn the logistics of loan financing during the last day of training. Lessons will include: Obtaining business capital; loan collateral; loan criteria; loans and interest rates; loan groups and centers; loan application; loan repayment.

### 5.2.2 Loan Centers and Groups

To encourage accountability in loan repayment, camp members must form groups of 5 members to receive a loan. All groups in the same camp will form a center for the purpose of repayment. Each center meets weekly at a recurring time for repayment. Repayment of a loan is the responsibility of the borrower, but members are encouraged to follow up on repayment among all group members.

**5.2.2.1 Loan Group Formation**

Camp members will be responsible for forming their own group of five members. Groups are formed during the final day of training with one member being nominated as the group leader.

**5.2.2.2 Center Leadership**

Each center will nominate a chairman, secretary, and discipline master by vote. The chairman will be responsible for collection of weekly installments to present to a Loan Officer. The secretary is responsible for monitoring attendance at repayment sessions. The discipline master enforces any rules created by the center.

### 5.2.3 Loan Financing

All loans are subject to an interest rate and other fees. Participants have a choice of interest paid over a 6 month repayment period (18%) or 9 months (27%). Other fees and deposits include:

- Entry fee (One-time payment of TSH 3,000)
- Loan fee (1% of loan amount)
- Loan insurance (1.5% of loan amount)
- Life insurance (Annual payment of TSH 550)
- Savings (Weekly TSH 2,000 deposit)

(Appendix B)

### 5.2.4 Loan Application

Loan application occurs over three weeks and includes registration, application, and savings deposits of U.S. $5. This process is enacted to establish the habit of participants attending weekly meetings, and to minimize chances of participants borrowing from another institution to fund the savings required to receive a YOSEFO loan. In the first week individuals must register for loans. In the second week individuals complete a loan application form that includes details on the planned business, physical collateral, and contacts for group members. Through these two weeks and the following week leading up to loan disbursal, camp members must deposit the equivalent of 5% of the loan amount to savings before receiving the loan.

**5.2.4 Loan Approval**

Participants that complete loan registration, loan application, and deposited required savings will have their loans reviewed for approval. All group members must sign the loan application indicating they approve the business idea. Upon group approval of loan, the center chairman nominates another center member to visit the home of the loan applicant to ensure s/he has the physical collateral listed on the loan application. When these procedures are completed and confirmed, loans are approved for an initial loan of TSH 160,000 ($100 USD).

### 5.2.5 Weekly Loan Repayment Sessions

Participants will meet with a loan officer for mandatory weekly repayment sessions. At these sessions members are required to pay principal, interest, and provide a weekly savings deposit.

For a first loan of TSH 160,000, weekly repayments for the two loan terms are:

- 6 months: TSH 5,300 principal and interest + TSH 2,000 in savings = TSH 7,300 per week
- 9 months: TSH 7,300 principal and interest + TSH 2,000 in savings = TSH 9,300 per week

(Appendix B)

Repayment sessions will be led by a Loan Officer, and may contain occasional discussions or informational sessions on successful business skills. During each session group leaders will collect payments from all others in their group, and then present the collective money to the center leader. Repayments will be recorded by the Loan Officer, who will also transfer payments back to YOSEFO.

### 5.2.6 Ongoing Access to Credit

Ongoing access to incrementally larger loan amounts is a key feature of microfinance. After successfully completing a loan cycle, camp members will be eligible for successive loans at increasing amounts. Required minimum savings to receive the loan will also increase with successive loan. Amounts available for the first three loan cycles are:

- 1^st^ Loan: TSH 160,000 (5% savings required)
- 2^nd^ Loan: TSH 300,000 (12.5% savings required)
- 3^rd^ Loan: TSH 450,000 (25% savings required)

Funds from this project will cover the first three loan cycles for participating intervention camp members. Those seeking a fourth loan will be eligible to receive this and future loans from YOSEFO. Minimum deposited savings for loans 3 or greater will be 25% of loan amount. The sum of suggested weekly savings deposits (TSH 2,000) will be greater than the minimum deposit required, and can be applied to this requirement.

## 5.3 Camp Health Leadership Implementation

The second component of the intervention targets peer leaders within their camps as health promoters for behavior change. This intervention component builds on the fact that it is possible to identify peer nominated leaders within the camps who are respected and trusted by their peers. To implement this intervention component we will take the following steps:

### 5.3.1 Nomination Process

Twenty percent of the total number of members at each camp will be nominated in to a Health Leadership role (n = Camp members on roster x 0.20). Nomination will occur at each camp separately when at least 50% of camp roster members are present. After discussing attributes of leaders, each camp member in attendance will nominate confidentially up to 3 leaders in their camps with these qualities. Leaders identified will then be approached and asked if they are interested in being trained as peer leaders for their camp.

### 5.3.2 Training

Nominated camp health leaders will attend a one week training that focuses on building skills in effective communication and social influence. Health leaders will be trained in groups with a maximum of 40 individuals at one time. The same training manual used in the pilot intervention, with minor modification will be used in this trial. The training will provide camp leaders with knowledge and will address myths and misconceptions related to HIV transmission, condoms, violence, and multiple partnerships. (Appendix C)

### 5.3.3 Implementation of Strategies with Camps

Once trained, leaders will be asked to implement the communication and social influence strategies they learned within their camps. Leaders will record information about the number of times they engage in peer discussions about the issues discussed in training, the topics, the sex of their discussant, and any challenges encountered. See Process Evaluation in Section 7.8.

### 5.3.4 Booster Training Sessions

Follow-up training sessions for the leaders will occur at 6 month intervals after the intervention has launched. The sessions will enable leaders across camps to share experiences and continue to build their skills.

## 5.4 Control Condition

Half of camps randomly selected in to the study (n=30) will be assigned to the control condition. Camps in the control condition will receive an HIV prevention training to be administered after the 30 month follow-up data collection with the intervention group. The 2-3 hour session will focus on how HIV is transmitted,

# 6. STUDY PROCEDURES AND ADVERSE EVENTS

## 6.1 Overview

This study will use both quantitative and qualitative methods to assess the impact of the study. We will closely document the process of the intervention implementation in order to better understand successes and failures and to facilitate replication if efforts are effective.

The behavioral survey will be conducted using Computer-Assisted Personal Interview (CAPI) at baseline and follow-up sessions. Each round of surveys will be conducted with all eligible camp members selected in to the study. We will measure changes in gender norms and risky sexual behavior as well as other potential mediators of STI risk listed in the Objectives section of this document. STI tests will be administered with a subset of men at baseline and all men at the 30 month follow-up. We will measure relative prevalence among men in the intervention as compared to those in the control condition.

## 6.2 Pre-test Survey

Prior to launching the baseline survey we will pre-test all survey items and CAPI procedures with fifty camp members who have similar demographic characteristics to our target population but are not members of an intervention or control camp. The reasons for this pre-test is to assess length of the survey, check response distribution, assess item comprehension, and pilot data collection procedures.

## 6.3 Baseline Behavioral Survey

Eligible and consenting camp members in the 60 selected camps will participate in a survey assessing socio-demographics, outcome variables and mediating variables (Appendix D). The questionnaires will be administered using CAPI by trained interviewers who are not intervention facilitators. The interview will last approximately 60 minutes. Interviews will take place in a central location within each ward, interviewers split between the four wards. Participants who appear influenced by drugs will be asked to return for an interview the next day.

## 6.4 Baseline STI Assessment

Urine and blood specimens for diagnosis of STIs (NG, TV, CT) and HIV will be requested from a sample of half of sexually active men at baseline. Baseline testing is employed to compare base prevalence in the two conditions.

## 6.5 Follow-up Behavioral Assessments

At 12 months and 30 months a follow-up behavioral assessment will be conducted among eligible and consenting participants in order to assess outcome and mediating variables. We will use the same procedures described for the baseline assessment.

## 6.6 Follow-up STI Assessment

At 30 months urine and blood specimens will be collected from 100% of eligible and consenting men for STI testing (NG, TV, CT) and HIV.

## 6.7 Qualitative Assessment

We will conduct 3 focused qualitative studies during the study period with a sub-sample of eligible and consenting members of intervention camps. Each study will have a different focus pertaining to HIV, IPV, or microfinance experience. The purpose of these studies will be to inform ongoing intervention activities. The results may be used to inform the booster training for health leaders as well as the microfinance information session that the loan officers do during the repayment sessions.

## 6.8 Process Evaluation

Process evaluation measures will be collected in both the microfinance and health leadership components through the life of the intervention.

### 6.8.1 Intervention Training

To ensure integrity and consistency of the microfinance and health leadership trainings, we will measure dose of intervention delivered, dose received by participants, participant satisfaction, implementation fidelity, reach, and other contextual factors. These analyses will result in real-time suggestions to improve intervention implementation, as well as provide data to better connect effectiveness of implementation and study outcomes.

### 6.8.2 Microfinance Repayment Monitoring

We will collect data on participation and repayment success at each weekly repayment meeting for all participating camp members. Loan Officers will collect this data in an Access database at each session to enable review of individual success as well as to aggregate results by camp over time. We will produce camp-specific monthly reports that show: rate of participation in loan access; rate of meeting attendance; rate of loan installment repayment; total savings deposited; rate of loan repayment completion; rates of dropout and default; and reasons for dropout or default.

### 6.8.3 Camp Health Leaders Monitoring

Camp Health Leaders will be required to keep a log of conversations they have with camp members pertaining to HIV and Gender-Based Violence. Logs are intended to measure the number of conversations, the topics of conversation, and any challenges faced. Forms will be collected monthly and used to inform booster sessions. Urgent challenges reported by CHLs will be followed up more frequently with direct contact from study staff.

## 6.9 Language of Interviews

The predominant language in the study area is Kiswahili. All survey interviews will be conducted in Kiswahili, with selected answers recorded on CAPI tablets. The CAPI instrument is able to switch between Kiswahili and English, thus enabling questions and responses to be viewed in both languages. Qualitative interviews will be conducted in Kiswahili and will be recorded. Recorded interviews will be transcribed and translated into to English.

## 6.10 Quality Assurance

Responsibilities for data quality assurance will be shared with the site and UNC staff. The various data collected through the life of this study will be checked, cleaned, and stored appropriately after each wave of data collection (See Section 8). For the first objective – enumerate and characterize camps – the Tanzania field coordinator and data entry staff will be responsible for removing entries that are redundant or where 50% or more of data is missing for a specific camp. For camps selected in to the study the Research Coordinator will work with camp leaders to get up-to-date camp rosters and ensure enough members are eligible and willing to participate to make the camp eligible for study participation. Data shared between sites will be de-identified, encrypted and transferred as password protected files.

Behavioral and biological data collected via CAPI and biological specimens will have several quality check procedures implemented. A hard copy contact form for each participant will record the unique Participant Identification Number, records of each attempted contact made to the participant, and final data collection result. Identical records will be entered in to the CAPI device. Nurses will keep separate records of all individuals that completed the baseline and endpoint behavioral surveys as well as whether or not participants provided specimens for STI testing. All survey data collected on CAPI will be stored initially in locked files with the data manager in Delhi, India. Copies of survey data will be locked within CAPI devices themselves, accessible only with password provided by the data manager. Duplicating records of behavioral and biological data collection results on hard copy and CAPI serve as a data check to ensure all data collected is accounted for.

## 6.11 Adverse Events and Social Harms

All adverse events or unanticipated problems associated with the procedures of any component of this study will be reported within the time frames required by the Institutional Review Boards at UNC and MUHAS. Appropriate action will be taken to address any adverse events or incidents. Reports will be made using standard forms available from the relevant IRBs for adverse events or unanticipated problems associated with the study procedures or participating individuals.

Participants will be provided with information on how to contact the local research staff to report such events as breaches of confidentiality, HIV-related disruption of families, acts of discrimination, and physical harm. We will ask participants to return to the research site or otherwise contact research staff in order to make such reports as well as receive referrals to mitigate potential harm.

If staff members experience occupational exposure to HIV, the incident will be reported and a protocol will be followed to minimize their risk of being infected with HIV that includes the provision of post-exposure prophylaxis with antiretroviral drugs when indicated. All staff at risk for occupational exposure to HIV will be trained on universal precautions and on the post-exposure prophylaxis protocol. All staff performing HIV tests will require training and certification on performing the test and on quality assurance.

Adverse events reporting in this trial has been considered in the context of several important characteristics of the study. First, this is a minimal-risk study as defined in federal regulations—“the probability and magnitude of harm or discomfort anticipated in the research are not greater in and of themselves than those ordinarily encountered in daily life or during the performance of routine physical or psychological examinations or tests.” Second, all participants in this study are de-identified using Personal Identification Numbers. Participant data will not be linked to names. We have chosen this approach to maximally protect the confidentiality of those volunteering for HIV testing and counseling. Third, although no clear guidance is currently provided in regulation for adverse events reporting in behavioral studies such as this one, as opposed to clinical trials of drugs or devices, we are taking the steps outlined above within the context of our study.

All study staff/volunteers having potential contact with study participants or data will receive human subjects training.

## 6.12 Security and Confidentiality

Strict security procedures will be followed to ensure confidentiality and security of data for all components of the study. Study documentation will be kept in locked files in study site offices. To ensure confidentiality, questionnaire data will be encrypted and saved on password-protected secure computers. Personal identifiers will not be stored in the data set and all computers will be protected by antivirus software.

# 7. EVALUATION OF OUTCOMES

## 7.1 Overview

This is a cluster-randomized controlled trial to measure the efficacy of a combined microfinance and health promotion intervention. A total of 60 camps will be randomized to receive the intervention or the control. The primary endpoint will be STI prevalence measured with biological assessments at baseline and at 30 months, and gender based violence perpetration and HIV-risky behaviors measured at baseline, 12 and 30 months with behavioral assessments.

## 7.2 Primary Endpoint - STI Prevalence

For the proposed study, men will provide a first-catch urine specimen for NG, CT and TV testing by polymerase chain reaction (PCR). The introduction of nucleic acid amplification tests (NAATs), including PCR for the detection of sexually transmitted pathogens, has provided critical new tools for diagnosis of infections with Neisseria gonorrhea (NG), Chlamydia trachomatis (CT) and Trichomonas vaginalis (TV). NAATs are substantially more sensitive than culture or antigen detection assays, and specific detection of multiple STI pathogens in men is possible using traditional urethral specimens and, importantly, in noninvasive urine specimens. Study participants who are identified with NG, CT, or TV will be offered treatment according to the standard of care in Tanzania: NG (Ceftriaxone), CT (Doxycycline), TV (Metronidazole).

Blood will be obtained for serologic tests to detect antibodies indicating infection with HIV. We will use Immnochromatographic tests (ICT), which are Alare Determine ^TM^ HIV 1/2 as Test 1 and Unigold ^TM^ HIV as Test 2. An Immuno-Chromatographic test (ICT) is used for the qualitative detection of antibodies to the human immunodeficiency virus (HIV) type 1 and 2 in blood. The blood sample flows along the strip and if HIV antibodies are present, the antigen/antibody complex binds with a conjugate forming red band. If antibodies to HIV are absent no red band is formed. To ensure assay validity, a procedural control band is incorporated into assay device.

### 7.2.1 Training, Quality Assurance, and Project Expertise for Lab

QA will be performed on each new lot of rapid test kits that arrives at sites by running the controls and testing one positive and one negative sample of known sera. To ensure the validity of each HIV test, a procedural control line is incorporated into the strip or device and is labeled “Control”. The control line must appear in all tests, whether positive or negative. Internal controls will be used in NG, CT, and TV tests to ensure validity.

### 7.2.2 Specimen Collection

Good Clinical Practices will be followed by all nurses collecting biological specimens to ensure the safety of participants and self. For each participant providing blood nurses will put on new gloves and select a new syringe and needle. After sanitizing the area of needle insertion, the nurse will draw at least 1 mL blood in a 2mL syringe and transfer to a 4 mL container. For urine specimen collection participants will be instructed to fill a 30 mL container up to a pre-marked 20 mL line with first catch urine. Both containers will be labeled with biological ID numbers and stored in a cooler at 18-25 degrees C until the end of the day.

### 7.2.3 Specimen Handling, Storage, and Shipping

Specimen (both urine and blood) will be transported from the field to MUHAS lab every day. Nurses will make sure each collected specimen has been stored in cooler boxes and ready for transportation. The Lab Coordinator will count and transport all collected specimen, and ensure transport at a temperature between 18 -25 degrees C to MUHAS. Blood samples will be processed at the MUHAS lab.

Urine sample will be and frozen to -20 C upon arrival at the MUHAS lab. At the end of each week the frozen specimens will be transported to the NIMR Mwanza laboratory for processing and further storage. There will be a tracking form for both urine and blood specimen.

Extra specimen will be stored in a -20 C freezer in the MUHAS lab should a re-test of any specimen be necessary.

## 7.3 Primary Endpoint - Gender-based Violence Perpetration

We will measure violence using the WHO violence against women instrument.([Garcia-Moreno, Jansen, Ellsberg, Heise, & Watts, 2006](#_ENREF_28)) This instrument is built on methodological work and research on violence by partners using the Conflict Tactics Scale. We have used these questions previously to measure young men’s perpetration of violence in Tanzania.([Maman, Yamanis, Kouyoumdjian, Watt, & Mbwambo, 2010](#_ENREF_51)) Psychometric analyses of these items reveal that they load on two factors, physical and sexual violence, and both factors are internally consistent. We will also include questions on emotional abuse from this scale. The physical violence items include instances of slapping, pushing, hitting, kicking, choking and threatening with a weapon. Sexual violence items include physically forcing a partner to have sexual intercourse or physically forcing a partner to do something sexual that is degrading or humiliating. Emotional abuse questions include instances of insulting, belittling in front of others, intimidating, and threatening to hurt someone the partner cared about. Men will be asked if they have ever perpetrated these instances with their current or any other partner. For questions answered ‘yes’, men will be asked how many times they have perpetrated these instances in the last 12 months as well as before the last 12 months. These questions will be followed up with the same questions but asked as the men as victim of these instances rather than perpetrator. We also included a question on physical violence and one on sexual abuse before age 12.

## 7.4 Secondary Outcomes - Behavioral Outcomes

All secondary outcomes except for microfinance outcomes will be measured at baseline, 12 months and 30 months. Microfinance outcomes will be measured at 12 and 30 months only.

### 7.4.1 HIV risk behaviors

**Consistent condom use with recent partners**

For each of their three most recent sexual partners, men will report how many times they engaged in sex (oral, anal, vaginal) with these partners during the past month, and for how many of these times they used a condom.

**Number of partners in past year and lifetime**

We will ask men to report their overall number of lifetime sexual partners and number of partners they have had in the past year. Several studies have shown that young men with more lifetime partners are more likely to have perpetrated violence towards their sexual partners.([Dunkle et al., 2004](#_ENREF_21); [Harrison, O'Sullivan, Hoffman, Dolezal, & Morrell, 2006](#_ENREF_29); [Maman et al., 2010](#_ENREF_51)) Thus, we will include lifetime number of partners as a mediating variable in our violence analysis. In addition, we will analyze men’s number of lifetime partners as an outcome, indicating risk of HIV transmission to women.

**Sexual debut**

We will ask men to report whether they have ever had vaginal or anal intercourse and the age of first intercourse after they report initiating sexual activity.

**HIV testing**

We will ask men to report at each assessment whether they have ever been tested for HIV, tested within the past 12 months (since last assessment), and whether they received their test results. HIV testing is one of the key HIV prevention strategies that will be promoted by the health leaders. In our previous work, we found that 25% of our population of sexually active youth aged 15-19 had ever been tested for HIV.

**Sexual concurrency**

We will assess concurrency during the two most recent sexual partnerships using the direct method. Participants are asked if they had sex with anyone else while in a sexual partnership with either of their two most recent partners. In our previous work in Tanzania, we found that the direct method was concordant with the UNAIDS calendar method at least 82% of the time([Eaton, 2009](#_ENREF_23)). We will ask participants how many sexual relationships they had at the same time during the past 6 months.

Concurrency in the last 12 months will also be assessed using an indirect method. Participants will be asked to list nicknames for their three longest sexual relationships in the past 12 months. For each partner mentioned participants will answer how recently they last had sex with each partner, when they first had sex with each partner, and if they are still having sex with each partner.

**Gender Equity**

We will use the Inequitible Gender Norms subscale of the Gender Equitable Men’s Scale (GEMS) validated by Shattuck et al. in Tanzania and Ghana ([Shattuck et al., 2013](#_ENREF_83)). The scale demonstrated good reliability in two populations of adult men in Tanzania (Cronbach’s a = .72 and .87). The scale was developed by Pulerwitz and Barker ([Julie Pulerwitz & Gary Barker, 2008](#_ENREF_71)), but the validated measure used in Tanzania and Ghana eliminates two questions that measured attitude towards homosexuality. Two other questions presented in the first person were changed to men in general to match all other questions. The scale asks participants do indicate how much they agree with scenarios describing roles and relations of men to women on a 4-point Likert scale from 1 = Strongly Agree to 4 = Strongly Disagree.

**Gender Role Conflict**

We will use 16 items from the Gender Role Conflict Scale (GRCS), developed and originally validated by O’Neil et al. in the United States to measure the impact of socialized gender roles on men (O’Neil et al., 1986). The scale initially demonstrated good internal consistency (Cronbach’s a = .75-.85). Since then, hundreds of studies have used the GRCS in the United States and globally and have assessed its validity and reliability. A recent review of over 200 studies confirmed good internal consistency (Cronbach’s a ranging from .70 to .89) and construct validity, with moderate factor intercorrelation (.35-.68). The review found the measure was positively correlated with depression, anxiety, stress, as well as measures of self-esteem, substance use, and shame (O’Neil, 2008; Wester et al., 2012).

**HIV Stigma**

We will incorporate three questions about camp-level perceptions of HIV from work in Uganda done by Singer and Charlebois ([Singer](#_ENREF_85)). These questions ask participants to answer how many camp members are likely to avoid people with HIV, if HIV is the result of wrong behaviors, and if people with HIV have brought shame on their families. Responses are on a 4-point Likert scale from 1 = No People to 4 = Most People.

**Alcohol and Drug Use**

Participants will be asked about lifetime use of alcohol and drugs. Participants who have used alcohol will be asked about use in the last month, frequency of use, frequency of use until intoxication, and if consumed greater than 5 drinks in one sitting. Participants who have used drugs will be asked about use and frequency of use in the last 12 months.

**Attitudes towards HIV Risk Behaviors and Intimate Partner Violence**

We will ask participants their opinion on questions related to behaviors towards sex and sexual partners. These will be asked directly after similar questions assessing injunctive norms in their social network. Questions will ask opinions on condom use, concurrent partners, and whether there are occasions where hitting or being hit by a partner is ok. Responses are on a 4-point Likert scale from 1 = Strongly Agree to 4 = Strongly Disagree.

**Experience of Peer and Neighborhood Violence**

We will ask participants about their experiences and perpetration of peer violence and about their perceptions of neighborhood violence and safety using three questions from a longitudinal study examining influences of adolescent health risk behaviors in North Carolina (Context Linkages Study; NIDA R01-DA13459; PI Susan T. Ennett). In addition, we will use one question from the 135-item Victimization Scale developed by Nadel et al. in New York (1991). This scale measures exposure to violence and victimization in the home, at school, and in the community and is included in CDC’s compendium of assessment tools for measuring violence-related attitudes, behaviors, and influences among youth.

**Self-Control**

Self-control is a strong predictor of sexual risk behavior and IPV perpetration, and has been found to mediate the effects of community and family characteristics on violence perpetration. To assess self-control, we will use impulsivity and temper items from Grasmick et al.’s Self-Control Scale (1993). The scale was validated among males and females by Piquero and Rosay (1998) and was found to have good internal consistency (Cronbach’s a=.71).

**Sexual Network**

We will assess the sexual behaviors of participants with up to three partners with whom they have had the longest sexual relationship in the last year. Participants will first be asked to give the nickname of these partners, then will be asked about relationship longevity, confidence in condom negotiation, condom use, alcohol use before sex, concurrency, confidence in discussing HIV, HIV testing, and physical abuse.

**Perceived Social Support**

We will use the Adolescent Perceived Microsystems Scale for Social Support to assess emotional and material support ([Seidman et al., 1995](#_ENREF_81)) This scale, including a measure of support for sexual health information, demonstrated high reliability among youth in rural Kenya (α = .95).([Puffer et al., 2010](#_ENREF_69)) This study ranked received support on a 4-point Likert scale. For our study participants will answer Yes or No regarding support they receive from family members, relatives, peers and sexual partners.

**Social Network Norms (For partner violence, condom use, HIV testing and concurrency)**

We hypothesize that the positive influence of peers will promote reductions in risk behavior by participating camp members. To obtain information regarding peer influence, we first will ask participants to identify all members of the camp that they know, then to select their three closest friends out of those selected. For each of the three people mentioned, we will ask the participant to describe whether the person uses condoms consistently, has ever perpetrated violence towards their partners, has had an HIV test, has ever engaged in at least two sexual partnerships at the same time, and whether or not the friend condones these behaviors in others.

**Camp Norms**

We will also ask participants questions about norms in the camp as a whole, using four questions informed by the Add Health Survey, which was used to assess adolescents’ perceptions of their peers’ substance use (Henry et al., 2011).

**Moral Agency**

At midpoint and endpoint we will assess moral agency in protecting against HIV transmission. Questions will be adapted from the Personal Responsibility Scale. ([Wolitski, Flores, O’Leary, Bimbi, & Gomez, 2007](#_ENREF_99)) Factor analysis of the scale revealed two factors, personal responsibility and partner responsibility. Computed item-to-total correlations for each scale were high and statistically significant. Personal responsibility questions were internally consistent (a = .85), while internal consistency for partner responsibility was relatively poor (a = .58). These questions ask participants about individual responsibility in protecting others against HIV. Responses are on a 5-point Likert scale from 1 = Strongly Agree to 5 = Strongly Disagree.

## 7.5 Mediating Variables/Outcomes

Mediating variables will be measured at baseline, 12 months and 30 months.

**Hope**

We will use Snyder’s State Hope Scale ([C. R. Snyder et al., 1991](#_ENREF_88)), a 6-item measure that has been shown to be internally consistent among several populations.([C. R. Snyder et al., 1996](#_ENREF_89)) Hopelessness among young urban men is associated with greater violence later in adolescence ([Stoddard, Henly, Sieving, & Bolland, 2011](#_ENREF_91)). We translated the measure into Kiswahili and found that a 5-item version was reliable in our R21 (n = 75; α = .73). We will use the 5-item version.

**Future Orientation**

We will use the 15-item Future Outlook Inventory developed by Cauffman and Woolard ([Cauffman & Woolard, 1999](#_ENREF_13)). The inventory uses items from the Life Orientation Task (Scheier & Carver, 1985), the Zimbardo Time Perspective Scale (Zimbardo, 1980), and the Consideration of Future Consequences Scale (Strathman, Gleicher, Boninger, & Edwards, 1994). Initial psychometric properties for this measure were established with a non-incarcerated sample. With that sample, the reliability coefficient = .71 and the measure correlated with self-reports of risk perception among high school and college students.

The Future Outlook Inventory asks participants to rank from 1 to 4 (1= Never True to 4= Always True) the degree to which each statement reflects how they usually are (e.g., I will keep working at difficult, boring tasks if I know they will help me get ahead later). Higher scores indicate a greater degree of future consideration and planning.

The Future Outlook Inventory will be supplemented with the six-item revised Life Orientation Test (LOT-R) ([Scheier, Carver, & Bridges, 1994](#_ENREF_80)). The six item version showed internal reliability (α = .78) and was found to be fairly stable over time with samples of U.S. college students.

**Social Cohesion**

We will use an adapted measure of social cohesion developed by Sampson, Raudenbush & Earls ([R. J. Sampson, Raudenbush, & Earls, 1997](#_ENREF_78)). Participants will respond to statements related to cohesion of camp members on a 4-point Likert scale ranging from 1 = strongly disagree to 4 = strongly agree. The statement included “people in my camp are willing to help each other” and “the members of my camp share the same values”. Two negatively framed questions will be reversed to the positive for ease of use. Kim et al. used a similar measure of social cohesion in an analysis of the relationships between neighborhood collective efficacy and adolescent sexual behavior in the US ([J. Kim, 2010](#_ENREF_42)).

**Collective efficacy** (Reciprocated Exchange)

Collective efficacy measures the extent to which people believe in their ability to act cooperatively to solve problems. Our goal in providing microfinance opportunities to camp members is to enhance the camp’s collective efficacy to achieve similar health outcomes. We will use the 5-item collective efficacy measure of reciprocated exchange (alpha = .65) ([R.J. Sampson, Morenoff, & Earls, 1999](#_ENREF_77)), which has been validated in other settings. For our measure we will substitute the word “neighborhood” with “camp,” and add a question about the frequency with which camp members visit their camp. Participants will be asked how often activities takes place within his camp on a 4-point Likert scale from 1 = Very Often to 4 = Never. Activities included having get-togethers as well as asking each other advice about personal things. To assess reciprocated exchange, each participant was asked how often a set of six activities takes place within his camp. Activities included having get-togethers as well as asking each other advice about personal things.

**Emotional Distress**

The HSCL-25 ([Hesbacher, Rickels, Morris, Newman, & Rosenfeld, 1980](#_ENREF_31)) includes the anxiety and depressive dimensions of the HSCL-58, derived from the Symptom Checklist-90 and originally developed by Derogatis *et al.* ([Derogatis, Lipman, Rickels, Uhlenhuth, & Covi, 1974](#_ENREF_16)). The items are scored on a scale from 1 (not bothered) to 4 (extremely bothered), and a mean score of 1.75 has been defined as the cut-off point for ‘caseness’ ([Winokur, Winokur, Rickels, & Cox, 1984](#_ENREF_98)) and has been validated for Southeast Asian versions of the HSCL-25 ([Mollica, Wyshak, de Marneffe, Khuon, & Lavelle, 1987](#_ENREF_58)).

We will use a version of the HSCL 25 translated into Kiswahili and implemented in Tanzania ([Lee, Kaaya, Mbwambo, Smith-Fawzi, & Leshabari, 2008](#_ENREF_48)). The measure inventories symptoms of anxiety and depression in 25 items (10 related to anxiety, 15 to depression). When implemented in antenatal clinics, the measure showed Good internal consistency for the total scale (a = 0.90) and for the depression subscale (a = 0.88), but marginal internal consistency (a = 0.76) for the anxiety subscale. Participants will respond to how much symptoms bothered them on a 4-point Likert scale from 1 = Not at All to 4 = Extremely.

**Work and Expenditures**

Questions on work and expenditures will be incorporated to assess changes in participants exposed and not exposed to the microfinance component. These questions will be derived from the Tanzania National Panel Survey’s (TNPS) Multi-topic Household Questionnaire ([W. Bank, 2012](#_ENREF_7)). Design and implementation of the TNPS is supported by the Living Standards Measurement Study-Integrated Surveys on Agriculture. Only questions relating to work and expenditures will be included.

**Adverse Childhood Experiences**

We will assess adverse childhood experiences during participants’ first 18 years of life using the Adverse Childhood Experiences International Questionnaire (ACE-IQ). This includes questions adapted from the Conflict Tactics Scale. For categories adapted from the CTS, response categories are “never,” “once” “a few times,” “many times.” The ACE-IQ includes questions on 13 categories of adverse childhood experiences: emotional abuse; physical abuse; sexual abuse; violence against household members; living with household members who were substance abusers; living with household members who were imprisoned; growing up with one or no parents; parental separation, or divorce; emotional neglect; and bullying. Though the measure is relatively new, it has been field tested in China, the Philippines, Saudi Arabia, South Africa, and Vietnam. It is currently being validated in six countrywide studies and has shown to be positively correlated with adverse health conditions in Vietnam (Tran et al., 2015).

**Household Hunger**

We will use four items from the Household Food Insecurity Access Scale (HFIAS), developed by FHI 360 through USAID’s Food and Nutrition Technical Assistance (FANTA) project. The scale has been used in several countries and can be used to assign households, individuals, and populations to a level of food insecurity, from food secure to severely food insecure. The scale was validated by Kneuppel et al. in Tanzania, where it showed good internal consistency (Cronbach’s a = 0.90) among a sample of 237 households and 21 key informants. Construct validity was verified in the same sample through factor analysis, with constructs explaining 69% of variance. Food security was positively associated with education and household wealth status and negatively associated with maternal age and household size (Kneuppel et al., 2009)

## 7.6 Process Outcomes

Intervention process outcomes and monitoring data collection will occur throughout the life of the study. Monitoring data will be collected ongoing through the two year intervention period. Additional process outcomes will be collected during behavioral data collection at midpoint and endpoint.

### 7.6.1 Microfinance Process Outcomes

Data will be collected during midpoint and endpoint surveys to assess rationale for loan uptake or non-uptake and effects of accessing the loan. Microfinance borrowers will be asked about businesses they started or supported with the loan, how they have benefited or suffered from taking the loan, and reasons for any difficulties in repaying the loan.

### 7.6.2 Microfinance Monitoring

Data will be collected at each weekly repayment session to monitor microfinance participation and repayment success. These data will be aggregated at the camp level to compare with other camps. Data to be collected on this form include:

**Attendance**

Data will be collected at weekly loan repayment meetings on whether or not individuals accessing loans attended weekly mandatory meetings.

**Loan Acquisition**

We will keep a roster for all camp members eligible for the microfinance intervention (completed baseline survey), and compare this to the total number of individuals who fulfill requirements and access loans.

**Loan Repayment**

Data will be collected at weekly loan repayment meetings on whether or not individuals paid installments. We will also monitor individuals who have completely paid back loans.

**Savings Deposited**

Participants accessing loans are required to contribute to personal savings for reserve in case of non-payment, and encouraged to deposit additional savings each week. We will monitor cumulative savings deposited.

**Loan Default and Dropout**

Repayment data will be monitored over time to determine whether or not individuals defaulted on their loan. We will also monitor participants that accessed a loan, repaid the loan completely, but decided not to access further loans.

**Types of businesses**

We will assess the type of businesses that men initiate with their microfinance loans.

**Profit earned from business**

We will assess how much profit, if any, men have earned from their business.

### 7.6.3 Camp Health Leadership Process Outcomes

Data will be collected at midpoint and endpoint to assess leadership characteristics of Camp Health Leaders and their reach in implementing the health leadership portion of the intervention. These questions were developed from a review of numerous publications on peer leadership ([Flanagan, Williams, & Mahler, 1996](#_ENREF_25); [Mason-Jones, Flisher, & Mathews, 2011](#_ENREF_53); [Pearlman, Camberg, Wallace, Symons, & Finison, 2002](#_ENREF_62); [Perry, Klepp, Halper, Hawkins, & Murray, 1986](#_ENREF_63)).

Nominated CHL will be shown a list of people they know in their camps and asked who on the list they have talked with about HIV or GBV. CHL will then be asked the frequency of HIV or GBV questions in the week prior to the assessment. Leadership characteristic questions will include CHL confidence in conversing about HIV and GBV, as well as how much they agree that the CHL training increased their knowledge of HIV and GBV, interpersonal skills, compassion towards others, and leadership skills. CHL will also be asked about their comfort in their role as CHL and talking about HIV and GBV, as well as whether they believe they are taken seriously in their role.

### 7.6.4 Camp Health Leaders Monitoring

We will ask health leaders to record the number and types of the conversations that they have with camp members related to HIV and GBV. CHL will be provided diaries divided by week and asked to record the number of individual(s) talked to, how many were male and female, the topic of conversation, and any challenges encountered. Records of conversations will be collected bi-weekly at loan repayment meetings or by visiting CHLs at camps. Urgent challenges will be followed up with phone calls, and trainers will use non urgent challenges to generate topics for booster sessions.

## 7.7 Qual*i*tative Outcomes

We will conduct 3 focused qualitative studies through the life of the project with a sub-sample of eligible and consenting participants. Each study will have a different focus pertaining to HIV risk, GBV perpetration, or intervention experience. Qualitative study topics include:

1. Intimate Partner Violence
2. Leadership and Microfinance
3. Childhood Experiences and Adult Conflict

The study team will convene during the intervention period and at the end of the intervention period to review interview transcripts and develop a coding scheme that identifies broad themes and subthemes related to the study’s research questions. Members of the study team will read the transcripts multiple times to refine the codes and will practice using the coding scheme on a selection of transcripts to ensure that all relevant text is captured appropriately by a code. After the coding scheme has been developed, a staff of coders will be trained to apply the codes to the transcripts. To ensure intercoder reliability, a subset of data will be double-coded. The study team will resolve coding discrepancies. Codes will be entered into ATLAS.ti. Using these codes, the study team will retrieve and analyze text relevant to the research study questions.

# 8. DATA COLLECTION AND ADVERSE EVENT REPORTING

Both quantitative and qualitative data will be collected, and we have developed a data management plan for each. Quantitative data will include results from the baseline demographic and behavioral survey, follow-up behavioral data collected at months 12 and 30, and STI assessments at baseline and month 30.

## 8.1 Data Management Center

The study team at the University of North Carolina at Chapel Hill will serve as the Data Management Coordinating Center. All behavioral and biological data will be entered on Samsung galaxy tablets and compiled by the Data Manager in Delhi, India. UNC study staff will work with the Data Manager to ensure the proper collection, storage, and transfer of data to UNC for cleaning and analysis.

## 8.2 Quantitative Data Management Plan

### 8.2.1 Data Security

The CAPI system we are using for survey collection and STI results has multiple tiers of security. A password must be entered to activate the tablet computer and a second password must be entered to access the data capture software.  There are different levels of access for interviewers, field supervisors, and system administrators.  Only users with administrator level access will have the ability to delete data that had been previously captured.  The data captured on the device will be in a machine-readable (xml) format and is not easy to interpret without using a computer program. In such a case, data stored on the device is archived in a separate directory and is encrypted using 128 bit AES encryption. Thus, any data stored on the device is inaccessible unless decrypted. The decryption key is not stored on the device and is maintained by the central data management team. There is also an option available to delete the data from the device after uploading to the Data Manager. As an additional security measure, lost or stolen tablets can be tracked and have their data erased remotely by data management staff in India when the lost or stolen tablet is connected to a Wi-Fi or cellular network.

All data captured by the tablets will be transmitted to the database server based in India using HTTPS, which is a widely used communications protocol for secure communication over a computer network. HTTPS provides authentication of the web app and associated web server that one is communicating with, which protects against malicious attacks. Additionally, it provides bidirectional encryption of communications between a client and server, which protects against eavesdropping and tampering with and/or forging the contents of the communication.

Once the data reaches the data server, it is then stored on a secure server. The server support RAID-1 storage in which a complete copy of the data is stored on multiple independent disk drives as a precaution against disk failure. The database is configured to make automated backups and logs of all modifications.  Additional monthly backups of the database are stored on external media such as DVD disks, which are under lock and key. Access to the server and database are restricted to authorized personnel with a valid password.

#### 8.2.2 Data Transfer and Analysis

Behavioral data collected on Samsung Galaxy tablets will be compiled by the Data Manager in Delhi, India. Following each phase of data collection the de-identified data will be sent to UNC and MUHAS in password protected files for cleaning and validation. Study staff from UNC will coordinate with field staff in Tanzania to clean and validate the data. Cleaned and validated data will be kept at UNC for analysis using SAS SURVEYLOGISTIC version 9.2. Analyses will be shared via password protected files with field staff. Data files will not contain identifying information on study subjects, but will contain a Personal Identification number to allow for checking of original paper forms for errors that might be identified.

All members of the study team potentially involved in data cleaning, analysis, or interpretation will be guided by a set of principles: (1) timely and rigorous analysis and dissemination of study data is a major responsibility the project has to the study participants and the research field, (2) project members should submit brief concept sheets using a standard forms proposing analyses that they wish to take the lead on, (3) other individuals may be given the opportunity to become involved in proposed analyses at the discretion of project PIs (4) all PIs will be given access to the cleaned data sets, both in the U.S. and in host countries, (5) data dispersed to project team members will be blinded of any identifying information to assure confidentiality, (6) there will be an opportunity to review all papers published from the project by all PIs, (7) there are many important means of dissemination of study results, including publications, presentations at meetings, and also in-country dissemination activities, all which should be supported, (8) that junior members of the overall study team should be given the opportunity to analyze and disseminate data with mentoring by senior members of the team, and (9) that full public access to the raw data should be made in a timely manner once the study has been completed and major papers have been disseminated.

#### 8.2.3 Data Collection Forms

**Community Informant and Camp Verification Interviews**

Community Informant and Camp Verification Interviews will be completed by Research Assistants in the field with pen and paper. The Community Informant form solicits the names of camps from community members of the four study wards. The Camp Verification form collects GPS coordinates and characteristics of existing, active camps found in during the Community Informant interviews. All completed interviews will be collected by the Research Coordinator at the end of each day and delivered to the data entry team at MUHAS for entry in to an Access database. Physical forms will be stored in a locked cabinet in the field office.

**Behavioral Survey**

An approximately 1 hour behavioral survey instrument will be created and formatted in Samsung Galaxy tablets. The survey will be comprised of variables that assess, but are not limited to, demographic data, HIV risk and protective behaviors, social and sexual networks, gender-based violence perpetration and victimization, hope, collective efficacy, emotional distress, social support, alcohol and drug use, and work and expenditures. The behavioral survey form will be used at baseline, and 12 and 30 month follow-ups.

**Participant Contact Form**

Logs of participant contact and survey status will be used to monitor whether or not participants have completed the survey, refused, or follow-up is needed. Similar logs will be kept by nurses to monitor uptake of STI testing among those selected to participate. These forms will be kept in locked cabinets at all times.

**Lab Transport Form**

Lab transport forms will be used to monitor the movement of biological specimens between collection sites, storage sites, and sites of analysis. Forms for each individual participating in specimen collection will receive a de-identified alpha-numeric label to track individual specimens. Lab result forms will be returned to study staff with the same alpha-numeric code to link to behavioral data.

### 8.3 Qualitative Data Management Plan

Study staff are responsible for the development of standardized field guides for the collection and coding of qualitative data. These data will be translated from local language into English and then transcribed and typed into computer files for analysis. Audio recorders will be procured for the study site for use by staff. The Intervention Coordinator will supervise transcription and translation staff, and there will be regular quality assurance checks conducted to assure consistency in methods of translation and transcription. Strict security procedures will be followed to ensure confidentiality and security of qualitative data. The final data files will be shared with U.S. staff via password protected files. Study documentation will be kept in locked files at the field study site. The transcriptionists will be trained to blind any identifying information of study participants as data is entered into textual computer files.

# 9. STATISTICAL CONSIDERATIONS

## 9.1 Quantitative Statistical Analysis Plan

## 9.1.1 Sample Size, Power Calculations and Effect Size

The choice of 60 camps randomized into two groups is based upon evidence generated in our previous study and likely incidence estimates available from published studies with similar populations in Tanzania and other countries in Africa. We estimate average camp sizes of 36.2 sexually active males and 52 total males. STI measures of ulcer, discharge, any symptom, and any STI were used as possible sexual outcome measures, with condom use and sexual and/or physical violence perpetration our behavioral outcome measures. Based upon our previous study we computed intraclass correlation (ICC) estimates between 0.00 and 0.01 for sexually transmitted diseases and 0.00-0.032 for behavioral measures. Given average camp sizes and ICC estimates we have inflated sample size estimates assuming simple random sampling with design effects ranging from 1.4 to 2.3. All sample estimates assumed an attrition of 20%. Given a sample size of 1955 sexually active males and 2808 total males in 54 camps we will have 80% power (2-sided, α=0.05) to detect the protective effect of the intervention (OR) of 0.32 for ulcer, 0.59 for discharge, 0.61 for any STI symptom, 0.50 for syphilis, and 0.66 for any STI. We will be able to detect protective effects of 0.74, 0.68 and 0.65 in condom use for those sexually active, for all males, and for sexual or physical violence perpetration in all males, respectively. Statistical power to detect mediation is expected to be high based on the simulation results of Fritz and MacKinnon where a total sample size of 539 was sufficient for .80 power to detect the most conservative simulated mediation effect.([Fritz & MacKinnon, 2007](#_ENREF_27))

### 9.1.2 Aim 1 Sampling and Analytical Technique

Aim 1 is to enumerate and characterize camps where young men at risk for HIV acquisition and transmission socialize in Dar es Salaam using the PLACE methodology.

**Primary analysis.** A list of camps in the four wards within Dar es Salaam will be collected through Community Informant interviews. A minimum of 450 interviews will be conducted with individuals located in the four wards of focus in Dar es Salaam. Research assistants will systematically move through each *mtaa* (street) in the ward to cover sufficient geographical space in each ward. Research assistants will return to each camp mentioned by community informants to verify whether or not it exists; if found to exist, camp characteristics and GPS coordinates will be collected. Frequencies of camp characteristic categories will be analyzed with SAS SURVEYLOGISTIC version 9.2.

### 9.1.3 Aim 2 Sampling and Analytical Technique

Aim 2 is to determine whether men in camps randomized to receive a microfinance and health leadership intervention have a lower prevalence of sexually transmitted infections (NG, TV, CT) and report perpetrating less physical or sexual violence against female sexual partners as compared to men in camps not randomized to receive the intervention.

Data gathered from cluster randomized studies such as this one are correlated by common group membership and therefore will be analyzed using methods appropriate to such designs. Since all male camp members in selected camps will be eligible for inclusion (p=1.0), a simple random sample of camps with randomization based upon an equal probability method will be conducted. Sampling weights will be computed as the inverse of probabilities of selection with non-response weights over each wave computed as the inverse of response rates. We will compare odds ratios across group at the 12 month assessment for behavioral outcomes and at 30 months for both behavioral and STI outcomes using SAS SURVEYLOGISTIC version 9.2 to incorporate both the complex nature of sample selection and weighting. Controls for covariates will also be introduced where necessary.

### 9.1.4 Mediation Sampling and Analytical Technique

In the event that program effects are identified, we will estimate path coefficients in multiple mediator models and generate bootstrapped confidence intervals for total and specific indirect effects of one or more mediator models using the Mplus program Version 6.11.([Pituch, Stapleton, & Kang, 2006](#_ENREF_65)) We will assess simple and multiple mediation in the relationships between the microfinance intervention condition, intervention mediators including hope, future orientation, social cohesion, social support, emotional distress, gender equity, conflict, alcohol and drug use, HIV stigma, perceived social support, collective efficacy, sexual relationships and risk behavior, attitudes towards HIV risk and intimate partner violence, work and expenditures, sexual relationships and risk behavior, and social and sexual network norms.

## 9.1.5 Treatment of Missing Data

We will examine the pattern of missingness due to loss to follow-up and apply an appropriate multiple imputation strategy if deemed necessary.([Schafer, 1997](#_ENREF_79); [Trials, 2010](#_ENREF_94)) We will use the propensity score method for multiple imputation and the Markov chain Monte Carlo approaches to multiple imputation of binary outcome data.([Rubin & Schenker, 1986](#_ENREF_75)) We will conduct sensitivity analyses that quantify the robustness of inferences to departures from underlying assumptions.([Molenberghs & Kenward, 2007](#_ENREF_56); [Molenberghs, Verbeke, Thijs, Lesaffre, & Kenward, 2001](#_ENREF_57); [Trials, 2010](#_ENREF_94)).

## 9.2 Qualitative Analysis Plan

Interviews will be conducted by local staff who have been hired and trained in qualitative research methods. All in-depth interviews with men from camps will be audio-taped, transcribed, translated, coded and computerized for analysis. Analysis will begin during data collection so that topics for further exploration can be incorporated into ongoing fieldwork. Qualitative data analysis consists of searching for patterns in data and conceptualizing ideas that help explain the presence of those patterns.([Bernard, 1995](#_ENREF_9)) Analysis of textual data will involve five steps: (1) reading for content; 2) deductive and inductive coding; 3) data display to identify emerging themes; 4) data reduction; and 5) interpretation.

# 10. ETHICS AND PROTECTION OF HUMAN SUBJECTS

## 10.1 Institutional Review

Prior to implementation of any study component at a site, the study strategy, informed consent and recruitment forms, and any other forms used with participants must be approved by the IRB at both UNC and in Tanzania. All form amendments and any other documents affecting the safety and welfare of study participants must be approved by both IRBs prior to implementation. The study site Principal Investigator is responsible for the preparation and submission of all documents and periodic reports required by an IRB.

## 10.2 Informed Consent

**Aim 1**

Enumerating and characterizing camps includes two stages of data collection. The first includes Community Informant interviews with community members in the fours wards of study; the second includes Camp Verification interviews with camp members. Since no personal data will be collected in this phase of the study, informed consent will not be collected.

**Aim 2**

HIV risk, gender-based violence perpetration, and their mediators will be assessed at baseline, 12 and 30 months. STI prevalence assessments will occur at baseline and 30 months. Participants selected to provide a biological specimen must have completed the behavioral assessment to be eligible. Separate written consent will be necessary for the behavioral and biological assessments. Potential participants will be excluded from participation based on an inability to provide informed consent. All consent documents will be stored in locked cabinets. Copies of the informed consent script will be provided to prospective participants to keep.

Research assistants will begin each interview by confirming the eligibility of participants. All eligible participants will then be read the informed consent script in Kiswahili and given the chance to ask questions or clarifications on what they are being asked to do in the study. Participants will acknowledge their consent by initialing the line for behavioral assessment and writing the date and their signature on the form.

Upon completion of the survey, participants selected for the biological assessment will meet with nurses who will administer the biological consent. Nurses will explain the purpose and procedure for blood and urine collection in private. Participants will acknowledge their consent by initialing the line for biological assessment.

Participants not selected for the biological assessment will have the opportunity to receive a symptomatic STI assessment with nurses. Nurses will explain the procedure for symptomatic STI assessment. Participants will acknowledge their consent by initialing the line for symptomatic STI assessment.

Written informed consent will be obtained from each participant in the qualitative sub-studies, prior to their participation. Participants will be provided with a copy of their consent form.

The host site has prior experience in obtaining informed consent for prevention trials within the cultural context. The informed consent procedure for this study has been designed to maximize understanding of potential risks. Consent forms will be translated into Kiswahili and piloted with 50 participants to ensure the script is understandable.

If there are cultural, literacy, or political reasons why signing the informed consent form is not appropriate, individuals will be allowed to mark consent forms with an “X”. At this point, any misunderstandings regarding procedures, risks, or benefits can be clarified. Individuals will be provided with information on how to contact the study staff to report adverse events associated with participation in any component of the study. Study staff will have been trained in the need to ensure individuals provide voluntary informed consent.

## 10.3 Confidentiality

### 10.3.1 Local Protections

Confidentiality of all study participants will be strictly maintained across all study components. All field staff who have not completed research ethics training will attend the one-day FHI 360 Research Ethics Training Curriculum for Community Representatives conducted by trained staff during the Training of the Trainers Meeting at the start of the project. A major component of the training involves a presentation and discussion of the principles of research ethics, with emphasis given to confidentiality. Field staff must pass the FHI 360 Research Ethics Training Curriculum for Community Representatives post-test to participate in data collection with participants. <http://www.fhi.org/en/RH/Training/trainmat/ethicscurr/retccr.htm>.

Staff conducting interviews for Aim 1 will select private areas at camps to ensure participant confidentiality. Upon arriving at camps, interviewers will scan the camp landscape to find the appropriate location for interview location. In the case that no such place exists, the interviewer will consult with camp leaders to arrange private areas for interviews to maintain confidentiality.

During the baseline behavioral assessment participants will be asked to provide written consent to participate in the intervention. Participants will be informed that they will be re-contacted to participate in follow-up behavioral assessments and may be selected for STI testing. Upon providing written consent to participate in the study, the participant will provide contact information. For the study components in which participants are required to provide written informed consent, all study data, laboratory specimens, reports, and study data collection, process, and administrative forms will be identified by a coded number only, to maintain participant confidentiality. All study data will be stored separately from study records that contain names or other personal identifiers (such as informed consent forms or locator forms). For post-test support services no identifying information will be linked to the participant's STI test results.

Forms, lists, logbooks, appointment books, and any other listings that link participant ID numbers to other identifying information must be stored in a separate, locked file in an area with limited access at the study site headquarters. Participant names and corresponding participant IDs entered into a computer database must be password protected and must be maintained in a directory separate from any study-specific data. File encryption is encouraged, but not required when stored on a password protected device.

For participants who provided written informed consent, study-related information will not be released without the written permission of participants.

### 10.3.2 Statistical and Data Management Protections

Access to all study databases housed with the Data Manager in Delhi, India and UNC will be password protected to ensure confidentiality of study participants. Any copies of data query forms, utilization logs, and all adverse event reports will be stored in locked file cabinets. Only study PIs and the data research coordinator will have access to secure documents. Any breach in confidentiality protocol will immediately be written up as an adverse event, and submitted to the IRB. The data to be analyzed at UNC only include anonymous study IDs with no other information identifying the subjects. The databases are stored electronically on university computers protected by a firewall and allow only local access using a password.

## 10.4 Benefits

**10.4.1 Individual Benefits**

- All study participants will have the opportunity to either learn their status on a number of STIs (NG, CT, TV) or receive symptomatic assessments of STIs. Participants who test positive for any STI will be provided with treatment free of charge and access to a trained psychologist as needed. Participants exhibiting STI symptoms will be referred to clinics that provide free treatment.
- Participants may benefit from knowing that the information learned from this study will help to improve the situation of youth like themselves.
- Participants may also benefit from talking with the trained peer leaders about social concerns that they face.

### 10.4.2 Community Benefits

- This trial tests the efficacy of a combined microfinance and HIV prevention trial in reducing HIV and gender-based violence perpetration by men in a country with high HIV prevalence, which if successful, could be disseminated more broadly in urban Tanzanian camps. This study is designed to answer an important scientific question about the ability of a community-based, behavioral HIV intervention to reduce the incidence of HIV in high-prevalence area (an estimated 2.7% of females and 1.2% of males aged 15-24 years are HIV-infected in Tanzania) ([TACAIDS & NBS OCGS, 2013](#_ENREF_93)). The outcome of this study could have a significant public health impact in communities and countries around the world.
- The population of focus in this study is young, urban men. Identifying venues to access male social networks, especially in urban settings where HIV prevalence is often highest and men are not being reached, is a challenge. Camps, the intervention unit in this study, provide potential venues to access social networks of men.

### 10.4.3 Benefits to Humanity

If the trial is successful there are potentially enormous social benefits that can be gained from the study.  We expect this intervention to be effective, cost-effective, sustainable and easily disseminated in other developing country settings.  Youth in sub-Saharan Africa and elsewhere continue to be disproportionately affected by the HIV epidemic, and there are limited strategies that have proven effective at reaching youth.  If proven efficacious, we feel this multi-level approach to reaching young men at risk for HIV transmission and acquisition will have widespread application in other settings.

## 10.5 Risks

### 10.5.1 Individual Psychosocial Risks

We believe the risk of participants experiencing negative psychological events as a result of participation in the study will be infrequent.    It is possible that some participants may experience these outcomes as a result of responding to the survey questions or participation in the health promotion component of the intervention trial.   We will train our interviewers in techniques of interviewing to help minimize the embarrassment and emotional distress that men may feel responding to personal questions about their relationships.  Health leaders from camps will also be trained in techniques to help minimize the embarrassment that men may feel as a result of participating in the health promotion component of the study.  It is also possible that men may experience some social or psychological risks if they learn they are positive for a sexually transmitted infection.  Nurses who will take the specimens and provide the results to the men will be trained in appropriate counseling procedures to communicate and discuss results with the men.  If men have social or psychological concerns beyond what nurses feel they are capable of responding to the nurses will be trained to refer these men to the site PI, Lusajo Kajula, who is a trained psychologist with experience working with adolescents.

### 10.5.2 Individual Physical/Health Risks

We believe that the specific research components in this study will not subject participants to any physical or health risks. Since the intervention includes a microfinance component there is the possibility that participants will experience financial gain. The possibility exists that participants with financial gain could be targeted for robbery, and thus potentially harmed physically. We believe this risk to be unlikely and had no such occurrences of robbery or mugging during a pilot study we conducted with a microfinance component. There is also the possibility that individuals taking a microfinance loan may not have successful businesses and become indebted. To minimize this risk numerous safeguards are in place to promote successful repayment (must attend business training, must have loan approved by group and camp leader, must deposit minimal savings), as well as booster sessions to help address business challenges. Taking a loan is voluntary and does not disclude individuals from further participation in the study. Nurses collecting blood samples with hypodermic needles may face occupational exposure to HIV and other STIs through accidental needle sticks. All nurses employed for this study are trained in proper precautionary procedures to prevent these accidents, and have been informed of post-exposure procedures to prevent infection.

### 10.5.3 Community Risks

Through participation in the study, camps may experience stigma, misperceptions, and/or negative rumors. For example, participating camps may be viewed as having a high prevalence of HIV as the reason for its inclusion in the study. These camps may also be perceived to be receiving special benefits beyond those outlined above; it may be perceived as receiving favored treatment. All these may lead to ostracism of and discrimination toward these camps and its members by neighboring communities.

## 10.6 Adverse Event Reporting

All adverse events and incidents associated with the procedures of this study will be reported within the time frames required by the appropriate Institutional Review Boards. Appropriate action will be taken to address any adverse events or incidents.

## 10.7 Study Withdrawal and Discontinuation

The study may be discontinued at any time by the NIH. Study participants will be informed though the process of informed consent that they may withdraw from the study at any time for any reason. Participants may be withdrawn from the study by the site Principal Investigators if they are found to have:

- An obvious psychological/psychiatric disorder that would invalidate the informed consent process, or otherwise contraindicate participation in the study; or
- Any other condition which in the opinion of the study site Principal Investigator will interfere with achieving the study objectives. In such cases the Principal Investigator will review the reasons for withdrawal with the study team prior to participant notification. The Principal Investigator may decide to include data collected prior to participant withdrawal in study analyses.

## 10.8 Incentives for Participation

Participants will be reimbursed TSH 10,000 (~$6.25) for the transportation costs incurred to travel to the interview site and for the time that they spent in transit to and during the interviews. This includes participation in the baseline behavioral and biological assessment, and the 12 and 30 month follow-ups. Health Leaders nominated from camps will receive TSH 10,000 (~$6.25) for each day present of the 5-day training. Likewise, all camp members eligible for the microfinance component will receive TSH 10,000 (~$6.25) for each day present during the business and entrepreneurship training.

## 10.9 Linkages to Care

All stages of this trial are planned in the context of recognized ethical principles for protecting human participants in international research. Participants who test positive for an STI during the baseline or 30 month assessment will receive free treatment and will have access to a trained psychologist if desired. Participants who are symptomatically positive for an STI will also be offered free treatment.

In order to track access to the above interventions, we will make a list of health facilities (public, nongovernmental, private) in each community and will determine which types of HIV-related prevention and treatment interventions are available in each facility by interviewing facility managers at baseline and updating the list annually. This activity will be the responsibility of the Study Site Project Director at each site.

## 10.10 Inclusion of Women

Because men are the primary targets in this study, we have focused on them in the details of this protocol. However, women are included as participants in all stages of this trial and all will be able participate in behavioral assessments. Diagnostic STI tests will be completed with men only, and women will be offered a syndromic STI assessment and free care if the assessment is positive. Both men and women will have access to a trained psychologist should they desire counseling after a positive assessment. Female members of intervention camps will be eligible for microfinance and to be elected as a Camp Health Leaders. This strategy is aimed to maintain the leadership and social support inherent in camps. While we acknowledge that the risks of physical and social harms are greater for women than men, we do not believe that the exclusion of women from the trial based on this potential risk could be justified.

## 10.11 Inclusion of Minorities

This cluster-randomized trial will be conducted in urban camps within four wards of Dar es Salaam Tanzania. All study participants are Black African. The intervention component will be applied to all members of camps assigned to this condition. All camps verified in these four wards that meet inclusion criteria (size, length in existence, and safety) are eligible for participation. All camp members meeting individual criteria (age, length of membership, plans to remain the city for 30 months, willingness to provide locator information) will be eligible for inclusion in the study.

## 10.12 Inclusion of Children

This trial targets young, urban men in Dar es Salaam, Tanzania. All phases of this trial require a minimum age of 15 to participate. This cut-off was chosen to maximize inclusion of young men who are newer in developing patterns of sexual relationships, to capture the range of men who are members of camps, and to determine how the intervention works with different aged men

# 11. LABORATORY SPECIMENS AND BIOHAZARD CONTAINMENT

## 11.1 HIV Testing

As HIV and other blood-borne infectious agents can be transmitted through contact with contaminated needles/lancets, blood, or blood products, universal precautions will be employed by all personnel involved in this study. Safety standards reflect those required by the Occupational Safety and Health Administration (OSHA) and recommended by the CDC. Policies for biohazard prevention and response, provision of postexposure prophylaxis for staff members having possible exposures to HIV-infected body fluids, and disposal of biological/hazardous waste will be followed. Good Clinical Practices will be followed by all nurses collecting biological specimens to ensure the safety of participants and self.

# 12. ADMINISTRATIVE PROCEDURES

## 12.1 Community Preparedness and Involvement

The study will conduct specific community preparedness activities in all wards being considered for inclusion in the research. The community preparedness activities will commence prior to communities’ randomization into the study, and will continue throughout the course of the study, with the goals of preparing communities for possible involvement in the study, maintaining constructive relationships with the communities over the course of the study, and ensuring that the research is responsive to community expectations and needs.

The first step in preparing for the study is for field staff to introduce themselves and the study concept to stakeholders. Prior to any data collection the Research Coordinator will meet with local ward officials to explain the study and how recruitment will occur. Initial data collection to identify and verify camps in the wards will be coordinated with these local ward officials. The Research Coordinator will also be responsible for explaining the study to camp leaders contacted for verification and provide updates on procedures for random selection and assignment.

After getting to know the communities socially and geographically, the study team can begin to build the Community Advisory Board (CAB). For this study the CAB will be composed of local government leaders, camp leaders, and parents of camp members. All participating wards and camps will be represented in the CAB. The primary purposes of the CAB are to provide the study team with input on plans of study implementation and serve as a community liaison between researchers and the community. As CAB members become more familiar and knowledgeable with our staff and project, there will be greater participation on study-specific issues where members may have a range of suggestions, concerns, and advice to offer. CAB meetings will be held periodically throughout the life of the project to discuss project progress.

Communities will be actively prepared for the initiation of the study. This will include ensuring that the randomization process is as transparent as possible. Extensive explanation of the community randomization process will be conducted with the CAB. The concepts underlying randomization and its importance in interpreting the study results will be elucidated. The results of the randomization will be provided to the CAB in a clear manner, and any questions or concerns regarding the results for the randomization will be addressed promptly by study personnel.

## 12.2 Study Coordination

The University of North Carolina at Chapel Hill will serve as the Operational Coordinating Center. In this capacity, UNC will be responsible for convening the study team on conference calls, setting agendas and meetings, recording and distributing minutes, setting the study timeline, interacting with NIMH, preparing the study protocol, and ensuring that the relevant IRB regulations are followed.

The Muhimbili University of Health and Allied Sciences will be responsible for coordinating and implementing all phases of the study. This will include community engagement, trainings, data collection, data transfer, and intervention implementation and follow-up. During critical implementation phases throughout the course of study the UNC PI or study coordinators will travel to Tanzania to work with the field based study team. This will allow them to visit the communities at each of the sites and discuss and collaborate on arrangements for the study from the vantage point of the actual experience of those sites.

## 12.3 Statistical and Data Management Coordinating Centers

All data collection and transfers to the Data Manager will be conducted by MUHAS. The study Data Manager in Delhi, India, Basant Singh, will provide oversight and advise both sites in local data management. Dr. Singh has been responsible for data collection design, consolidation, and quality assurance for a number of large clinical trials in Tanzania. UNC will be primarily responsible for statistical analysis, including oversight of analysis conducted at MUHAS.

## 12.4 Study Site Monitoring

### 12.4.1 Intervention

Day-to-day monitoring, supervision, and support of the microfinance and health leadership intervention components will be led by the intervention coordinator, who will periodically observe microfinance repayment sessions and meet with Camp Health Leaders to discuss progress in implementing health promotion training within the camps. The intervention coordinator will supervise loan officers and solicit periodic reports on progress of the microfinance component in the camps.

### 12.4.2 Data Management

Study PIs will generate a list of criteria to be used for on-site data management quality control and monitoring. Study staff responsible for collecting and transferring data to the data manager will be in regular contact with the data manager for data monitoring and review.

### 12.4.3 Documentation

Regular monitoring of study documentation will be performed by UNC and MUHAS to ensure proper recording, reporting, and storage of all study documentation.

## 12.5 Protocol Compliance

This study will be conducted in full compliance with the protocol. Amendments to the protocol will be reviewed and validated by study staff at both UNC and MUHAS. Protocol amendments requiring IRB approval will be submitted to relevant IRBs by the study sites’ Principal Investigators, and approval obtained prior to implementing the amendment.

## 12.6 Minimizing Risks to Staff

All staff members obtaining and handling blood samples for the study will have received training in proper procedures to minimize risks associated with occupational exposure to blood-borne pathogens. All staff will follow standard safety procedures and will have appropriate supplies available to minimize such risks. Protocols will be in place to provide postexposure prophylaxis (PEP) for any study-related occupational exposures to HIV.

## 12.7 Investigator Records

All project primary source documents will be maintained in a secure location for a period of five years after documents are considered complete and utilized. Primary source documents will be stored at field office locations in locked cabinets. Primary source documents will be made available to authorized personnel upon request.

## 12.8 Policy on Data Sharing

Project data sets will be housed at both UNC and MUHAS, and will be password protected. All Principal Investigators (both U.S. and host country) will be given access to the cleaned data sets. Individuals outside the primary study team are required to submit a data acquisition form that outlines desired variables. To ensure confidentiality, data dispersed will be blinded of any identifying participant information.

## 12.9 Dissemination of Study Results

Timely and rigorous analysis and dissemination of study data is a major responsibility the project has to the study participants and the research field. We recognize that there are many important means of dissemination of study results, including publications, presentations at meetings, and in-country dissemination activities.

The project PIs must give study staff permission to proceed with dissemination submissions and will provide manuscript review for publications with the goal of ensuring that all manuscripts, publications, and other products generated from the study accurately represent its design, implementation, and analysis. Both manuscripts and abstracts will require review by the PIs prior to submission to the journal/conference.

The PIs of this study will develop a list of potential publications for the study and will have first selection on publication development. Other study group members will be given the opportunity to become involved or lead in proposed analyses and dissemination activities. Study members can also propose additional analyses and publications not generated by study PIs. If approved by the study PIs, the study staff will have 6 months to produce a draft to the study team. If no product has been submitted by the end of this time period, the authors forfeit their claim to the proposed analysis, and the analysis will be assigned to new authors.

Proper recognition will be given to all study group members participating sufficiently in the publication process. Authorship of all study manuscripts will reflect standards of scientific paper authorship, including that all authors have made an intellectual contribution to the design, implementation, interpretation, and/or analysis of the study, and have participated in the study and the preparation of the manuscript sufficiently to assume public responsibility for the content of the manuscript.

All peer-reviewed manuscripts and presentations must acknowledge the funding source for this project with the following statement,

*“This research was sponsored by the U.S. National Institute of Mental Health (5R01MH098690).” In addition, authors of manuscripts may choose to acknowledge key staff members and others who were involved in the research but do not meet the criteria for authorship.*

Tool Revision History:

| ****Version Number**** | Version Date | Summary of Revisions Made: |
| --- | --- | --- |
| 1.0 | 11Feb2013 | Original version with Tool Summary Sheet |
| 2.0 | 23Sep2015 | Updated intervention implementation text |

**References**

Accion. Retrieved December 17 2010, from <http://www.accion.org>

AIDS, U. G. C. o. W. a. (2006). Keeping the promise: an agenda for action on women and AIDS. . Geneva

Almodovar, A., Tomaka, J., Thompson, S., Mckinnon, S., & O'Rourke, K. (2006). Risk and protective factors among high school students on the US/Mexico border. *American Journal of Health Behavior, 30*(6), 745-752.

Amirkhanian, Y. A., Kelly, J. A., Kabakchieva, E., Kirsanova, A. V., Vassileva, S., Takacs, J., . . . Mocsonaki, L. (2005). A randomized social network HIV prevention trial with young men who have sex with men in Russia and Bulgaria. *Aids, 19*(16), 1897.

Andrews, J. A., Tildesley, E., Hops, H., & Li, F. (2002). The influence of peers on young adult substance use. *Health psychology, 21*(4), 349.

Bank, T. W. (1998). Using microcredit to advance women. PREM notes. .

Bank, W. (2012). Tanzania National Panel Survey. <http://econ.worldbank.org/WBSITE/EXTERNAL/EXTDEC/EXTRESEARCH/EXTLSMS/0,,contentMDK:22288781~menuPK:6194952~pagePK:64168445~piPK:64168309~theSitePK:3358997~isCURL:Y,00.html>

Bartley, M., & Ferrie, J. (2010). Do we need to worry about the health effects of unemployment? *Journal of Epidemiology and Community Health, 64*(01), 5.

Bernard, H. R. (1995). *Research Methods in Anthropology*. Walnut Creek Altamira Press

Bernays, S., Rhodes, T., & Barnett, T. (2007). Hope: a new way to look at the HIV epidemic. *Aids, 21*, S5.

Bond, K. C., Valente, T. W., & Kendall, C. (1999). Social network influences on reproductive health behaviors in urban northern Thailand. *Social Science & Medicine, 49*(12), 1599-1614.

Caldwell, J. C. (2000). Rethinking the African AIDS epidemic. *Population and development review, 26*(1), 117-135.

Cauffman, E., & Woolard, J. (1999). The future outlook inventory. Instrument developed for the MacArthur Juvenile Competence Study. *Unpublished measure available from Department of Psychology and Social Behavior, University of California, Irvine*.

D'Amico, E. J., & McCarthy, D. M. (2006). Escalation and initiation of younger adolescents' substance use: The impact of perceived peer use. *Journal of Adolescent Health, 39*(4), 481-487.

D., C. L. P., & Knowlton, A. (2005). Micro-social structural approaches to HIV prevention: a social ecological perspective. *AIDS Care, 17*(S1), 102-113.

Derogatis, L. R., Lipman, R. S., Rickels, K., Uhlenhuth, E. H., & Covi, L. (1974). The Hopkins Symptom Checklist (HSCL): A self‐report symptom inventory. *Behavioral science, 19*(1), 1-15.

DiClemente, R. J. W., G. . (2003). Human immunodeficiency virus prevention for adolescents: window of opportunity for optimizing effectiveness. *Archives of Pediatric and Adolescent Medicine, 157*, 319-320.

Dilger, H. (2003). Sexuality, AIDS, and the lures of modernity: reflexivity and morality among young people in rural Tanzania. *Medical Anthropology, 22*(1), 23-52.

Dodd, R., Munck, L., Organization, W. H., Bank, W., & Poor, V. o. t. (2002). Dying for Change: Poor People's Experience of Health and Ill-health: World Health Organization.

Dowsett, G. W., Aggleton, P., Abega, S., Jenkins, C., Marshall, T. M., Runganga, A., . . . Tarr, C. M. (1998). Changing gender relations among young people: the global challenge for HIV/AIDS prevention. *Critical Public Health, 8*(4), 291-309.

Dunkle, K. L., Jewkes, R. K., Brown, H. C., Gray, G. E., McIntryre, J. A., & Harlow, S. D. (2004). Gender-based violence, relationship power, and risk of HIV infection in women attending antenatal clinics in South Africa. *The Lancet, 363*(9419), 1415-1421.

Dunkle, K. L., Jewkes, R. K., Nduna, M., Levin, J., Jama, N., Khuzwayo, N., . . . Duvvury, N. (2006). Perpetration of partner violence and HIV risk behaviour among young men in the rural Eastern Cape, South Africa. *Aids, 20*(16), 2107.

Eaton, J. (2009). Modeling and Projections: Recommendations from a meeting of the UNAIDS reference group on estimates, modelling, and projections. Nairobi, Kenya.

Fernald, L., Hamad, R., Karlan, D., Ozer, E., & Zinman, J. (2008). Small individual loans and mental health: a randomized controlled trial among South African adults. *Bmc Public Health, 8*(1), 409.

Flanagan, D., Williams, C., & Mahler, H. (1996). Peer Education in Projects Supported by AIDCSAP: A Study of Twenty-one Projects in Africa. *Asia and Latin America: AIDSCAP/FHI*.

Friedman, S. R., & Aral, S. (2001). Social networks, risk-potential networks, health, and disease. *Journal of Urban Health, 78*(3), 411-418.

Fritz, M. S., & MacKinnon, D. P. (2007). Required sample size to detect the mediated effect. *Psychological Science, 18*(3), 233.

Garcia-Moreno, C., Jansen, H. A. F. M., Ellsberg, M., Heise, L., & Watts, C. H. (2006). Prevalence of intimate partner violence: findings from the WHO multi-country study on women's health and domestic violence. *The Lancet, 368*(9543), 1260-1269.

Grasmick, H.G., Tittle, C.R., Bursik, R.J., & Arneklev, B.J. (1993). Testing the Core Empirical Implications of Gottfredson and Hirschi's General Theory of Crime. *Journal of Research in Crime and Delincquency, 30*(1), 5-29.

Harrison, A., O'Sullivan, L. F., Hoffman, S., Dolezal, C., & Morrell, R. (2006). Gender role and relationship norms among young adults in South Africa: Measuring the context of masculinity and HIV risk. *Journal of Urban Health, 83*(4), 709-722.

Helleringer, S., & Kohler, H. P. (2005). Social networks, perceptions of risk, and changing attitudes towards HIV/AIDS: new evidence from a longitudinal study using fixed-effects analysis. *Population Studies, 59*(3), 265-282.

Henry, D.B., Kobus, K., and Schoeny, M.E. (2011). Accuracy and Bias in Adolescents’ Perceptions of Friends’ Substance Use. *Psychology of Addictive Behaviors*, *25*(1), 80-89.

Hesbacher, P. T., Rickels, K., Morris, R., Newman, H., & Rosenfeld, H. (1980). Psychiatric illness in family practice. *Journal of Clinical Psychiatry*.

Initiative, A. U. Keeping the Promise: An Agenda for Action on Women and AIDS.

JEMMOTT, J. B., & Jemmott, L. S. (2000). HIV risk reduction behavioral interventions with heterosexual adolescents. *Aids, 14*(2), 40-52.

Jewkes, R., Dunkle, K., Koss, M. P., Levin, J. B., Nduna, M., Jama, N., & Sikweyiya, Y. (2006). Rape perpetration by young, rural South African men: prevalence, patterns and risk factors. *Social Science & Medicine, 63*(11), 2949-2961.

Karlan, D., & Zinman, J. (2011). Microcredit in Theory and Practice: Using Randomized Credit Scoring for Impact Evaluation. *Science, 332*(6035), 1278-1284. doi: 10.1126/science.1200138

Kelly, J. A. (1999). Community-level interventions are needed to prevent new HIV infections. *American Journal of Public Health, 89*(3), 299.

Kelly, J. A. (2004). Popular opinion leaders and HIV prevention peer education: Resolving discrepant findings, and implications for the development of effective community programmes. *AIDS Care*.

Kelly, J. A., Murphy, D. A., Sikkema, K. J., McAuliffe, T. L., Roffman, R. A., Solomon, L. J., . . . Kalichman, S. C. (1997). Randomised, controlled, community-level HIV-prevention intervention for sexual-risk behaviour among homosexual men in US cities. *The Lancet, 350*(9090), 1500-1505.

Kelly, J. A., St Lawrence, J. S., Diaz, Y. E., Stevenson, L. Y., Hauth, A., Brasfield, T., . . . Andrew, M. (1991). HIV risk behavior reduction following intervention with key opinion leaders of population: an experimental analysis. *American Journal of Public Health, 81*(2), 168.

Kelly, J. A., St Lawrence, J. S., Stevenson, L. Y., Hauth, A. C., Kalichman, S., Diaz, Y., . . . Morgan, M. (1992). Community AIDS/HIV risk reduction: the effects of endorsements by popular people in three cities. *American Journal of Public Health, 82*(11), 1483.

Khandker, S. R. (2005). Microfinance and poverty: evidence using panel data from Bangladesh. *The World Bank Economic Review, 19*(2), 263.

Kim, J. (2010). Influence of neighbourhood collective efficacy on adolescent sexual behaviour: variation by gender and activity participation. *Child Care Health Dev, 36*(5), 646-654. doi: 10.1111/j.1365-2214.2010.01096.x

Kim, J., Pronyk, P., Barnett, T., & Watts, C. (2008). Exploring the role of economic empowerment in HIV prevention. *Aids, 22*, S57.

Kim, J. C., Watts, C. H., Hargreaves, J. R., Ndhlovu, L. X., Phetla, G., Morison, L. A., . . . Pronyk, P. (2007). Understanding the impact of a microfinance-based intervention on women's empowerment and the reduction of intimate partner violence in South Africa. *American Journal of Public Health*, AJPH. 2006.095521 v095521.

Kneuppel, D., Demment, M., and Kaiser, L. (2009).Validation of the Household Food Insecurity Access Scale in rural Tanzania. *Public Health Nutrition, 13*(3), 360-367.

Krishnan, S., Rocca, C. H., Hubbard, A. E., Subbiah, K., Edmeades, J., & Padian, N. S. (2010). Do changes in spousal employment status lead to domestic violence? Insights from a prospective study in Bangalore, India. *Social Science & Medicine, 70*(1), 136-143.

Latkin, C. A., Forman, V., Knowlton, A., & Sherman, S. (2003). Norms, social networks, and HIV-related risk behaviors among urban disadvantaged drug users. *Social Science & Medicine, 56*(3), 465-476.

Leatherman, S., & Dunford, C. (2010). Linking health to microfinance to reduce poverty. *Bulletin of the World Health Organization, 88*(6), 470-471.

Lee, B., Kaaya, S. F., Mbwambo, J. K., Smith-Fawzi, M. C., & Leshabari, M. T. (2008). Detecting depressive disorder with the Hopkins Symptom Checklist-25 in Tanzania. *International Journal of Social Psychiatry, 54*(1), 7-20.

Li, F., Barrera, M., Hops, H., & Fisher, K. J. (2002). The longitudinal influence of peers on the development of alcohol use in late adolescence: A growth mixture analysis. *Journal of Behavioral Medicine, 25*(3), 293-315.

Maman, S., Mbwambo, J. K., Hogan, N. M., Kilonzo, G. P., Campbell, J. C., Weiss, E., & Sweat, M. D. (2002). HIV-positive women report more lifetime partner violence: findings from a voluntary counseling and testing clinic in Dar es Salaam, Tanzania. *American Journal of Public Health, 92*(8), 1331.

Maman, S., Yamanis, T., Kouyoumdjian, F., Watt, M., & Mbwambo, J. (2010). Intimate Partner Violence and the Association With HIV Risk Behaviors Among Young Men in Dar es Salaam, Tanzania. *Journal of interpersonal violence, 25*(10), 1855.

Martin, S. L., Kilgallen, B., Tsui, A. O., Maitra, K., Singh, K. K., & Kupper, L. L. (1999). Sexual behaviors and reproductive health outcomes. *JAMA: the journal of the American Medical Association, 282*(20), 1967.

Mason-Jones, A. J., Flisher, A. J., & Mathews, C. (2011). Who are the peer educators? HIV prevention in South African schools. *Health education research, 26*(3), 563-571.

Matasha, E., Ntembelea, T., Mayaud, P., Saidi, W., Todd, J., Mujaya, B., & Tendo-Wambua, L. (1998). Sexual and reproductive health among primary and secondary school pupils in Mwanza, Tanzania: need for intervention. *AIDS Care, 10*(5), 571-582.

McCurdy, S. A., Ross, M. W., Kilonzo, G. P., Leshabari, M., & Williams, M. L. (2006). HIV/AIDS and injection drug use in the neighborhoods of Dar es Salaam, Tanzania. *Drug and alcohol dependence, 82*, S23-S27.

Molenberghs, G., & Kenward, M. G. (2007). *Missing data in clinical studies* (Vol. 26): John Wiley & Sons Inc.

Molenberghs, G., Verbeke, G., Thijs, H., Lesaffre, E., & Kenward, M. G. (2001). Influence analysis to assess sensitivity of the dropout process. *Computational statistics & data analysis, 37*(1), 93-113.

Mollica, R., Wyshak, G., de Marneffe, D., Khuon, F., & Lavelle, J. (1987). Indochinese versions of the Hopkins Symptom Checklist-25: a screening instrument for the psychiatric care of refugees. *Am J Psychiatry, 144*(4), 497-500.

Morduch, J., & Haley, B. (2001). Analysis of the effects of microfinance on poverty reduction. *CIDA, Ottawa*.

Nadel, H., Spellmann, M., Alvaarez-Canino, T., Lausell-Bryant, L., & Landsberg, G. (1996). The cycle of violence and victimization: a study of the school-based intervention of a multidisciplinary youth violence prevention program. *American Journal of Preventive Medicine,12*(5 Suppl):109-119.

Narayan-Parker, D., Patel, R., & Bank, W. (2000). *Voices of the poor: can anyone hear us?* : Oxford University Press for the World Bank.

Noar, S. M., & Morokoff, P. J. (2002). The relationship between masculinity ideology, condom attitudes, and condom use stage of change: A structural equation modeling approach. *International Journal of Men's Health, 1*(1), 43-58.

O’Neil, J.M. (2008). Summarizing 25 Years of Research on Men’s Gender Role Conflict Using the Gender Role Conflict Scale. *The Counseling Psychologist*, *36*(3), 358-445.

Pearlman, D. N., Camberg, L., Wallace, L. J., Symons, P., & Finison, L. (2002). Tapping youth as agents for change: evaluation of a peer leadership HIV/AIDS intervention. *Journal of Adolescent Health, 31*(1), 31-39.

Perry, C. L., Klepp, K. I., Halper, A., Hawkins, K. G., & Murray, D. M. (1986). A process evaluation study of peer leaders in health education. *Journal of School Health, 56*(2), 62-67.

Perullo, A. (2005). Hooligans and heroes: youth identity and hip-hop in Dar es Salaam, Tanzania. *Africa Today, 51*(4), 75-101.

Piquero, A.R. & Rosay, A.B. (1998). The reliability and validity of Grasmick et al.'s Self-Control Scale. *Criminology, 36*(1), 157-173.

Pituch, K. A., Stapleton, L. M., & Kang, J. Y. (2006). A comparison of single sample and bootstrap methods to assess mediation in cluster randomized trials. *Multivariate Behavioral Research, 41*(3), 367-400.

Pronyk, P., Kim, J., Hargreaves, J., Makhubele, M., Morison, L., Watts, C., & Porter, J. (2005). Microfinance and HIV prevention emerging lessons from rural South Africa. *Small Enterprise Development, 16*(3), 26-38.

Pronyk, P. M., Hargreaves, J. R., Kim, J. C., Morison, L. A., Phetla, G., Watts, C., . . . Porter, J. D. H. (2006). Effect of a structural intervention for the prevention of intimate-partner violence and HIV in rural South Africa: a cluster randomised trial. *The Lancet, 368*(9551), 1973-1983.

Pronyk, P. M., Harpham, T., Busza, J., Phetla, G., Morison, L. A., Hargreaves, J. R., . . . Porter, J. D. (2008). Can social capital be intentionally generated? A randomized trial from rural South Africa. *Social Science & Medicine, 67*(10), 1559-1570.

Puffer, E. S., Meade, C. S., Drabkin, A. S., Broverman, S. A., Ogwang-Odhiambo, R. A., & Sikkema, K. J. (2010). Individual-and Family-Level Psychosocial Correlates of HIV Risk Behavior Among Youth in Rural Kenya. *AIDS and Behavior*, 1-11.

Pulerwitz, J., & Barker, G. (2008). Measuring Attitudes toward Gender Norms among Young Men in Brazil. *Men and Masculinities, 10*(3), 322.

Pulerwitz, J., & Barker, G. (2008). Measuring attitudes toward gender norms among young men in Brazil development and psychometric evaluation of the GEM scale. *Men and Masculinities, 10*(3), 322-338.

Raj, A., Santana, M. C., La Marche, A., Amaro, H., Cranston, K., & Silverman, J. G. (2006). Perpetration of intimate partner violence associated with sexual risk behaviors among young adult men. *American Journal of Public Health, 96*(10), 1873.

Robbins, R. N., & Bryan, A. (2004). Relationships between future orientation, impulsive sensation seeking, and risk behavior among adjudicated adolescents. *Journal of Adolescent Research, 19*(4), 428.

Rogers, E. M. (1995). *Diffusion of innovations*: Free Pr.

Rubin, D. B., & Schenker, N. (1986). Multiple imputation for interval estimation from simple random samples with ignorable nonresponse. *Journal of the American Statistical Association*, 366-374.

Sampson, R. J. (2003). The neighborhood context of well-being. *Perspectives in biology and Medicine, 46*(3), S53-S64.

Sampson, R. J., Morenoff, J. D., & Earls, F. (1999). Beyond social capital: Spatial dynamics of collective efficacy for children. *American Sociological Review*, 633-660.

Sampson, R. J., Raudenbush, S. W., & Earls, F. (1997). Neighborhoods and violent crime: a multilevel study of collective efficacy. *Science, 277*(5328), 918-924.

Schafer, J. L. (1997). *Analysis of incomplete multivariate data* (Vol. 72): Chapman & Hall/CRC.

Scheier, M. F., Carver, C. S., & Bridges, M. W. (1994). Distinguishing optimism from neuroticism (and trait anxiety, self-mastery, and self-esteem): a reevaluation of the Life Orientation Test. *Journal of personality and social psychology, 67*(6), 1063.

Seidman, E., Allen, L. R., Lawrence Aber, J., Mitchell, C., Feinman, J., Yoshikawa, H., . . . Ortiz-Torres, B. (1995). Development and validation of adolescent-perceived microsystem scales: Social support, daily hassles, and involvement. *American Journal of Community Psychology, 23*(3), 355-388.

Setel, P. (1999). *A plague of paradoxes: AIDS, culture, and demography in northern Tanzania*: University of Chicago Press.

Shattuck, D., Burke, H., Ramirez, C., Succop, S., Costenbader, B., Attafuah, J. D., . . . Guest, G. (2013). Using the Inequitable Gender Norms scale and associated HIV risk behaviors among men at high risk for HIV in Ghana and Tanzania. *Men and Masculinities, 16*(5), 540-559.

Sieving, R. E., Eisenberg, M. E., Pettingell, S., & Skay, C. (2006). Friends' influence on adolescents' first sexual intercourse. *Perspectives on Sexual and Reproductive Health, 38*(1), 13-19.

Singer, S. e. a. *Differences and similarities between perceived community HIV stigma and TB stigma among TB evaluation patients offered same-day HIV counseling and testing*. University of California San Francisco. San Francisco, CA. Retrieved from <http://caps.ucsf.edu/uploads/pubs/presentations/pdf/charlebois_VCT_ias08.pdf>

Sivaram, S., Latkin, C. A., Solomon, S., & Celentano, D. (2006). HIV prevention in India: focus on men, alcohol use and social networks. *Harvard Health Policy Review, 7*(2), 125-134.

Snyder, C., Cheavens, J., & Sympson, S. C. (1997). Hope: An individual motive for social commerce. *Group Dynamics: Theory, Research, and Practice, 1*(2), 107.

Snyder, C. R., Harris, C., Anderson, J. R., Holleran, S. A., Irving, L. M., Sigmon, S. T., . . . Harney, P. (1991). The will and the ways: Development and validation of an individual-differences measure of hope. *Journal of Personality and Social Psychology, 60*(4), 570.

Snyder, C. R., Sympson, S. C., Ybasco, F. C., Borders, T. F., Babyak, M. A., & Higgins, R. L. (1996). Development and validation of the State Hope Scale. *Journal of Personality and Social Psychology, 70*(2), 321.

Statistics, T. N. B. o. (2002). Population Census Dar es Salaam

Stoddard, S. A., Henly, S. J., Sieving, R. E., & Bolland, J. (2011). Social connections, trajectories of hopelessness, and serious violence in impoverished urban youth. *Journal of youth and adolescence*, 1-18.

Stroeken, K., Remes, P., De Koker, P., Michielsen, K., Van Vossole, A., & Temmerman, M. (2011). HIV among out-of-school youth in Eastern and Southern Africa: a review. *AIDS CARE-PSYCHOLOGICAL AND SOCIO-MEDICAL ASPECTS OF AIDS/HIV*.

TACAIDS, Z., & NBS OCGS, I. (2013). Tanzania HIV/AIDS and Malaria Indicator Survey 2011–12. *Dar es Salaam, Tanzania. Dar es Salaam, Tanzania: Tanzania Commission for AIDS (TACAIDS), Zanzibar AIDS Commission (ZAC), National Bureau of Statistics (NBS), Office of the Chief Government Statistician (OCGS), and ICF International*.

Tran, Q. A., Dunne, M. P., Van Vo, T., & Luu, N. H. (2015). Adverse childhood experiences and the health of university students in eight provinces of Vietnam. *Asia-Pacific Journal of Public Health*, doi:1010539515589812.

Trials, P. o. H. M. D. i. C. (2010). *The Prevention and Treatment of Missing Data in Clinical Trials*: Natl Academy Pr.

UNAIDS. (2005). Intensifying HIV Prevention: Policy Position Paper: UNAIDS.

UNFPA. (2001). Tanzania Country Report.

Weir, S. S., Pailman, C., Mahlalela, X., Coetzee, N., Meidany, F., & Boerma, J. (2003). From people to places: focusing AIDS prevention efforts where it matters most. *Aids, 17*(6), 895.

Wester, S.R., Vogel, D.L, O’Neil, J.M., and Danforth, L. (2012). Development and evaluation of the Gender Role Conflict Scale Short Form. *Psychology of Men & Masculinity, 13*(2), 199-210.

Winokur, A., Winokur, D. F., Rickels, K., & Cox, D. S. (1984). Symptoms of emotional distress in a family planning service: stability over a four-week period. *The British Journal of Psychiatry, 144*(4), 395-399.

Wolitski, R. J., Flores, S. A., O’Leary, A., Bimbi, D. S., & Gomez, C. A. (2007). Beliefs about personal and partner responsibility among HIV-seropositive men who have sex with men: measurement and association with transmission risk behavior. *AIDS and Behavior, 11*(5), 676-686.

Yamanis, T. J., Maman, S., Mbwambo, J. K., Earp, J. A. E., & Kajula, L. J. (2010). Social venues that protect against and promote HIV risk for young men in Dar es Salaam, Tanzania. *Social Science & Medicine*.

Youm, Y., & Laumann, E. O. (2002). Social network effects on the transmission of sexually transmitted diseases. *Sexually transmitted diseases, 29*(11), 689.

**APPENDIX A**

Vijana Vijiweni II/YOSEFO: Entrepreneurship Training Schedule

| DAY | TIME | TOPIC |
| --- | --- | --- |
| Monday | 8:30 a.m. – 9:00 a.m. | Registration |
|  | 9:00 a.m. – 10:30 a.m. | Introduction and Training Overview |
|  | 10:30 a.m. – 11:00 a.m. | Tea |
|  | 11:00 a.m. – 1:00 p.m. | Entrepreneurship |
|  | 1:00 p.m. – 2:00 p.m. | Lunch |
|  | 2:00 p.m. – 3:30 p.m. | Entrepreneurship (Continued) |
|  | | |
| Tuesday | 8:30 a.m. – 9:00 a.m. | Review of Day 1 |
|  | 9:00 a.m. – 10:30 a.m. | Skills for Entrepreneurship |
|  | 10:30 a.m. – 11:00 a.m. | Tea |
|  | 11:00 a.m. – 1:00 p.m. | Business Environment |
|  | 1:00 p.m. – 2:00 p.m. | Lunch |
|  | 2:00 p.m. – 3:30 p.m. | Marketing and Sales Techniques |
|  | | |
| Wednesday | 8:30 a.m. – 9:00 a.m. | Review of Day 2 |
|  | 9:00 a.m. – 10:30 a.m. | How to Start a Business |
|  | 10:30 a.m. – 11:00 a.m. | Tea |
|  | 11:00 a.m. – 1:00 p.m. | Ways to Expand a Business |
|  | 1:00 p.m. – 2:00 p.m. | Lunch |
|  | 2:00 p.m. – 3:30 p.m. | Ways to Expand a Business (Continued) |
|  | | |
| Thursday | 8:30 a.m. – 9:00 a.m. | Review of Day 3 |
|  | 9:00 a.m. – 10:30 a.m. | Regulation of Credit |
|  | 10:30 a.m. – 11:00 a.m. | Tea |
|  | 11:00 a.m. – 1:00 p.m. | Procedures for Loan Repayment |
|  | 1:00 p.m. – 2:00 p.m. | Lunch |
|  | 1:00 p.m. – 3:30 p.m. | Procedures for Loan Repayment (Continued) |
|  |  |  |
| Friday | 8:30 a.m. – 9:00 a.m. | Review of Day 4 |
|  | 9:00 a.m. – 10:30 a.m. | How to Estimate Cost |
|  | 10:30 a.m. – 11:00 a.m. | Tea |
|  | 11:00 a.m. – 1:00 p.m. | Pricing |
|  | 1:00 p.m. – 2:00 p.m. | Lunch |
|  | 1:00 p.m. – 3:30 p.m. | Pricing (Continued) |
|  | | |
|  | 3:00 p.m. – 3:30 p.m. | Group Formation |

**APPENDIX B**

First Loan Application Fees and Repayment Plan

**
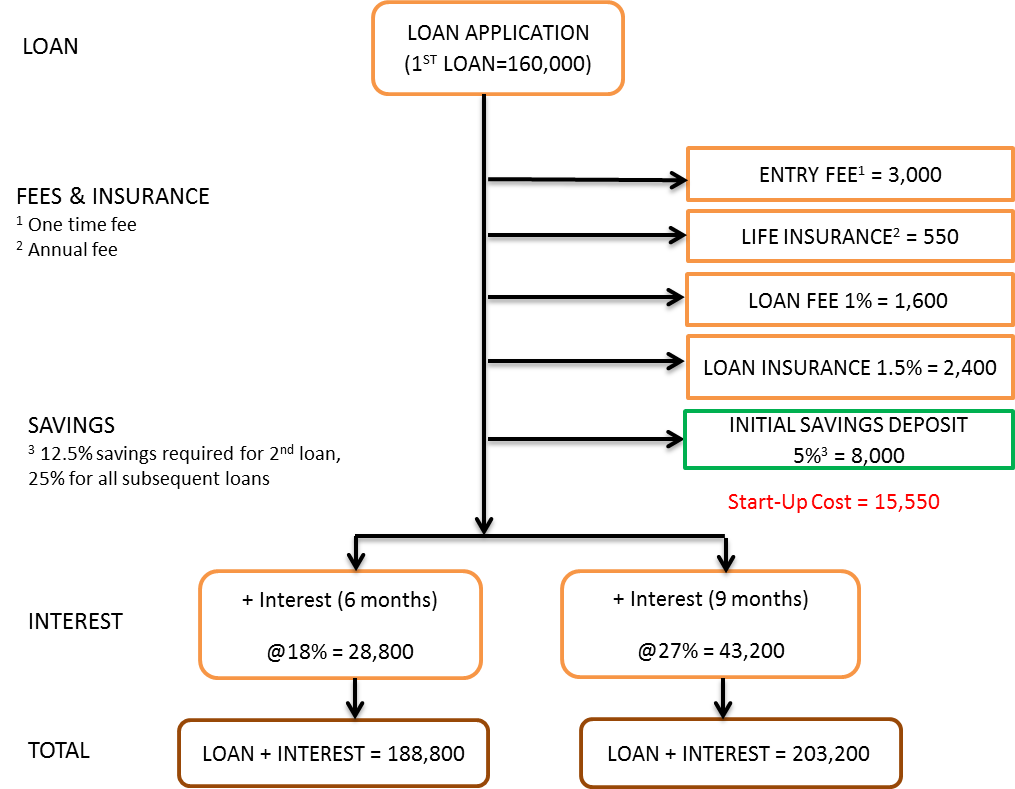
**

**APPENDIX C**

Camp Health Leader Training Schedule

| **Time** | **Activities** | | | **Responsible Personal** | | |
| --- | --- | --- | --- | --- | --- | --- |
| **Day 1: Introduction, Leadership, Introduction to HIV/AIDS** | | | | | | |
| 8:00 – 8:15 | | Registration  [Pre test] | | Ms Gema | | |
| 8:15 – 9:00 | | Welcome, Introduction & Overview of the Program | | Ms Gema | | |
| 9:00 -10:00 | | The AIDS Epidemic in the World and Your Community | | Ms Lusajo | | |
| 10:00 -10:30 | | Tea Break | | ALL | | |
| 10:30-12:30 | | HIV Myths and Facts | | Ms Lusajo | | |
| 12:30-13:00 | | Who is a Leader? | | Ms Gema | | |
| 13:00 – 14:00 | | Lunch Break | | ALL | | |
| 14:00 – 14:30 | | What are we asking you as a CHL? | | Ms Gema | | |
| 14:30 – 15:30 | | Correcting HIV/STI Myths & Communication Facts | | Mrema | | |
| 15:30 – 16:00 | | Visiting Health Facilities  Wrap-up & Evaluation | | Ms Gema | | |
| **Day 2: Gender Based Violence & Alcohol** | | | | | | |
| 8:00-8:30 | | Review | | | Edward | |
| 8:30-9:30 | | Power, Control & Violence (My Planet) | | | Ms Lusajo | |
| 9:30-10:15 | | GBV Activity (in Her Shoes) | | | Ms Lusajo | |
| 10:15-10:45 | | Tea Break | | | ALL | |
| 10:45-11:45 | | Masculinity | | | Dr Likindikoki | |
| 11:45-13:00 | | Consent Vs Coercion: Attitudes Towards Rape | | | Dr. Likindikoki | |
| 13:00-14:00 | | Lunch Break | | | ALL | |
| 14:00-15:00 | | Alcohol and HIV Risk Behaviour | | | Dr Praxeda | |
| 15:00-15:45 | | Values around Gender and Alcohol use | | | Dr Praxeda | |
| 15:45-16:00 | | Wrap-up & Evaluation of Day 2 | | | Edward | |
| **Day 3: So what? Learning about Skills: Condom Use, Problem Solving, and Crafting Messaging** | | | | | | |
| 8:00-8:30 | | | Review | | | Gema |
| 8:30-10:00 | | | Introduction on STI, Condom Demonstration and Condom Myths  [Assign HW # 1: Visit HIV/STI facilities  Assign # 2 [Buy Condoms] | | | Dr. Chuwa |
| 10:00-10:30 | | | Tea Break | | | ALL |
| 10:30-12:00 | | | Problem Solving Triggers Activity with Scenarios | | | Mrema |
| 12:00-13:00 | | | Planning for the Future and Why it matters | | | Mrema |
| 13:00-14:00 | | | Lunch Break | | | ALL |
| 14:00-15:00 | | | Characteristics of Effective HIV/STI Prev Messages | | | Mrema |
| 15:00-15:45 | | | Create Messages using “I” Statements | | | Ms Gema |
| 15:45-16:00 | | | Wrap-up & Evaluation of Day 3 | | | Ms Gema |
| **Day 4: Initiating Conversation Using Effect Prevention Messages** | | | | | | |
| 8:00-8:30 | | | Review | | | Edward |
| 8:30-10:00 | | | Practice Delivering Effective Prevention Messages  Group Activity-Formulating Prevention Messages, Presentation and Voting | | | Ms Lusajo |
| 10:00-10:30 | | | Tea Break | | | ALL |
| 10:30-11:30 | | | Countering Negative Views About Sex | | | Mr Isaac |
| 11:30-13:00 | | | Communication and Interpersonal Skills Training | | | Mr Isaac |
| 13:00-14:00 | | | Lunch Break | | | ALL |
| 14:00-15:45 | | | Role play initiating Conversation  Assign # 3 [practice Initiating Conversation] | | | Ms Gema |
| 15:45-16:00 | | | Wrap-up & Evaluation of Day 4 | | | Edward |
| **Day 5: Helping Others Overcome Barriers to safer Behavior** | | | | | | |
| 8:00-8:30 | | | Review | | | Gema |
| 8:30 -10:000 | | | Identify & Addressing Barriers to Practicing Safe Sex | | | Mrema |
| 10:00-10:30 | | | Tea Break | | | ALL |
| 10:30-12:00 | | | CHL Role play Practicing of Giving Advice | | | Lusajo/Mrema |
| 12:00-13:00 | | | Planning & Documenting Effective Conversations | | | Lusajo/Mrema |
| 13:00-14:00 | | | Lunch Break | | | ALL |
| 14:00-14:15 | | | Referral Information | | | Ms Gema |
| 14:15-15:00 | | | Next Steps and Evaluation & Conclusion  [Written Evaluation of Entire Training] | | | ALL |
| 15:00-15:30 | | | Graduation and Certificates Given  [Condom Distribution] | | | ALL |

**­APPENDIX D**

**Health Knowledge and Behavior Survey**

**Microfinance and Health Study**

Table of Contents

Section 0: Confirming Eligibility 2

Section 1: Background 3

Section 2: State of Hope Scale 5

Section 3: Future Orientation 5

Section 4: Social Cohesion 7

Section 5: Collective Efficacy 8

Section 6: Emotional Distress (HSCL 25) 8

Section 7: Gender Equitable Men’s Scale 10

Section 8: Conflict Tactics Scale 12

Section 9: HIV Stigma 19

Section 10: Alcohol and Drug Use 19

Section 11: Social Support 20

Section 12: Social Network 22

Section 13: Attitudes towards HIV Risk Behaviors and Intimate Partner Violence 26

Section 14: Sexual Relationships and Risk Behaviors 27

Section 15. Sexual Network 29

Section 16: Work and Expenditures 35

Section 17: Closing 39

Section 0: Confirming Eligibility

Interviewer:

**→ You have been chosen to participate in a study. I would like to tell you more about the study so that you can decide if you would like to participate. Before we begin, I would like to ask you a few brief questions.**

| **No.** | **Questions and Filters** | **Coding Categories** | **Codes** | **Skip to:** | **VARIABLE** |
| --- | --- | --- | --- | --- | --- |
| Q001 | [DO NOT READ OUTLOUD]  Is this participant able to provide informed consent (i.e. the person does not have a permanent physical disability (e.g. deafness) or mental disability which prevents them from providing consent)? | NO  YES | 0  1 | **END** | ELIG1 |
| Q002 | What is your date of birth?  Note: If the participant does not know his/her date of birth, please enter 1900 for the year. | (Day)___ ___ / (Mo.)___ ___ (Year)___ ___ ___ ___ |  |  | ELIG2 |
| Q003 | How old are you? | __ __ Years |  |  | ELIG3 |
| Q004 | [DO NOT READ OUTLOUD]  Is the participant older than 15 years old?  Note: If the respondent was born after today’s date in 1998, or later (Younger than age 15), then the participant is not eligible. | NO  YES | 0  1 | **END** | ELIG4 |
| Q005 | How long have you been a member of camp X?  Note: Look at the Participant Contact Form for the name of the camp | __ __ YEARS  __ __ MONTHS |  | **If Years is ≥ 2, then enter 0 for Months** | ELIG5 |
| Q006 | [DO NOT READ OUTLOUD]  Has the participant been a member of camp X for at least 3 months? | NO  YES | 0  1 | **END** | ELIG6 |
| Q007 | How often would you say you come to camp X? | EVERY DAY  SEVERAL TIMES A WEEK  ONE TIME PER WEEK  COUPLE TIMES A MONTH  ONE TIME PER MONTH  LESS THAN ONCE A MONTH | 1  2  3  4  5  6 | **END**  **END**  **END** | ELIG7 |
| Q008 | Are you a member of any other camps? | NO  YES | 0  1 | **Q012** | ELIG8 |
| Q009 | What are the names of these other camps?  *Note: If participant is a member of more than 2 other camps, separate these camp names with a comma.* |  |  |  | ELIG9 |
| Q010 | [DO NOT READ OUTLOUD]  By looking at the list of 60 study camps, determine whether participant is a member of another study camp. Is the participant a member of another study camp? | NO  YES | 0  1 | **Q012** | ELIG10 |
| Q011 | I see that you are also a member of camp X. Which of these camps would you consider to be your PRIMARY CAMP? By primary camp, I mean you spend more time in this camp than any other camp. | This Camp  Other Camp |  | **END** | ELIG11 |
| Q012 | Have you answered these types of questions and have you previously provided a sample of your blood and urine to our study staff? | NO  YES | 0  1 | **END** | ELIG12 |
| Q013 | Do you plan to live in Dar es Salaam for the next 30 months? | NO  YES | 0  1 | **END** | ELIG13 |
| Q014 | Are you willing to provide a phone number and contact information for two family members or close friends so that study staff can contact you in the future? | NO  YES | 0  1 | **END** | ELIG14 |

*Interviewer:*

**→ *Go through Informed Consent process and document informed consent on form.***

Section 1: Background

Interviewer:

→ I would like to begin our discussion by asking you some questions about yourself.

| **No.** | **Questions and Filters** | **Coding Categories** | **Codes** | **Skip to:** | **VARIABLE** |
| --- | --- | --- | --- | --- | --- |
| Q101 | Sex or gender of the participant: | Male  Female | 1  2 |  | GENDER |
| Q102 | How long have you lived in your current location?  **→** PROBE: A place where you spend at least 2 nights per week. Note that if participant has lived in the current location for his/her entire life, please enter the participant’s age here. | __ __ YEARS  __ __ MONTHS |  | **If Years is ≥ 2, then enter 0 for Months** | LIVE_D_Y  LIVE_D_M |
| Q103 | Where do you usually stay? | IN A HOME WITH MY FAMILY  IN MY OWN RENTED ROOM OR HOUSE  IN A RENTED ROOM/HOUSE WITH FRIENDS (GHETTO)  STREET  PLACE OF WORK OR BUSINESS  IN A CAMP  OTHER _____________________ | 1  2  3  4  5  6  7 |  | STAY_LOC  STAY_S |
| Q104 | Are you currently a student? | NO  YES | 0  1 |  | EDU_NOW |
| Q105 | What is the highest level of school you have completed? | NO FORMAL EDUCATION  STANDARD 4 OR LESS  STANDARD 5-7  FORM ONE  FORM TWO  FORM THREE  FORM FOUR  GREATER THAN FORM FOUR | 1  2  3  4  5  6  7  8 |  | EDUCOMP |
| Q106 | Have you ever been married? | NO  YES | 0  1 |  | MARRIED |
| Q107 | How many children have you had? | NO CHILDREN  __ __ # CHILDREN | 0 | **Q111** | CHILD_EV |
| Q108 | How many of these children are still living? | NO CHILDREN  __ __ # CHILDREN | 0 | **Note that # cannot be > response to Q107** | CHILD_LV |
| Q109 | Do you own any of the following items?  FOAM MATTRESS  BEDFRAME  COOKING POTS  CELL PHONE  RADIO  TELEVISION  COMPUTER  BICYCLE  MOTORBIKE  CAR | NO  YES | 0  1 |  | ASSET_FM  ASSET_BF  ASSET_CKP  ASSET_CELL  ASSET_R  ASSET_TV  ASSET_CMP ASSET_BC  ASSET_MB  ASSET_CR |
| Q110 | What is your role in this camp? | CHAIRPERSON  ASSISTANT CHAIRPERSON  SECRETARY  TREASURER  MEMBER  OTHER _____________________ | 1  2  3  4  5  6 |  | CAMPROLE  CMPROLE_S |
| Q111 | Are there other camps that you visit regularly? | NO  YES | 0  1 | **Q201** | CAMPHNG |
| Q112 | What are the names of these camps?  Note: If participant is a member of more than 2 other camps, separate these camp names with a comma. | OTHER CAMPS VISITED: ____________________________ |  |  | CMPHNG_S |
| Q113 | How often do you go to hang out at these other camps? | EVERY DAY  SEVERAL TIMES A WEEK  ONE TIME PER WEEK  COUPLE TIMES A MONTH  ONE TIME PER MONTH  LESS THAN ONCE A MONTH  NEVER | 1  2  3  4  5  6  7 |  | CMPHNG_F |

Section 2: State Hope Scale

*Interviewer:*

**→** Now, I would like to ask some questions about how you think about yourself right now. *Please take a few moments to focus on yourself and what is going on in your life at this moment.* I will read some statements and will ask you to respond with whether you strongly agree, agree, disagree, or strongly disagree with each statement. Now, I would like to ask you if you agree with the statement…

| **No.** | **Questions and Filters** | **Coding Categories** | **Codes** | **Skip to:** | **VARIABLE** |
| --- | --- | --- | --- | --- | --- |
| Q201 | At the present time, I am energetically pursuing my goals | STRONGLY AGREE  AGREE  DISAGREE  STRONGLY DISAGREE | 1  2  3  4 |  | HOPE1 |
| Q202 | There are lots of ways around any problem that I am facing right now | STRONGLY AGREE  AGREE  DISAGREE  STRONGLY DISAGREE | 1  2  3  4 |  | HOPE2 |
| Q203 | Right now I see myself as being pretty successful. | STRONGLY AGREE  AGREE  DISAGREE  STRONGLY DISAGREE | 1  2  3  4 |  | HOPE3 |
| Q204 | I can think of many ways to reach my current goals. | STRONGLY AGREE  AGREE  DISAGREE  STRONGLY DISAGREE | 1  2  3  4 |  | HOPE4 |
| Q205 | At this time, I am meeting the goals that I have set for myself. | STRONGLY AGREE  AGREE  DISAGREE  STRONGLY DISAGREE | 1  2  3  4 |  | HOPE5 |

Section 3: Future Orientation

*Interviewer:*

**→** Now would like to ask you some questions about how you view your life and the future. I will read you some statements and I would like you to respond with whether the statement is never true, sometimes true, often true, or always true.

| **No.** | **Questions and Filters** | **Coding Categories** | **Codes** | **Skip to:** | **VARIABLE** |
| --- | --- | --- | --- | --- | --- |
| Q301 | I think about how things might be in the future. | NEVER TRUE  SOMETIMES TRUE  OFTEN TRUE  ALWAYS TRUE | 1  2  3  4 |  | FUTUR1 |
| Q302 | I can see my life 10 years from now. | NEVER TRUE  SOMETIMES TRUE  OFTEN TRUE  ALWAYS TRUE | 1  2  3  4 |  | FUTUR2 |
| Q303 | I think often about what tomorrow may bring. | NEVER TRUE  SOMETIMES TRUE  OFTEN TRUE  ALWAYS TRUE | 1  2  3  4 |  | FUTUR3 |
| Q304 | Before making a decision I weigh the good versus the bad. | NEVER TRUE  SOMETIMES TRUE  OFTEN TRUE  ALWAYS TRUE | 1  2  3  4 |  | FUTUR4 |
| Q305 | I think about the consequences before I do something | NEVER TRUE  SOMETIMES TRUE  OFTEN TRUE  ALWAYS TRUE | 1  2  3  4 |  | FUTUR5 |
| Q306 | I like to plan things out one step at a time | NEVER TRUE  SOMETIMES TRUE  OFTEN TRUE  ALWAYS TRUE | 1  2  3  4 |  | FUTUR6 |
| Q307 | I make decisions and act without thinking about the big picture | NEVER TRUE  SOMETIMES TRUE  OFTEN TRUE  ALWAYS TRUE | 1  2  3  4 |  | FUTUR7 |
| Q308 | I think things work out better when you’ve planned for them in advance | NEVER TRUE  SOMETIMES TRUE  OFTEN TRUE  ALWAYS TRUE | 1  2  3  4 |  | FUTUR8 |
| Q309 | I run through all possible outcomes of a decision in my mind before I decide what to do | NEVER TRUE  SOMETIMES TRUE  OFTEN TRUE  ALWAYS TRUE | 1  2  3  4 |  | FUTUR9 |
| Q310 | I don’t think it’s worth it to worry about what I can’t predict | NEVER TRUE  SOMETIMES TRUE  OFTEN TRUE  ALWAYS TRUE | 1  2  3  4 |  | FUTUR10 |
| The next few questions also ask you about how you view life and the future. For these items I will ask you to respond with whether you strongly agree, agree, disagree, or strongly disagree with each statement. | | | | | |
| Q311 | In uncertain times, I usually expect the best. | STRONGLY DISAGREE  DISAGREE  NEITHER AGREE NOR DISAGREE  AGREE  STRONGLY AGREE | 1  2  3  4  5 |  | LOT1 |
| Q312 | If something can go wrong for me it will. | STRONGLY DISAGREE  DISAGREE  NEITHER AGREE NOR DISAGREE  AGREE  STRONGLY AGREE | 1  2  3  4  5 |  | LOT2 |
| Q313 | I’m always optimistic about my future. | STRONGLY DISAGREE  DISAGREE  NEITHER AGREE NOR DISAGREE  AGREE  STRONGLY AGREE | 1  2  3  4  5 |  | LOT3 |
| Q314 | I hardly ever expect things to go my way. | STRONGLY DISAGREE  DISAGREE  NEITHER AGREE NOR DISAGREE  AGREE  STRONGLY AGREE | 1  2  3  4  5 |  | LOT4 |
| Q315 | I rarely count on good things happening to me. | STRONGLY DISAGREE  DISAGREE  NEITHER AGREE NOR DISAGREE  AGREE  STRONGLY AGREE | 1  2  3  4  5 |  | LOT5 |
| Q316 | Overall, I expect more good things to happen to me than bad. | STRONGLY DISAGREE  DISAGREE  NEITHER AGREE NOR DISAGREE  AGREE  STRONGLY AGREE | 1  2  3  4  5 |  | LOT6 |

Section 4: Social Cohesion

***Interviewer:***

→ Now I’d like to ask you a few questions about how you and your fellow camp members get along in this camp. For each statement, please tell me whether you strongly agree, agree, strongly disagree, or disagree with the statement. When answering these questions, keep in mind that I’m asking you to say whether you agree with the statement based on what is true for **you**, not what other people think is true or what your camp thinks is true.

| **No.** | **Questions and Filters** | **Coding Categories** | **Codes** | **Skip to:** | **VARIABLE** |
| --- | --- | --- | --- | --- | --- |
| Q401 | People in my camp are willing to help each other. | STRONGLY AGREE  AGREE  DISAGREE  STRONGLY DISAGREE | 1  2  3  4 |  | SOCOH1 |
| Q402 | We are very close to each other in this camp. | STRONGLY AGREE  AGREE  DISAGREE  STRONGLY DISAGREE | 1  2  3  4 |  | SOCOH2 |
| Q403 | I can trust my fellow camp members. | STRONGLY AGREE  AGREE  DISAGREE  STRONGLY DISAGREE | 1  2  3  4 |  | SOCOH3 |
| Q404 | The members of my camp get along with each other. | STRONGLY AGREE  AGREE  DISAGREE  STRONGLY DISAGREE | 1  2  3  4 |  | SOCOH4 |
| Q405 | The members of my camp share the same values. | STRONGLY AGREE  AGREE  DISAGREE  STRONGLY DISAGREE | 1  2  3  4 |  | SOCOH5 |

Section 5: Collective Efficacy

***Interviewer:***

→ For the next set of questions, please tell me how often you think the following activities take place within your camp. For each statement, please tell me whether you think the activity takes place very often, often, seldom, or never.

| **No.** | **Questions and Filters** | **Coding Categories** | **Codes** | **Skip to:** | **VARIABLE** |
| --- | --- | --- | --- | --- | --- |
| Q501 | About how often do you and other camp members do favors for each other? (By favors we mean such things as lending each other small amounts of money and other small acts of kindness?) | VERY OFTEN  OFTEN  SELDOM  NEVER | 1  2  3  4 |  | COLEFF1 |
| Q502 | How often do you and people in this camp have get-togethers? | VERY OFTEN  OFTEN  SELDOM  NEVER | 1  2  3  4 |  | COLEFF2 |
| Q503 | When a camp member travels out of town, how often do you and other camp members watch over their property? | VERY OFTEN  OFTEN  SELDOM  NEVER | 1  2  3  4 |  | COLEFF3 |
| Q504 | How often do camp members visit in each other’s homes? | VERY OFTEN  OFTEN  SELDOM  NEVER | 1  2  3  4 |  | COLEFF4 |
| Q505 | How often do camp member spend time in the camp? | VERY OFTEN  OFTEN  SELDOM  NEVER | 1  2  3  4 |  | COLEFF5 |
| Q506 | How often do you and other people in your camp ask each other advice about personal things? | VERY OFTEN  OFTEN  SELDOM  NEVER | 1  2  3  4 |  | COLEFF6 |

Section 6: Emotional Distress (HSCL 25)

Interviewer:

→ I would now like to ask you about symptoms or problems that people sometimes have. Please listen to each one carefully and decide how much the symptoms bothered or distressed you in the last week, including today. Please indicate whether the symptom did not bother you at all, bothered you a little bit, quite a bit, or extremely bothered you. Now, in the last 1 week, to what extent were you bothered by…

| No. | Questions and Filters | | | Coding Categories | | Codes | Skip to: | | VARIABLE |
| --- | --- | --- | --- | --- | --- | --- | --- | --- | --- |
| anxiety sub-scale | | | | | | | | | |
| Q601 | | | Suddenly scared for no reason | | Not at all  a little  quite a bit  extremely | 1  2  3  4 |  | HSCL1 | |
| Q602 | | | Feeling fearful | | Not at all  a little  quite a bit  extremely | 1  2  3  4 |  | HSCL2 | |
| Q603 | | | Faintness, dizziness, or weakness | | Not at all  a little  quite a bit  extremely | 1  2  3  4 |  | HSCL3 | |
| Q604 | | | Nervousness or shakiness inside | | Not at all  a little  quite a bit  extremely | 1  2  3  4 |  | HSCL4 | |
| Q605 | | | Heart pounding or racing | | Not at all  a little  quite a bit  extremely | 1  2  3  4 |  | HSCL5 | |
| Q606 | | | Trembling | | Not at all  a little  quite a bit  extremely | 1  2  3  4 |  | HSCL6 | |
| Q607 | | | Feeling tense or keyed up | | Not at all  a little  quite a bit  extremely | 1  2  3  4 |  | HSCL7 | |
| Q608 | | | Headaches | | Not at all  a little  quite a bit  extremely | 1  2  3  4 |  | HSCL8 | |
| Q609 | | | Spells of terror or panic | | Not at all  a little  quite a bit  extremely | 1  2  3  4 |  | HSCL9 | |
| Q610 | | | Feeling restless, can't sit still | | Not at all  a little  quite a bit  extremely | 1  2  3  4 |  | HSCL10 | |
| **Depression sub-scale**  ***Interviewer:***  → I would now like to ask you about other symptoms or problems that people sometimes have. Similar to the questions we just answered, please decide how much the symptoms bothered or distressed you in the last week, including today. Please indicate whether the symptom did not bother you at all, bothered you a little bit, quite a bit, or extremely bothered you. Now, in the last 1 week, to what extent were you bothered by… | | | | | | | | | |
| Q611 | | Feeling low in energy, slowed down | | | Not at all  a little  quite a bit  extremely | 1  2  3  4 |  | HSCL11 | |
| Q612 | | Blaming yourself for things | | | Not at all  a little  quite a bit  extremely | 1  2  3  4 |  | HSCL12 | |
| Q613 | | Crying easily | | | Not at all  a little  quite a bit  extremely | 1  2  3  4 |  | HSCL13 | |
| Q614 | | Loss of sexual interest or pleasure | | | Not at all  a little  quite a bit  extremely | 1  2  3  4 |  | HSCL14 | |
| Q615 | | Poor appetite | | | Not at all  a little  quite a bit  extremely | 1  2  3  4 |  | HSCL15 | |
| Q616 | | Difficulty falling asleep, staying asleep | | | Not at all  a little  quite a bit  extremely | 1  2  3  4 |  | HSCL16 | |
| Q617 | | Feeling hopeless about the future | | | Not at all  a little  quite a bit  extremely | 1  2  3  4 |  | HSCL17 | |
| Q618 | | Feeling blue | | | Not at all  a little  quite a bit  extremely | 1  2  3  4 |  | HSCL18 | |
| Q619 | | Feeling lonely | | | Not at all  a little  quite a bit  extremely | 1  2  3  4 |  | HSCL19 | |
| Q620 | | Thoughts of ending your life | | | Not at all  a little  quite a bit  extremely | 1  2  3  4 |  | HSCL20 | |
| Q621 | | Feeling of being trapped or caught | | | Not at all  a little  quite a bit  extremely | 1  2  3  4 |  | HSCL21 | |
| Q622 | | Worrying too much about things | | | Not at all  a little  quite a bit  extremely | 1  2  3  4 |  | HSCL22 | |
| Q623 | | Feeling no interest in things | | | Not at all  a little  quite a bit  extremely | 1  2  3  4 |  | HSCL23 | |
| Q624 | | Feeling everything is an effort | | | Not at all  a little  quite a bit  extremely | 1  2  3  4 |  | HSCL24 | |
| Q625 | | Feelings of worthlessness | | | Not at all  a little  quite a bit  extremely | 1  2  3  4 |  | HSCL25 | |

Section 7: Gender Equitable Men’s Scale

***Interviewer:***

→ Now I’d like to ask you a few questions about what behaviors you think are acceptable in intimate relationships. For each statement, please tell me whether you strongly agree, somewhat agree, somewhat disagree, or strongly disagree with the statement. When answering these questions, keep in mind that I’m asking you to say whether you agree with the statement based on what is true for **you**, not what other people think is true or what your camp thinks is true.

| **No.** | | **Questions and Filters** | **Coding Categories** | **Codes** | **Skip to:** | **VARIABLE** |
| --- | --- | --- | --- | --- | --- | --- |
| Q701 | | It is the man who decides what type of sex to have. | STRONGLY AGREE  SOMEWHAT AGREE  SOMEWHAT DISAGREE  STRONGLY DISAGREE | 1  2  3  4 |  | GEM1 |
| Q702 | | A woman’s most important role is to take care of her home and cook for her family. | STRONGLY AGREE  SOMEWHAT AGREE  SOMEWHAT DISAGREE  STRONGLY DISAGREE | 1  2  3  4 |  | GEM2 |
| Q703 | | Men need sex more than women do. | STRONGLY AGREE  SOMEWHAT AGREE  SOMEWHAT DISAGREE  STRONGLY DISAGREE | 1  2  3  4 |  | GEM3 |
| Q704 | You don’t talk about sex, you just do it. | | STRONGLY AGREE  SOMEWHAT AGREE  SOMEWHAT DISAGREE  STRONGLY DISAGREE | 1  2  3  4 |  | GEM4 |
| Q705 | Women who carry condoms on them are ‘cheap’. | | STRONGLY AGREE  SOMEWHAT AGREE  SOMEWHAT DISAGREE  STRONGLY DISAGREE | 1  2  3  4 |  | GEM5 |
| Q706 | A man must have sex with other women, even if things with his wife are fine. | | STRONGLY AGREE  SOMEWHAT AGREE  SOMEWHAT DISAGREE  STRONGLY DISAGREE | 1  2  3  4 |  | GEM6 |
| Q707 | There are times when a woman deserves to be beaten. | | STRONGLY AGREE  SOMEWHAT AGREE  SOMEWHAT DISAGREE  STRONGLY DISAGREE | 1  2  3  4 |  | GEM7 |
| Q708 | It is a woman’s responsibility to avoid getting pregnant. | | STRONGLY AGREE  SOMEWHAT AGREE  SOMEWHAT DISAGREE  STRONGLY DISAGREE | 1  2  3  4 |  | GEM8 |
| Q709 | A man should have the final word about decisions in his home. | | STRONGLY AGREE  SOMEWHAT AGREE  SOMEWHAT DISAGREE  STRONGLY DISAGREE | 1  2  3  4 |  | GEM9 |
| Q710 | Men are always ready to have sex. | | STRONGLY AGREE  SOMEWHAT AGREE  SOMEWHAT DISAGREE  STRONGLY DISAGREE | 1  2  3  4 |  | GEM10 |
| Q711 | A woman should tolerate violence in order to keep her family together. | | STRONGLY AGREE  SOMEWHAT AGREE  SOMEWHAT DISAGREE  STRONGLY DISAGREE | 1  2  3  4 |  | GEM11 |
| Q712 | If a woman cheats on a man, it is okay for him to hit her. | | STRONGLY AGREE  SOMEWHAT AGREE  SOMEWHAT DISAGREE  STRONGLY DISAGREE | 1  2  3  4 |  | GEM12 |
| Q713 | If someone insults a man, he should defend his reputation with force if he has to | | STRONGLY AGREE  SOMEWHAT AGREE  SOMEWHAT DISAGREE  STRONGLY DISAGREE | 1  2  3  4 |  | GEM13 |
| Q714 | A man should be outraged if his wife asks him to use a condom. | | STRONGLY AGREE  SOMEWHAT AGREE  SOMEWHAT DISAGREE  STRONGLY DISAGREE | 1  2  3  4 |  | GEM14 |
| Q715 | It is okay for a man to hit his wife if she won’t have sex with him. | | STRONGLY AGREE  SOMEWHAT AGREE  SOMEWHAT DISAGREE  STRONGLY DISAGREE | 1  2  3  4 |  | GEM15 |

Section 8: Conflict Tactics Scale

Interviewer:

→ The next questions are about things that happen within many relationships, and that your current partner, or any other partner may have done to you. I want you to tell me if your current partner, or any other partner has ever done the following things to you. If you say your partner or any partner has ever done one of the following things, I will then ask you to think about how many times this occurred within the last 12 months and before the last 12 months. By partner, I mean someone you feel or have felt close to or been intimate with. Partner can mean a sexual partner, but does not have to be.

| No. | Questions and Filters | Coding Categories | Codes | Skip to: | VARIABLE |
| --- | --- | --- | --- | --- | --- |
| Q801 | Has your current or any other partner ever insulted you or made you feel bad about yourself? | NO  YES | 0  1 | Q804 | CTSV1 |
| Q802 | In the last 12 months how many times has your current or any other partner insulted you or made you feel bad about yourself? | NEVER  ONCE  2-3 TIMES  4-10 TIMES  >10 TIMES | 1  2  3  4  5 |  | CTSV1_A |
| Q803 | Before the last 12 months how many times has your current or any other partner insulted you or made you feel bad about yourself? | NEVER  ONCE  2-3 TIMES  4-10 TIMES  >10 TIMES | 1  2  3  4  5 |  | CTSV1_B |
| Q804 | Has your current or any other partner ever belittled or humiliated you in front of other people? | NO  YES | 0  1 | Q807 | CTSV2 |
| Q805 | In the last 12 months how many times has your current or any other partner belittled or humiliated you in front of other people? | NEVER  ONCE  2-3 TIMES  4-10 TIMES  >10 TIMES | 1  2  3  4  5 |  | CTSV2_A |
| Q806 | Before the last 12 months how many times has your current or any other partner belittled or humiliated you in front of other people? | NEVER  ONCE  2-3 TIMES  4-10 TIMES  >10 TIMES | 1  2  3  4  5 |  | CTSV2_B |
| Q807 | Has your current or any other partner ever done things to scare or intimidate you on purpose (e.g. by the way he/she looked at you, by yelling and smashing things)? | NO  YES | 0  1 | Q810 | CTSV3 |
| Q808 | In the last 12 months how many times has your current or any other partner ever done things to scare or intimidate you on purpose (e.g. by the way he/she looked at you, by yelling and smashing things)? | NEVER  ONCE  2-3 TIMES  4-10 TIMES  >10 TIMES | 1  2  3  4  5 |  | CTSV3_A |
| Q809 | Before the last 12 months how many times has your current or any other partner ever done things to scare or intimidate you on purpose? | NEVER  ONCE  2-3 TIMES  4-10 TIMES  >10 TIMES | 1  2  3  4  5 |  | CTSV3_B |
| Q810 | Has your current or any other partner ever threatened to hurt you or someone you care about? | NO  YES | 0  1 | Q813 | CTSV4 |
| Q811 | In the last 12 months how many times has your current or any other partner threatened to hurt you or someone you care about? | NEVER  ONCE  2-3 TIMES  4-10 TIMES  >10 TIMES | 1  2  3  4  5 |  | CTSV4_A |
| Q812 | Before the last 12 months how many times has your current or any other partner threatened to hurt you or someone you care about | NEVER  ONCE  2-3 TIMES  4-10 TIMES  >10 TIMES | 1  2  3  4  5 |  | CTSV4_B |
| Q813 | Has your current or any other partner ever slapped or thrown something at you that could hurt you? | NO  YES | 0  1 | Q816 | CTSV5 |
| Q814 | In the last 12 months how many times has your current or any other partner slapped or thrown something at you that could hurt you? | NEVER  ONCE  2-3 TIMES  4-10 TIMES  >10 TIMES | 1  2  3  4  5 |  | CTSV5_A |
| Q815 | Before the last 12 months how many times has your current or any other partner slapped or thrown something at you that could hurt you? | NEVER  ONCE  2-3 TIMES  4-10 TIMES  >10 TIMES | 1  2  3  4  5 |  | CTSV5_B |
| Q816 | Has your current or any other partner ever pushed or shoved you? | NO  YES | 0  1 | Q819 | CTSV6 |
| Q817 | In the last 12 months how many times has your current or any other partner pushed or shoved you? | NEVER  ONCE  2-3 TIMES  4-10 TIMES  >10 TIMES | 1  2  3  4  5 |  | CTSV6_A |
| Q818 | Before the last 12 months how many times has your current or any other partner pushed or shoved you? | NEVER  ONCE  2-3 TIMES  4-10 TIMES  >10 TIMES | 1  2  3  4  5 |  | CTSV6_B |
| Q819 | Has your current or any other partner ever hit you with his fist or something else that could hurt you? | NO  YES | 0  1 | Q822 | CTSV7 |
| Q820 | In the last 12 months how many times has your current or any other partner hit you with his fist or something else that could hurt you? | NEVER  ONCE  2-3 TIMES  4-10 TIMES  >10 TIMES | 1  2  3  4  5 |  | CTSV7_A |
| Q821 | Before the last 12 months how many times has your current or any other partner hit you with his fist or something else that could hurt you? | NEVER  ONCE  2-3 TIMES  4-10 TIMES  >10 TIMES | 1  2  3  4  5 |  | CTSV7_B |
| Q822 | Has your current or any other partner ever kicked you, dragged you or beaten you up? | NO  YES | 0  1 | Q825 | CTSV8 |
| Q823 | In the last 12 months how many times has your current or any other partner kicked you, dragged you or beaten you up? | NEVER  ONCE  2-3 TIMES  4-10 TIMES  >10 TIMES | 1  2  3  4  5 |  | CTSV8_A |
| Q824 | Before the last 12 months how many times has your current or any other partner kicked you, dragged you or beaten you up? | NEVER  ONCE  2-3 TIMES  4-10 TIMES  >10 TIMES | 1  2  3  4  5 |  | CTSV8_B |
| Q825 | Has your current or any other partner ever choked or burnt you on purpose? | NO  YES | 0  1 | Q828 | CTSV9 |
| Q826 | In the last 12 months how many times has your current or any other partner choked or burnt you on purpose? | NEVER  ONCE  2-3 TIMES  4-10 TIMES  >10 TIMES | 1  2  3  4  5 |  | CTSV9_A |
| Q827 | Before the last 12 months how many times has your current or any other partner choked or burnt you on purpose? | NEVER  ONCE  2-3 TIMES  4-10 TIMES  >10 TIMES | 1  2  3  4  5 |  | CTSV9_B |
| Q828 | Has your current or any other partner ever threatened to use or actually used a gun, knife or other weapon that could hurt you? | NO  YES | 0  1 | Q831 | CTSV10 |
| Q829 | In the last 12 months how many times has your current or any other partner threatened to use or actually used a gun, knife or other weapon that could hurt you? | NEVER  ONCE  2-3 TIMES  4-10 TIMES  >10 TIMES | 1  2  3  4  5 |  | CTSV10_A |
| Q830 | Before the last 12 months how many times has your current or any other partner threatened to use or actually used a gun, knife or other weapon that could hurt you? | NEVER  ONCE  2-3 TIMES  4-10 TIMES  >10 TIMES | 1  2  3  4  5 |  | CTSV10_B |
| Q831 | Has your current or any other partner ever physically forced you to have sex when you did not want to? | NO  YES | 0  1 | Q834 | CTSV11 |
| Q832 | In the last 12 months how many times has your current or any other partner physically forced you to have sex when you did not want to? | NEVER  ONCE  2-3 TIMES  4-10 TIMES  >10 TIMES | 1  2  3  4  5 |  | CTSV11_A |
| Q833 | Before the last 12 months how many times has your current or any other partner physically forced you to have sex when you did not want to? | NEVER  ONCE  2-3 TIMES  4-10 TIMES  >10 TIMES | 1  2  3  4  5 |  | CTSV11_B |
| Q834 | Has your current or any other partner ever used threats to make you have sex? | NO  YES | 0  1 | Q837 | CTSV12 |
| Q835 | In the last 12 months how many times has your current or any other partner used threats to make you have sex? | NEVER  ONCE  2-3 TIMES  4-10 TIMES  >10 TIMES | 1  2  3  4  5 |  | CTSV12_A |
| Q836 | Before the last 12 months how many times has your current or any other partner used threats to make you have sex? | NEVER  ONCE  2-3 TIMES  4-10 TIMES  >10 TIMES | 1  2  3  4  5 |  | CTSV12_B |
| Q837 | Has your current or any other partner ever forced you to do something sexual that you found degrading or humiliating? | NO  YES | 0  1 | Q840 | CTSV13 |
| Q838 | In the last 12 months how many times has your current or any other partner forced you to do something sexual that you found degrading or humiliating? | NEVER  ONCE  2-3 TIMES  4-10 TIMES  >10 TIMES | 1  2  3  4  5 |  | CTSV13_A |
| Q839 | Before the last 12 months how many times has your current or any other partner forced you to do something sexual that you found degrading or humiliating? | NEVER  ONCE  2-3 TIMES  4-10 TIMES  >10 TIMES | 1  2  3  4  5 |  | CTSV13_B |
| Interviewer:  → Now I would like you to think about whether or not you have done any of the following to your current partner, or any other partner. If you say you have done one of the following things, I will then ask you to think about how many times this you did these things within the last 12 months and before the last 12 months. Remember, by partner, I mean someone you feel close to or intimate with. Partner can mean a sexual partner, but does not have to be. | | | | | |
| Q840 | Have you ever insulted your current or any other partner and made him/her feel bad about himself/herself? | NO  YES | 0  1 | Q843 | CTSP1 |
| Q841 | In the last 12 months how many times have you insulted your current or any other partner and made him/her feel bad about himself/herself? | NEVER  ONCE  2-3 TIMES  4-10 TIMES  >10 TIMES | 1  2  3  4  5 |  | CTSP1_A |
| Q842 | Before the last 12 months how many times have you insulted your current or any other partner and made him/her feel bad about himself/herself? | NEVER  ONCE  2-3 TIMES  4-10 TIMES  >10 TIMES | 1  2  3  4  5 |  | CTSP1_B |
| Q843 | Have you ever belittled or humiliated your current or any other partner in front of other people? | NO  YES | 0  1 | Q846 | CTSP2 |
| Q844 | In the last 12 months how many times have you belittled or humiliated your current or any other partner in front of other people? | NEVER  ONCE  2-3 TIMES  4-10 TIMES  >10 TIMES | 1  2  3  4  5 |  | CTSP2_A |
| Q845 | Before the last 12 months how many times have you belittled or humiliated your current or any other partner in front of other people? | NEVER  ONCE  2-3 TIMES  4-10 TIMES  >10 TIMES | 1  2  3  4  5 |  | CTSP2_B |
| Q846 | Have you ever done things to scare or intimidate your current or any other partner on purpose (e.g. by the way he/she looked at you, by yelling and smashing things)? | NO  YES | 0  1 | Q849 | CTSP3 |
| Q847 | In the last 12 months how many times have you done things to scare or intimidate your current or any other partner on purpose (e.g. by the way he/she looked at you, by yelling and smashing things)? | NEVER  ONCE  2-3 TIMES  4-10 TIMES  >10 TIMES | 1  2  3  4  5 |  | CTSP3_A |
| Q848 | Before the last 12 months how many times have you done things to scare or intimidate your current or any other partner on purpose (e.g. by the way he/she looked at you, by yelling and smashing things)? | NEVER  ONCE  2-3 TIMES  4-10 TIMES  >10 TIMES | 1  2  3  4  5 |  | CTSP3_B |
| Q849 | Have you ever threatened to hurt your current or any other partner or someone you care about? | NO  YES | 0  1 | Q852 | CTSP4 |
| Q850 | In the last 12 months how many times have you threatened to hurt your current or any other partner or someone you care about? | NEVER  ONCE  2-3 TIMES  4-10 TIMES  >10 TIMES | 1  2  3  4  5 |  | CTSP4_A |
| Q851 | Before the last 12 months how many times have you threatened to hurt your current or any other partner or someone you care about? | NEVER  ONCE  2-3 TIMES  4-10 TIMES  >10 TIMES | 1  2  3  4  5 |  | CTSP4_B |
| Q852 | Have you ever slapped or thrown something at your current or any other partner that could hurt her/him? | NO  YES | 0  1 | Q855 | CTSP5 |
| Q853 | In the last 12 months how many times have you slapped or thrown something at your current or any other partner that could hurt her/him? | NEVER  ONCE  2-3 TIMES  4-10 TIMES  >10 TIMES | 1  2  3  4  5 |  | CTSP5_A |
| Q854 | Before the last 12 months how many times have you slapped or thrown something at your current or any other partner that could hurt her/him? | NEVER  ONCE  2-3 TIMES  4-10 TIMES  >10 TIMES | 1  2  3  4  5 |  | CTSP5_B |
| Q855 | Have you ever pushed or shoved your current or any other partner? | NO  YES | 0  1 | Q858 | CTSP6 |
| Q856 | In the last 12 months how many times have you pushed or shoved your current or any other partner? | NEVER  ONCE  2-3 TIMES  4-10 TIMES  >10 TIMES | 1  2  3  4  5 |  | CTSP6_A |
| Q857 | Before the last 12 months how many times have you pushed or shoved your current or any other partner? | NEVER  ONCE  2-3 TIMES  4-10 TIMES  >10 TIMES | 1  2  3  4  5 |  | CTSP6_B |
| Q858 | Have you ever hit your current or any other partner with your fist or something else that could hurt her/him? | NO  YES | 0  1 | Q861 | CTSP7 |
| Q859 | In the last 12 months how many times have you hit your current or any other partner with your fist or something else that could hurt her/him? | NEVER  ONCE  2-3 TIMES  4-10 TIMES  >10 TIMES | 1  2  3  4  5 |  | CTSP7_A |
| Q860 | Before the last 12 months how many times have you hit your current or any other partner with your fist or something else that could hurt her/him? | NEVER  ONCE  2-3 TIMES  4-10 TIMES  >10 TIMES | 1  2  3  4  5 |  | CTSP7_B |
| Q861 | Have you ever kicked, dragged or beaten up your current or any other partner? | NO  YES | 0  1 | Q864 | CTSP8 |
| Q862 | In the last 12 months how many times have you kicked, dragged or beaten up your current or any other partner? | NEVER  ONCE  2-3 TIMES  4-10 TIMES  >10 TIMES | 1  2  3  4  5 |  | CTSP8_A |
| Q863 | Before the last 12 months how many times have you kicked, dragged or beaten up your current or any other partner? | NEVER  ONCE  2-3 TIMES  4-10 TIMES  >10 TIMES | 1  2  3  4  5 |  | CTSP8_B |
| Q864 | Have you ever choked or burnt your current or any other partner on purpose? | NO  YES | 0  1 | Q867 | CTSP9 |
| Q865 | In the last 12 months how many times have you choked or burnt your current or any other partner on purpose? | NEVER  ONCE  2-3 TIMES  4-10 TIMES  >10 TIMES | 1  2  3  4  5 |  | CTSP9_A |
| Q866 | Before the last 12 months how many times have you choked or burnt your current or any other partner on purpose? | NEVER  ONCE  2-3 TIMES  4-10 TIMES  >10 TIMES | 1  2  3  4  5 |  | CTSP9_B |
| Q867 | Have you ever threatened to use or actually used a gun, knife or other weapon that could hurt your current or any other partner? | NO  YES | 0  1 | Q870 | CTSP10 |
| Q868 | In the last 12 months how many times have you threatened to use or actually used a gun, knife or other weapon that could hurt your current or any other partner? | NEVER  ONCE  2-3 TIMES  4-10 TIMES  >10 TIMES | 1  2  3  4  5 |  | CTSP10_A |
| Q869 | Before the last 12 months how many times have you threatened to use or actually used a gun, knife or other weapon that could hurt your current or any other partner? | NEVER  ONCE  2-3 TIMES  4-10 TIMES  >10 TIMES | 1  2  3  4  5 |  | CTSP10_B |
| Q870 | Have you ever physically forced your current or any other partner to have sex when she/he did not want to? | NO  YES | 0  1 | Q873 | CTSP11 |
| Q871 | In the last 12 months how many times have you physically forced your current or any other partner to have sex when she/he did not want to? | NEVER  ONCE  2-3 TIMES  4-10 TIMES  >10 TIMES | 1  2  3  4  5 |  | CTSP11_A |
| Q872 | Before the last 12 months how many times have you physically forced your current or any other partner to have sex when she/he did not want to? | NEVER  ONCE  2-3 TIMES  4-10 TIMES  >10 TIMES | 1  2  3  4  5 |  | CTSP11_B |
| Q873 | Have you ever used threats to make your current or any other partner have sex? | NO  YES | 0  1 | Q876 | CTSP12 |
| Q874 | In the last 12 months how many times have you used threats to make your current or any other partner have sex? | NEVER  ONCE  2-3 TIMES  4-10 TIMES  >10 TIMES | 1  2  3  4  5 |  | CTSP12_A |
| Q875 | Before the last 12 months how many times have you used threats to make your current or any other partner have sex? | NEVER  ONCE  2-3 TIMES  4-10 TIMES  >10 TIMES | 1  2  3  4  5 |  | CTSP12_B |
| Q876 | Have you ever forced your current or any other partner to do something sexual that she/he found degrading or humiliating? | NO  YES | 0  1 | Q879 | CTSP13 |
| Q877 | In the last 12 months how many times have you forced your current or any other partner to do something sexual that she/he found degrading or humiliating? | NEVER  ONCE  2-3 TIMES  4-10 TIMES  >10 TIMES | 1  2  3  4  5 |  | CTSP13_A |
| Q878 | Before the last 12 months how many times have you forced your current or any other partner to do something sexual that she/he found degrading or humiliating? | NEVER  ONCE  2-3 TIMES  4-10 TIMES  >10 TIMES | 1  2  3  4  5 |  | CTSP13_B |
| SAY: Thank you for sharing that information with me. I’d like to ask a few more questions about your sexual relationships. These questions will focus more on violence you may have experienced in any of your relationships. | | | | | |
| Q879 | When you were growing up (before the age of 12 years) did you undergo any unwanted sexual experiences? By sexual experiences, I mean, inappropriate touching or unwanted sexual intercourse? | NO  YES | 0  1 |  | CHILDVIO1 |
| Q880 | When you were growing up (before the age of 12 years) did you ever experience physical violence? By physical violence, I mean, were you ever hit, hit with an object, punched, kicked or beaten up in a way that resulted in injury, severe pain or other serious harm? | NO  YES | 0  1 |  | CHILDVIO2 |

Section 9: HIV Stigma

***Interviewer:***

→ Now I’m going to ask you a few questions about what members of your camp think about HIV. In response to each question, please let me know if you think no one, very few people, some people, or most people in the camp believe it to be true.

| **No.** | **Questions and Filters** | **Coding Categories** | **Codes** | **Skip to:** | **VARIABLE** |
| --- | --- | --- | --- | --- | --- |
| Q901 | In your camp, how many people think that people with HIV have brought shame on their families? | NO ONE  VERY FEW PEOPLE  SOME PEOPLE  MOST PEOPLE | 1  2  3  4 |  | CMPSTIG1 |
| Q902 | In your camp, how many people would avoid visiting the homes of people with HIV? | NO ONE  VERY FEW PEOPLE  SOME PEOPLE  MOST PEOPLE | 1  2  3  4 |  | CMPSTIG2 |
| Q903 | In your camp, how many people think that if you have HIV you have done wrong behaviors? | NO ONE  VERY FEW PEOPLE  SOME PEOPLE  MOST PEOPLE | 1  2  3  4 |  | CMPSTIG3 |

Section 10: Alcohol and Drug Use

***Interviewer:***

→ Now I’d like to ask you some questions about your drinking habits and substance use. I know that these are sensitive questions and want to remind you that your responses are confidential.

| **No.** | **Questions and Filters** | **Coding Categories** | **Codes** | **Skip to:** | **VARIABLE** |
| --- | --- | --- | --- | --- | --- |
| Q1001 | Have you ever used alcohol **in your lifetime?** | NO  YES | 0  1 | **Q1005** | ALC_EVR |
| Q1002 | **In the past month**, how often did you use any alcohol? If you don’t know exactly, give a best guess. | NEVER  LESS THAN ONCE A WEEK  1-3 TIMES A WEEK  >3 TIMES A WEEK | 0  1  2  3 | **Q1005** | ALC_FRQ |
| Q1003 | **In the past month**, how often, on average did you drink until you were intoxicated? If you don't know exactly give a best guess. | NEVER  LESS THAN ONCE A WEEK  1-3 TIMES A WEEK  >3 TIMES A WEEK | 0  1  2  3 | **Q1005** | DRNK_FRQ |
| Q1004 | **In the past month,** have you had 5 drinks or more during any one given occasion?  Probe: One drink equals one beer, one glass of wine, or one ounce of hard alcohol | NO  YES | 0  1 |  | ALC_BING |
| Q1005 | Have you ever used marijuana in your lifetime? | NO  YES | 0  1 | **Q1007** | MRJ_EVR |
| Q1006 | How often did you smoke marijuana in the last 12 months? | NOT AT ALL  EVERY DAY  A FEW TIMES A WEEK  ONCE OR TWICE A MONTH  ONCE EVERY FEW MONTHS | 0  1  2  3  4 |  | MRJ_FRQ |
| Q1007 | Have you ever smoked a “cocktail” (mixture of heroin and marijuana) in your lifetime? (I want to remind you that your response will be confidential) | NO  YES | 0  1 | **Q1009** | CKT_EVR |
| Q1008 | How often have you smoked “cocktails” in the last 12 months? | NOT AT ALL  EVERY DAY  A FEW TIMES A WEEK  ONCE OR TWICE A MONTH  ONCE EVERY FEW MONTHS | 0  1  2  3  4 |  | CKT_FRQ |
| Q1009 | Have you ever injected heroin, opium, or cocaine? | NO  YES | 0  1 |  | HINJ_EVR |

Section 11: Social Support

***Interviewer:***

→ Now I would like to ask some questions about the support you may be getting from your friends and family.

| **No.** | | **Questions and Filters** | **Coding Categories** | **Codes** | **Skip to:** | **VARIABLE** |
| --- | --- | --- | --- | --- | --- | --- |
| Q1101 | | In the last 12 months, were the following individuals available for you to talk to?  MOTHER  FATHER  SISTERS, AUNTS, OTHER FEMALE RELATIVES  BROTHERS, UNCLES, OTHER MALE RELATIVES  SEXUAL PARTNER  CAMP MEMBERS  OTHER CLOSE FRIENDS OUTSIDE OF THE CAMP  OTHER________________________ | NO  YES | 0  1 | **For each category, if participant indicates “NO”, do not ask follow-up questions (Q1102-Q1104) for that category.** | SSAVLM  SSAVLF  SSAVLFF  SSAVLMF  SSAVLSP  SSAVLCM  SSAVLCF  SSAVLO  SSAVLO_S |
| Q1102 | | In the last 12 months, have you talked about a personal problem with any of the following people?  MOTHER  FATHER  SISTERS, AUNTS, OTHER FEMALE RELATIVES  BROTHERS, UNCLES, OTHER MALE RELATIVES  SEXUAL PARTNER  CAMP MEMBERS  OTHER CLOSE FRIENDS OUTSIDE OF THE CAMP  OTHER________________________ | NO  YES | 0  1 |  | SSTLKM  SSTLKF  SSTLKFF  SSTLKMF  SSTLKSP  SSTLKCM  SSTLKCF  SSTLKO |
| Q1103 | | In the last 12 months, have you talked to any of the following individuals about HIV?  MOTHER  FATHER  SISTERS, AUNTS, OTHER FEMALE RELATIVES  BROTHERS, UNCLES, OTHER MALE RELATIVES  SEXUAL PARTNER  CAMP MEMBERS  OTHER CLOSE FRIENDS OUTSIDE OF THE CAMP  OTHER________________________ | NO  YES | 0  1 |  | SSHIVM  SSHIVF  SSHIVFF  SSHIVMF  SSHIVSP  SSHIVCM  SSHIVCF  SSHIVO |
| Q1104 | In the last 12 months, have you received money or other things you needed from any of the following people?  MOTHER  FATHER  SISTERS, AUNTS, OTHER FEMALE RELATIVES  BROTHERS, UNCLES, OTHER MALE RELATIVES  SEXUAL PARTNER  CAMP MEMBERS  OTHER CLOSE FRIENDS OUTSIDE OF THE CAMP  OTHER________________________ | | NO  YES | 0  1 |  | SSMONM  SSMONF  SSMONFF  SSMONMF  SSMONSP  SSMONCM  SSMONCF  SSMONO |

Section 12: Social Network

­­­­­­­­

***Interviewer:***

→ Now I would like to ask you some questions about your relationships with people at this camp. As you know, your camp provided our research team with a list of the camp members. We used this list to identify camp members, like you, to participate in the study. I would like to ask you to identify the camp members that you know from this list. We will then ask you a few questions about your close friends in this camp. Our team will be asking other camp members to answer similar questions, but we will never reveal anybody’s answers to anybody else. Just as I won’t tell you what any of the other camp members said about you, I will not tell other camp members anything that you say in this interview.

FOR EACH CAMP MEMBER ON THE ROSTER, ASK:

| **No.** | **Questions and Filters** | **Coding Categories** | **Codes** | **Skip to:** | **VARIABLE** |
| --- | --- | --- | --- | --- | --- |
| ***Interviewer:***  → *I would first like you to identify all the people on this list that you know.* | | | | | |
| Q1201 | Do you know this person? | NO  YES | 0  1 | **For person, if participant indicates “NO”, do not ask Q1202 for that person.** | KNOWP |
| ***Interviewer:***  → *Now, for all the camp members you know, I would like you to identify what relationship you have with each person.* | | | | | |
| Q1202 | What relationship do you have with this person? | FRIEND  ACQUAINTANCE  SOMEBODY I DON’T GET ALONG WITH | 1  2  3 |  | RELP |
| ***Interviewer:***  → Now I’d like to ask you some questions about your friends in this camp. You mentioned that these people are your friends: [HAVE COMPUTER LIST THE NAMES OF PEOPLE THEY MENTIONED WERE THEIR FRIENDS] | | | | | |
| Q1203 | Aside from your girlfriend/boyfriend, from the people on this list, who is your closest friend? [PARTICIPANT SELECTS FRIEND 1]  NOTE that the participant should not select his/her boyfriend or girlfriend, as we will ask about that relationship later. | PID |  |  | FRND1 |
| Q1204 | Who is your second closest friend out of the people on this list? [SELECTS FRIEND 2] | PID |  |  | FRND2 |
| Q1205 | Who is your third closest friend out of the people on this list? [SELECTS FRIEND 3] | PID |  |  | FRND3 |
| ***Interviewer:***  → Now you are going to be asked some questions about your two closest friends in your camp. Remember that no information you provide will be shared with anyone outside of our research team. I will start by asking you about FRIEND 1. | | | | | |
| Q1206 | Is FRIEND 1 a man or a woman? | MAN  WOMAN | 1  2 |  | GEND_F1 |
| Q1207 | In addition to friendship, do you have any of the following other relationships with FRIEND 1?  PAST  GIRLFRIEND/BOYFRIEND  HAD SEX, BUT NOT BOYFRIEND/GIRLFRIEND  SISTER/BROTHER  COUSIN  OTHER RELATIVE  CO-WORKER  CO-OWNER OF BUSINESS  OTHER___________________ | NO  YES | 0  1 |  | O_PGBF_F1  O_SEX_F1  O_SIB_F1  O_COS_F1  O_FAM_F1  O_CWK_F1  O_CBUS_F1  O_REL_F1  O_REL_F1_S |
| Q1208 | How long have you known FRIEND 1? | MORE THAN 3 YEARS  1-3 YEARS  6-12 MONTHS  LESS THAN 6 MONTHS, BUT MORE THAN 1 MONTH  1 MONTH OR LESS | 1  2  3  4  5 |  | KNOWF1 |
| Q1209 | How close do you feel towards FRIEND 1? | VERY CLOSE  SOMEWHAT CLOSE  NOT VERY CLOSE | 1  2  3 |  | CLOSEF1 |
| Q1210 | How much do you trust FRIEND 1? | A LOT  SOMEWHAT  NOT VERY MUCH  NOT AT ALL | 1  2  3  4 |  | TRUSTF1 |
| Q1211 | Where do you mostly meet FRIEND 1?  Please select the 1 location where you meet FRIEND 1 the most. | SCHOOL  WORK  CAMP  MOSQUE/CHURCH  STREET  CLUB/BAR  GUEST HOUSE/HOTEL  BEACH/PARTY  HOME  OTHER___________________ | 1  2  3  4  5  6  7  8  9  10 |  | MEETF1  MEETF1_S |
| Q1212 | Do you think FRIEND 1 uses condoms all the time? | NO  YES | 0  1 |  | CNDUSEF1 |
| Q1213 | Do you think FRIEND 1 thinks that he/she should be using condoms all the time? | NO  YES | 0  1 |  | CNDINJF1 |
| Q1214 | Has FRIEND 1 encouraged you to use condoms all the time? | NO  YES | 0  1 |  | CNDADVF1 |
| Q1215 | Do you think FRIEND 1 has more than one sexual partner at the same time? | NO  YES | 0  1 |  | MCPF1 |
| Q1216 | Do you think FRIEND 1 thinks that it’s ok for him/her to have more than one sexual partner at the same time? | NO  YES | 0  1 |  | MCPINJF1 |
| Q1217 | Has FRIEND 1 discouraged you from having more than one sexual partner at the same time? | NO  YES | 0  1 |  | MCPADVF1 |
| Q1218 | Do you think FRIEND 1 hits his sexual partner? | NO  YES | 0  1 | **ASK ONLY IF Q1206 = 1** | IPVF1 |
| Q1219 | Do you think FRIEND 1 thinks there are some situations in which it’s ok to hit his sexual partner? | NO  YES | 0  1 | **ASK ONLY IF Q1206 = 1** | IPVINJF1 |
| Q1220 | Do you think FRIEND 1 gets hit by her sexual partner? | NO  YES | 0  1 | **ASK ONLY IF Q1206 = 2** | VVF1 |
| Q1221 | Do you think FRIEND 1 thinks there are some situations in which it’s ok for her to be hit by her sexual partner? | NO  YES | 0  1 | **ASK ONLY IF Q1206 = 2** | VVINJF1 |
| Q1222 | Has FRIEND 1 discouraged you from hitting your sexual partner? | NO  YES | 0  1 | **ASK ONLY IF Q101 = 1** | IPVADVF1 |
| Q1223 | Has FRIEND 1 encouraged you to request that your sexual partner not hit you? | NO  YES | 0  1 | **ASK ONLY IF Q101 = 2** | VVADVF1 |
| Q1224 | Do you think FRIEND 1 ever had an HIV test? | NO  YES | 0  1 |  | HIVTSTF1 |
| Q1225 | Do you think FRIEND 1 thinks that he/she should have an HIV test? | NO  YES | 0  1 |  | HIVINJF1 |
| Q1226 | Has FRIEND 1 encouraged you to get an HIV test? | NO  YES | 0  1 |  | HIVADVF1 |
| Friend 2  ***Interviewer:***  → I would now like to ask you the same questions, expect now I would like you to think about Friend 2. | | | | | |
| Q1227 | Is FRIEND 2 a man or a woman? | MAN  WOMAN | 1  2 |  | GEND_F2 |
| Q1228 | In addition to friendship, do you have any of the following other relationships with FRIEND 2?  PAST  GIRLFRIEND/BOYFRIEND  HAD SEX, BUT NOT BOYFRIEND/GIRLFRIEND  SISTER/BROTHER  COUSIN  OTHER RELATIVE  CO-WORKER  CO-OWNER OF BUSINESS  OTHER___________________ | NO  YES | 0  1 |  | O_PGBF_F2  O_SEX_F2  O_SIB_F2  O_COS_F2  O_FAM_F1  O_CWK_F2  O_CBUS_F2  O_REL_F2  O_REL_F2_S |
| Q1229 | How long have you known FRIEND 2? | MORE THAN 3 YEARS  1-3 YEARS  6-12 MONTHS  LESS THAN 6 MONTHS, BUT MORE THAN 1 MONTH  1 MONTH OR LESS  DON’T KNOW | 1  2  3  4  5  88 |  | KNOWF2 |
| Q1230 | How close do you feel towards FRIEND 2? | VERY CLOSE  SOMEWHAT CLOSE  NOT VERY CLOSE | 1  2  3 |  | CLOSEF2 |
| Q1231 | How much do you trust FRIEND 2? | A LOT  SOMEWHAT  NOT VERY MUCH  NOT AT ALL | 1  2  3  4 |  | TRUSTF2 |
| Q1232 | Where do you mostly meet FRIEND 2?  Please select the 1 location where you meet FRIEND 2 the most. | SCHOOL  WORK  CAMP  MOSQUE/CHURCH  STREET  CLUB/BAR  GUEST HOUSE/HOTEL  BEACH/PARTY  HOME  OTHER___________________ | 1  2  3  4  5  6  7  8  9  10 |  | MEETF2  MEETF2_S |
| Q1233 | Do you think FRIEND 2 uses condoms all the time? | NO  YES | 0  1 |  | CNDUSEF2 |
| Q1234 | Do you think FRIEND 2 thinks that he/she should be using condoms all the time? | NO  YES | 0  1 |  | CNDINJF2 |
| Q1235 | Has FRIEND 2 encouraged you to use condoms all the time? | NO  YES | 0  1 |  | CNDADVF2 |
| Q1236 | Do you think FRIEND 2 has more than one sexual partner at the same time? | NO  YES | 0  1 |  | MCPF2 |
| Q1237 | Do you think FRIEND 2 thinks that it’s ok for him/her to have more than one sexual partner at the same time? | NO  YES | 0  1 |  | MCPINJF2 |
| Q1238 | Has FRIEND 2 discouraged you from having more than one sexual partner at the same time? | NO  YES | 0  1 |  | MCPADVF2 |
| Q1239 | Do you think FRIEND 2 hits his sexual partner? | NO  YES | 0  1 | **ASK ONLY IF Q1227 = 1** | IPVF2 |
| Q1240 | Do you think FRIEND 2 thinks there are some situations in which it’s ok to hit his sexual partner? | NO  YES | 0  1 | **ASK ONLY IF Q1227 = 1** | IPVINJF2 |
| Q1241 | Do you think FRIEND 1 gets hit by her sexual partner? | NO  YES | 0  1 | **ASK ONLY IF Q1227 = 2** | VVF2 |
| Q1242 | Do you think FRIEND 2 thinks there are some situations in which it’s ok for her to be hit by her sexual partner? | NO  YES | 0  1 | **ASK ONLY IF Q1227 = 2** | VVINJF2 |
| Q1243 | Has FRIEND 2 discouraged you from hitting your sexual partner? | NO  YES | 0  1 | **ASK ONLY IF Q101 = 1** | IPVADVF2 |
| Q1244 | Has FRIEND 2 encouraged you to request that your sexual partner not hit you? | NO  YES | 0  1 | **ASK ONLY IF Q101 = 2** | = |
| Q1245 | Do you think FRIEND 2 ever had an HIV test? | NO  YES | 0  1 |  | HIVTSTF2 |
| Q1246 | Do you think FRIEND 2 thinks that he/she should have an HIV test? | NO  YES | 0  1 |  | HIVINJF2 |
| Q1247 | Has FRIEND 2 encouraged you to get an HIV test? | NO  YES | 0  1 |  | HIVADVF2 |

Section 13: Attitudes towards HIV Risk Behaviors and Intimate Partner Violence

***Interviewer:***

→ I would like to ask your opinion about certain issues related to behaviors towards sex and your sexual partners. Please tell me how strongly you agree with the following statements:

| **No.** | **Questions and Filters** | **Coding Categories** | **Codes** | **Skip to:** | **VARIABLE** |
| --- | --- | --- | --- | --- | --- |
| Q1301 | I should be using condoms all the time | STRONGLY AGREE  SOMEWHAT AGREE  SOMEWHAT DISAGREE  STRONGLY DISAGREE | 1  2  3  4 |  | ATT_CND |
| Q1302 | It’s ok for me to have more than one sexual partner at the same time | STRONGLY AGREE  SOMEWHAT AGREE  SOMEWHAT DISAGREE  STRONGLY DISAGREE | 1  2  3  4 |  | ATT_MCP |
| Q1303 | There are some situations in which it is ok for me to hit my partner | STRONGLY AGREE  SOMEWHAT AGREE  SOMEWHAT DISAGREE  STRONGLY DISAGREE | 1  2  3  4 | **ASK ONLY IF Q101 = 1** | ATT_IPVM |
| Q1304 | There are some situations in which it is ok for me to be hit by my partner. | STRONGLY AGREE  SOMEWHAT AGREE  SOMEWHAT DISAGREE  STRONGLY DISAGREE | 1  2  3  4 | **ASK ONLY IF Q101 = 2** | ATT_IPVF |

Section 14: Sexual Relationships and Risk Behaviors

***Interviewer:***

→ Now I would like to ask some more personal questions about you and your relationships. Please remember that your answers will be confidential.

| **No.** | **Questions and Filters** | **Coding Categories** | **Codes** | **Skip to:** | **VARIABLE** |
| --- | --- | --- | --- | --- | --- |
| Q1401 | Have you ever had sex?  • Vaginal sex is when a man puts his penis in the vagina of a woman; [some people call this [Swahili term].  • Anal sex is when a man puts his penis in the rectum of a man or a woman; [some people call this [Swahili term]. | NO  YES | 0  1 | **Q1409 and then AND SKIP to SECTION 16** | SEX_EVR |
| Q1402 | How old were you when you had sex for the first time? If you can’t recall the exact age, please give a best estimate. | __ __ YEARS  DON’T KNOW | 88 |  | SEX_AGE |
| Q1403 | How would you describe the first time that you had sex? Would you say that you wanted to have sex, you did not want to have sex but it happened anyway, or were you forced to have sex? | WANTED TO HAVE SEX  DID NOT WANT TO HAVE SEX BUT HAPPENED ANYWAY  WAS FORCED TO HAVE SEX | 1  2  3 |  | SEX_1DES |
| Q1404 | Did you experience any of the following types of situations the first time you had sex?  VERBAL THREATS  PHYSICAL THREATS  PHYSICAL FORCE/HOLDING DOWN  BEATING  HUMILIATING OR DEGRADING SEX ACTS | NO  YES | 0  1 |  | SEX1_VER  SEX1_PHY  SEX1_FRC  SEX1_DEG |
| Q1405 | In total, how many people have you ever had sex with in your lifetime? | __ __ # PERSONS |  | *Note that if this value is less than 3, the participant will only be asked about the corresponding # of partners in the Sexual Network Section | SEXE_NUM |
| Q1406 | In total, how many people have you had sex with in the past 12 months?  This includes people you had sex with only once and people you have had sex with regularly, such as a spouse or someone you live with.  If you can’t recall the exact number, please give a best guess. | NONE  __ __ # PERSONS | 0 |  | SXP12_NUM |
| Q1407 | How many of these partners were women and how many were men? | WOMEN___________  MEN___________ |  | **ASK ONLY IF Q100 = 1**  ***This should total Q1406** | MSM_NUM |
| Q1408 | In general, how would you consider your chances of getting HIV/AIDS? Are they small, moderate, great, or no risk at all? | NO RISK AT ALL  SMALL  MODERATE  GREAT | 1  2  3  4 |  | HIVRISK |
| Q1409 | Have you ever had a test for HIV/AIDS? | NO  YES  DON’T KNOW | 0  1  88 | **Q1501 (UNLESS Q1401 =0)** | TESTHIV |
| Q1410 | How many times have you been tested for HIV/AIDS? | __ __ # TIMES TESTED |  |  | TSTHIV_N |
| Q1411 | Have you had a test for HIV/AIDS in the past 12 months? | NO  YES | 0  1 |  | TEST12M |
| Q1412 | Have you ever received your test results? | NO  YES | 0  1 | **Q1501** | RSLTREC |
| Q1413 | What was the result of your most recent HIV test?  Please remember that everything you say in this interview will be kept in confidence. If you are uncomfortable sharing your result, you may choose to skip this question. | POSITIVE  NEGATIVE  INCONCLUSIVE | 1  2  3 |  | HIVSTAT |
| Q1414 | Who have you shared your HIV/AIDS test results with?  Check all that apply  NO ONE  SPOUSE  BOY/GIRLFRIEND  CASUAL PARTNER  FAMILY (Parents, Children, Siblings, etc.)  RELATIVES (Aunts, Uncles, Cousins, Grandparents, etc.)  FRIEND(S)  HEALTH PROFESSIONAL  OTHER ___________________ | NO  YES | 0  1 |  | HIVDISC  HIVDISC_S |

Section 15. Sexual Network

**NOTE: This section should only be asked for participants who have had sex (if Q1401=No, Skip this Section)**

*Interviewer:*

**→** I would now like to ask you about your sexual relationships. When I talk about having sex I mean that you had either vaginal or anal sex with the person.

Many people have different types of sexual partnerships. Some sexual partners are people we have sex with once or twice and some sexual partners are people we have sex with regularly. Some people have many sexual partners and other people have fewer sexual partners. Some people have sex with a partner of the same gender.

In the past 12 months, you mentioned that you have had sex with (Response from Q1406) people. I would like to begin by asking you about the person you have had the longest sexual relationship with during the last 12 months.

| **No.** | **Questions and Filters** | | **Coding Categories** | | **Codes** | **Skip to:** | **VARIABLE** |
| --- | --- | --- | --- | --- | --- | --- | --- |
| PARTNER #1 | | | | | | | |
| Q1501 | | Tell me about the person you have had the longest sexual relationship with. What is the nickname or initials I could use to talk about this person? | PARTNER #1 |  | | ASK ENTIRE SECTION ONLY IF Q1401=1 | NAME_P1 |
| Q1502 | | When was the most recent time you had sex with PARTNER 1? | YEARS AGO__ __  MONTHS AGO__ __  WEEKS AGO__ __  DAYS AGO__ __ |  | |  | RECSEX_P1 |
| Q1503 | | Is PARTNER 1 a man or a woman? | MAN  WOMAN | 1  2 | |  | GEND_P1 |
| Q1504 | | Where did you meet PARTNER 1? | HOME  SCHOOL  WORK  MY CAMP  ANOTHER CAMP  MOSQUE/CHURCH  STREET  CLUB/BAR  GUEST HOUSE  BEACH  PARTY  WEDDING/FUNERAL  OTHER___________________ | 1  2  3  4  5  6  7  8  9  10  11  12  13 | |  | MEET_P1  METP1_S |
| Q1505 | | How long have you been having sex with this partner? | YEARS_________________  MONTHS_______________  WEEKS_________________  DAYS__________________ |  | |  | SEXDY_P1  SEXDM_P1  SEXDW_P1 |
| Q1506 | | What type of relationship do you have with PARTNER 1? | SPOUSE  POLYGAMOUS SPOUSE  COHABITING PARTNER  GIRLFRIEND/BOYFRIEND  FRIEND  CASUAL ACQUAINTANCE  COMMERCIAL SEX WORKER  ONE TIME PARTNER  OTHER___________________ | 1  2  3  4  5  6  7  8  9 | |  | RELP_P1  RELP_P1_S |
| Q1507 | | To the best of your knowledge, how old is _____ PARTNER 1? | __ __ YEARS |  | |  | AGE_P1 |
| Q1508 | | Is PARTNER 1 a member of this camp? | NO  YES  DON’T KNOW | 0  1  88 | |  | CAMPM_P1 |
| Q1509 | | Are you still having sex with PARTNER 1? | NO  YES | 0  1 | |  | SEXCUR_P1 |
| Q1510 | | How often do/did you have sex with PARTNER 1? | EVERY DAY  A FEW TIMES A WEEK  FEW TIMES A MONTH  ABOUT ONCE A MONTH  ONLY 1-2 TIMES EVER | 1  2  3  4  5 | |  | SEXFRQ_P1 |
| Q1511 | | During the last (most recent) time you had sex with this partner, did you use a condom? | NO  YES  DON’T KNOW | 0  1  88 | |  | CNDUSE_P1 |
| Q1512 | | How many times would you say you had sex with PARTNER 1 during the last one month you had sex with him/her? | _____________ |  | |  | NUMSEX_P1 |
| Q1513 | | Of these (Response from Q1512) times, how many times would you say you used a condom? | _____________ |  | |  | NUMCND_P1 |
| Q1514 | | Did you ever give PARTNER 1 money in exchange for having sex with you? | NO  YES | 0  1 | |  | GIVFSP_P1 |
| Q1515 | | Did PARTNER 1 ever give YOU money in exchange for having sex? | NO  YES | 0  1 | |  | RECFSP_P1 |
| Q1516 | | Did you use a condom the last time money was given or exchanged for sex with PARTNER 1? | NO  YES | 0  1 | | **ASK ONLY IF either Q1514 or Q1515 =1** | CNDM_P1 |
| Q1517 | | The last time you had sex with PARTNER 1, did you drink alcohol before or during sex? | NO  YES | 0  1 | |  | DRNK1_P1 |
| Q1518 | | The last time you had sex with PARTNER 1, did PARTNER 1 drink alcohol before or during sex with you? | NO  YES  DON’T KNOW | 0  1  88 | |  | DRINK2_P1 |
| Q1519 | | While you were in a sexual relationship with PARTNER 1, did you have sex with anybody else? | NO  YES | 0  1 | |  | SXCUR_P1 |
| Q1520 | | As far as you know, during the time you were having a sexual relationship with PARTNER 1, do you think PARTNER 1 had sex with other people? | NO  YES | 0  1 | |  | MCP_P1 |
| Q1521 | | How confident are you that you can use a condom with PARTNER 1? | VERY CONFIDENT  SOMEWHAT CONFIDENT  NOT AT ALL CONFIDENT | 1  2  3 | |  | UCND_P1 |
| Q1522 | | How confident are you that you can discuss HIV with PARTNER 1? | VERY CONFIDENT  SOMEWHAT CONFIDENT  NOT AT ALL CONFIDENT | 1  2  3 | |  | TLKHIV_P1 |
| Q1523 | | Have you ever discussed getting an HIV test with PARTNER 1? | NO  YES | 0  1 | |  | TLKTST_P1 |
| Q1524 | | Have you ever gone together with PARTNER 1 for an HIV test? | NO  YES | 0  1 | |  | TOGTST_P1 |
| Q1525 | | Has PARTNER 1 shared the results of their HIV test in the past 12 months? | NO  YES | 0  1 | |  | TSTDISC_P1 |
| Q1526 | | In the past 12 months, has PARTNER 1 ever hit, slapped, kicked, pushed, shoved or otherwise physically hurt you? | NO  YES | 0  1 | |  | VICVIO_P1 |
| Q1527 | | In the past 12 months, have you ever hit, slapped, kicked, pushed, shoved or otherwise physically hurt PARTNER 1? | NO  YES | 0  1 | |  | PERVIO_P1 |
| PARTNER #2  *Interviewer:*  **→** Now lets talk about your other most recent partners (excluding the partner we just talked about). | | | | | | | |
| Q1528 | | Tell me about the person you had sex with most recently within the last 12 months who is not PARTNER 1. What is the nickname or initials I could use to talk about this person? | PARTNER #2 | |  |  | NAME_P2 |
| Q1529 | | When was the most recent time you had sex with PARTNER 2? | MONTHS __ __  WEEKS __ __  DAYS __ __ | |  |  | RECSEX_P2 |
| Q1530 | | Is PARTNER 2 a man or a woman? | MAN  WOMAN | | 1  2 |  | GEND_P2 |
| Q1531 | | Where did you meet PARTNER 2? | HOME  SCHOOL  WORK  MY CAMP  ANOTHER CAMP  MOSQUE/CHURCH  STREET  CLUB/BAR  GUEST HOUSE  BEACH  PARTY  WEDDING/FUNERAL  OTHER___________________ | | 1  2  3  4  5  6  7  8  9  10  11  12  13 |  | MEET_P2  METP1_S |
| Q1532 | | How long have you been having sex with this partner? | YEARS_________________  MONTHS_______________  WEEKS_________________ | |  |  | SEXDY_P2  SEXDM_P2  SEXDW_P2 |
| Q1533 | | What type of relationship do you have with PARTNER 2? | SPOUSE  POLYGAMOUS SPOUSE  COHABITING PARTNER  GIRLFRIEND/BOYFRIEND  FRIEND  CASUAL ACQUAINTANCE  COMMERCIAL SEX WORKER  ONE TIME PARTNER  OTHER___________________ | | 1  2  3  4  5  6  7  8  9 |  | RELP_P2  RELP_P2_S |
| Q1534 | | (If needed: To the best of your knowledge) How old is _____ PARTNER 2)? | __ __ YEARS | |  |  | AGE_P2 |
| Q1535 | | Is PARTNER 2 a member of this camp? | NO  YES  DON’T KNOW | | 0  1  88 |  | CAMPM_P2 |
| Q1536 | | Are you still having sex with PARTNER 2? | NO  YES | | 0  1 |  | SEXCUR_P2 |
| Q1537 | | How often do/did you have sex with PARTNER 2? | EVERY DAY  A FEW TIMES A WEEK  FEW TIMES A MONTH  ABOUT ONCE A MONTH  ONLY 1-2 TIMES EVER | | 1  2  3  4  5 |  | SEXFRQ_P2 |
| Q1538 | | During the last (most recent) time you had sex with this partner, did you use a condom? | NO  YES  DON’T KNOW | | 0  1  88 |  | CNDUSE_P2 |
| Q1539 | | How many times would you say you had sex with PARTNER 2 during the last one month you had sex with him/her? | _____________ | |  |  | NUMSEX_P2 |
| Q1540 | | Of these (Response from Q1539) times, how many times would you say you used a condom? | _____________ | |  |  | NUMCND_P2 |
| Q1541 | | Did you ever give PARTNER 2 money in exchange for having sex with you? | NO  YES | | 0  1 |  | GIVFSP_P2 |
| Q1542 | | Did PARTNER 2 ever give YOU money in exchange for having sex? | NO  YES | | 0  1 |  | RECFSP_P2 |
| Q1543 | | Did you use a condom the last time money was given or exchanged for sex with PARTNER 2? | NO  YES | | 0  1 | **ASK ONLY IF either Q1542 or Q1543 =1** | CNDM_P2 |
| Q1544 | | The last time you had sex with PARTNER 2, did you drink alcohol before or during sex | NO  YES | | 0  1 |  | DRNK1_P2 |
| Q1545 | | The last time you had sex with PARTNER 2, did PARTNER 2 drink alcohol before or during sex with you? | NO  YES  DON’T KNOW | | 0  1  88 |  | DRINK2_P2 |
| Q1546 | | While you were in a sexual relationship with PARTNER 2, did you have sex with anybody else? | NO  YES | | 0  1 |  | SXCUR_P2 |
| Q1547 | | As far as you know, during the time you were having a sexual relationship with PARTNER 2, do you think PARTNER 2 had sex with other people? | NO  YES | | 0  1 |  | MCP_P2 |
| Q1548 | | How confident are you that you can use a condom with PARTNER 2? | VERY CONFIDENT  SOMEWHAT CONFIDENT  NOT AT ALL CONFIDENT | | 1  2  3 |  | UCND_P2 |
| Q1549 | | How confident are you that you can discuss HIV with PARTNER 2? | VERY CONFIDENT  SOMEWHAT CONFIDENT  NOT AT ALL CONFIDENT | | 1  2  3 |  | TLKHIV_P2 |
| Q1550 | | Have you ever discussed getting an HIV test with PARTNER 2? | NO  YES | | 0  1 |  | TLKTST_P2 |
| Q1551 | | Have you ever gone together with PARTNER 2 for an HIV test? | NO  YES | | 0  1 |  | TOGTST_P2 |
| Q1552 | | Has PARTNER 2 shared the results of their HIV test in the past 12 months? | NO  YES | | 0  1 |  | TSTDISC_P2 |
| Q1553 | | In the past 12 months, has PARTNER 2 ever hit, slapped, kicked, pushed, shoved or otherwise physically hurt you? | NO  YES | | 0  1 |  | VICVIO_P2 |
| Q1554 | | In the past 12 months, have you ever hit, slapped, kicked, pushed, shoved or otherwise physically hurt PARTNER 2? | NO  YES | | 0  1 |  | PERVIO_P2 |
| PARTNER #3 | | | | | | | |
| Q1555 | | Tell me about another person you had sex with most recently before PARTNER 2. What is the nickname or initials I could use to talk about this person? | PARTNER #3 | |  |  | NAME_P3 |
| Q1556 | | When was the most recent time you had sex with PARTNER 3? | MONTHS __ __  WEEKS __ __  DAYS __ __ | |  |  | RECSEX_P3 |
| Q1557 | | Is PARTNER 3 a man or a woman? | MAN  WOMAN | | 1  2 |  | GEND_P3 |
| Q1558 | | Where did you meet PARTNER 3? | SCHOOL  WORK  MY CAMP  ANOTHER CAMP  MOSQUE/CHURCH  STREET  CLUB/BAR  GUEST HOUSE  BEACH  PARTY  WEDDING/FUNERAL  OTHER___________________ | | 1  2  3  4  5  6  7  8  9  10  11  12 |  | MEET_P3  METP1_S |
| Q1559 | | How long have you been having sex with this partner? | YEARS_________________  MONTHS_______________  WEEKS_________________ | |  |  | SEXDY_P3  SEXDM_P3  SEXDW_P3 |
| Q1560 | | What type of relationship do you have with PARTNER 3? | SPOUSE  POLYGAMOUS SPOUSE  COHABITING PARTNER  GIRLFRIEND/BOYFRIEND  FRIEND  CASUAL ACQUAINTANCE  COMMERCIAL SEX WORKER  ONE TIME PARTNER  OTHER___________________ | | 1  2  3  4  5  6  7  8  9 |  | RELP_P3  RELP_P3_S |
| Q1561 | | (If needed: To the best of your knowledge) How old is _____ PARTNER 3)? | __ __ YEARS | |  |  | AGE_P3 |
| Q1562 | | Is PARTNER 3 a member of this camp? | NO  YES  DON’T KNOW | | 0  1  88 |  | CAMPM_P3 |
| Q1563 | | Are you still having sex with PARTNER 3? | NO  YES | | 0  1 |  | SEXCUR_P3 |
| Q1564 | | How often do/did you have sex with PARTNER 3? | EVERY DAY  A FEW TIMES A WEEK  FEW TIMES A MONTH  ABOUT ONCE A MONTH  ONLY 1-2 TIMES EVER | | 1  2  3  4  5 |  | SEXFRQ_P3 |
| Q1565 | | During the last (most recent) time you had sex with this partner, did you use a condom? | NO  YES  DON’T KNOW | | 0  1  88 |  | CNDUSE_P3 |
| Q1566 | | How many times would you say you had sex with PARTNER 3 during the last one month you had sex with him/her? | _____________ | |  |  | NUMSEX_P3 |
| Q1567 | | Of these (Response from Q1566) times, how many times would you say you used a condom? | _____________ | |  |  | NUMCND_P3 |
| Q1568 | | Did you ever give PARTNER 3 money in exchange for having sex with you? | NO  YES | | 0  1 |  | GIVFSP_P3 |
| Q1569 | | Did PARTNER 3 ever give YOU money in exchange for having sex? | NO  YES | | 0  1 |  | RECFSP_P3 |
| Q1570 | | Did you use a condom the last time money was given or exchanged for sex with PARTNER 3? | NO  YES | | 0  1 | **ASK ONLY IF either Q1570 or Q1571 =1** | CNDM_P3 |
| Q1571 | | The last time you had sex with PARTNER 3, did you drink alcohol before or during sex | NO  YES | | 0  1 |  | DRNK1_P3 |
| Q1572 | | The last time you had sex with PARTNER 3, did PARTNER 3 drink alcohol before or during sex with you? | NO  YES  DON’T KNOW | | 0  1  88 |  | DRINK2_P3 |
| Q1573 | | While you were in a sexual relationship with PARTNER 3, did you have sex with anybody else? | NO  YES | | 0  1 |  | SXCUR_P3 |
| Q1574 | | As far as you know, during the time you were having a sexual relationship with PARTNER 3, do you think PARTNER 3 had sex with other people? | NO  YES | | 0  1 |  | MCP_P3 |
| Q1575 | | How confident are you that you can use a condom with PARTNER 3? | VERY CONFIDENT  SOMEWHAT CONFIDENT  NOT AT ALL CONFIDENT | | 1  2  3 |  | UCND_P3 |
| Q1576 | | How confident are you that you can discuss HIV with PARTNER 3? | VERY CONFIDENT  SOMEWHAT CONFIDENT  NOT AT ALL CONFIDENT | | 1  2  3 |  | TLKHIV_P3 |
| Q1577 | | Have you ever discussed getting an HIV test with PARTNER 3? | NO  YES | | 0  1 |  | TLKTST_P3 |
| Q1578 | | Have you ever gone together with PARTNER 3 for an HIV test? | NO  YES | | 0  1 |  | TOGTST_P3 |
| Q1579 | | Has PARTNER 3 shared the results of their HIV test in the past 12 months? | NO  YES | | 0  1 |  | TSTDISC_P3 |
| Q1580 | | In the past 12 months, has PARTNER 3 ever hit, slapped, kicked, pushed, shoved or otherwise physically hurt you? | NO  YES | | 0  1 |  | VICVIO_P3 |
| Q1581 | | In the past 12 months, have you ever hit, slapped, kicked, pushed, shoved or otherwise physically hurt PARTNER 3? | NO  YES | | 0  1 |  | PERVIO_P3 |

Section 16: Work and Expenditures

**Interviewer:**

**→** I would now like to ask you about the ways in which you have made money. I will first ask you about wage jobs you may have had (where you worked for someone else for pay) and later I will ask you about any businesses you may own or co-own.

| **No.** | **Questions and Filters** | **Coding Categories** | **Codes** | **Skip to:** | **VARIABLE** |
| --- | --- | --- | --- | --- | --- |
| Q1601 | Did you do any work of any type for pay, profit, or barter during the last 7 days? | NO  YES | 0  1 | **Q1605** | WORK7D |
| Q1602 | Although you did not do any work during the last 7 days, do you have a job to which you will definitely return to work? | NO  YES | 0  1 | **Q1605** | RETWORK |
| Q1603 | Were you available for work during the last 7 days?  By available to work, I mean were you were physically available to work. Reasons for NOT being available to work may include being in school or being sick, disabled, or busy with household duties. | NO  YES | 0  1 |  | AVLBWK |
| Q1604 | When was the last time you did work for pay, profit or gain? | MONTHS AGO_________  YEARS AGO__________  NEVER | 00 | **Q1618**  **Q1618** | LSTWRK_M  LSTWRK_Y |
| *Interviewer:*  **→** For this set of questions, please think about any wage work you have done. By wage work, I mean work you have done for someone else for pay. Keep in mind that you may have been paid in cash or another form or payment. A an activity can be considered wage work even if it was just one time, several times, or something you do daily. Please don't include work you have done for your own business as wage work. | | | | | |
| Q1605 | Did you do any wage work during the **last 12 months**?  Remember that by wage work, I mean work for someone else for pay. You may have been paid in cash or another form or payment. Also, remember that you may have done wage work even if it was just one time, several times or something you do daily. Please don't include work you have done for your own business as wage work. | NO  YES | 0  1 | **Q1612** | WAGE30D |
| Q1606 | What kind of wage work have you done in the 1ast 12 months?  Select all that apply | DALADALA DRIVER OR CONDUDCTOR  FACTORY WORKER  TEACHER  REPAIR PERSON  SALON WORKER  TRANSPORT ITEMS  OTHER___________________ | 1  2  3  4  5  6  7 |  | WAGETP  WAGETP_S |
| Q1607 | Thinking of all the wage work you have done, how much cash did you earn from these activities, on average, over 1 month in the last year?  Probe: Think about any seasonal work you may have done. Try to account for the high and low months and do you best to identify an average monthly income from all your wage jobs. | TSH_______________  WAS NOT PAID | 99 |  | EARN30D |
| Q1608 | Did you receive any payment for this work in any other form? | NO  YES | 0  1 | **Q1610** | PAYOTHF |
| Q1609 | What would you estimate is the value of the other payments in other form that you received, on average, over 1 month in the last year? | TSH_______________ |  |  | OTHFPAY |
| Q1610 | During the last 12 months, how many weeks per month, on average did you work doing these jobs?  This should be between 0.5-4. | WEEKS______________ |  |  | WEEKPM |
| Q1611 | During the last 12 months, how many hours per week did you usually work in these jobs? | HOURS______________ |  |  | HOURPW |
| **Self-Employed**  *Interviewer:*  **→** This next set of questions is about any self-employed activities you have done. By self-employed activity, we mean you own or co-own a business and that you have invested funds in the start-up or maintenance of a business with the expectation of earning a profit. | | | | | |
| Q1612 | Did you operate any business or do any self-employed activity during the **last 12 months**?  Remember, by self-employed activity, we mean you own or co-own a business and that you have invested funds in the start-up or maintenance of a business with the expectation of earning a profit. | NO  YES | 0  1 | **Q1618** | SEMPL |
| Q1613 | What kind of business(es) do you operate?  Select all that apply.  BODA BODA  VIDEO RENTAL  FUNDI  SELL FOOD  SELL CLOTHES OR SHOES  SELL OTHER_____________  OTHER___________________ | NO  YES | 0  1 |  | B_BODA  B_VID  B_FUNDI  B_SELLFD  B_SELLCTHS  B_SELL_O  B_SELL_O_S  B_OTHER  B_OTHER_S |
| Q1614 | For the business you have been operating the longest, how long have you been operating this business? | YEARS_______________  MONTHS________________ |  |  | BUS_DURY  BUS_DURM |
| Q1615 | What were the main sources of start-up capital for your businesses?  Select all that apply.  LOAN FROM FAMILY/FRIENDS  GIFT FROM FAMILY/FRIENDS  SALE OF ASSETS OWNED  PROCEEDS FROM ANOTHER BUSINESS  OWN SAVINGS  LOAN FROM YOSEFO  LOAN FROM OTHER INSTITUTION  LOAN FROM MONEY LENDER  OTHER_____________ | NO  YES | 0  1 |  | BUS_CAP1  BUS_CAP2  BUS_CAP 3  BUS_CAP 4  BUS_CAP 5  BUS_CAP 6  BUS_CAP 7  BUS_CAP 8  BUS_CAP 9  BUS_CAP9-S |
| Q1616 | Thinking of all the businesses you have, what would you estimate to be the total value of your physical capital stock, including all tools, equipment, inputs, supplies, and finished merchandise (goods for sale)?  If your business is co-owned by other individuals, only include the portion of these items that belongs to you. | TSH________________ |  |  | CAPTL_VAL |
| Q1617 | What was your net income (profit) from your businesses on average, over a one month period in the last year? | TSH________________ |  |  | NTINCOME |
| **Credit Borrowed.**  *Interviewer:*  **→** This next set of questions is about Credit. We are interested in knowing about any cash you have borrowed from an institution. We would like you to think about the biggest loan or credit you obtained in the last 12 months for the following set of questions. | | | | | |
| Q1618 | Over the past 12 months, did you borrow cash from an institution? | NO  YES | 0  1 | **Q1631** | CRD_12M |
| Q1619 | Did you have a co-borrower on this loan? | NO  YES | 0  1 |  | CRD_CBOR |
| Q1620 | What was the source of this loan? | BANK OR OTHER INSTITUTION  INSURANCE COMPANY  YOSEFO  MONEY LENDER  EMPLOYER  RELIGIOUS INSTITUTION  OTHER NGO  OTHER__________________ | 1  2  3  4  5  6  7  8 |  | CRDSOURC |
| Q1621 | How much was borrowed? | TSH________________ |  |  | CRDBOR |
| Q1622 | Is the loan/credit re-paid in full? | NO  YES | 0  1 | **Q1624** | CRDPAID |
| Q1623 | Approximately when do you expect to pay back the money in full? | MONTH___________  YEAR___________ |  |  | CRDPAY |
| Q1624 | Total amount to be paid on the loan including interest | TSH________________ |  |  | CRDAMNT |
| Q1625 | What was the interest rate? | ____________ |  |  | INTEREST |
| Q1626 | Did you use the funds from this loan for any of the following business-related purposes?  BUSINESS INPUT  HIRE ADDITIONAL STAFF  BUILD OR REPAIR DWELLING  REPAID BUSINESS LOANS  OTHER_____________ | NO  YES | 0  1 |  | BUSEXP1  BUSEXP2  BUSEXP3  BUSEXP4  BUSEXP5_S |
| Q1627 | You said you have not yet paid this loan back in full. Did you experience any problems that made it difficult for you to pay back the loan? | NO  YES | 0  1 | **ASK ONLY IF Q1622=0** | DIFFPAY |
| Q1628 | What was the main problem you had that made it difficult for you to pay back the loan? | NOT ENOUGH MONEY TO EXPAND OUR BUSINESS  DIDN’T GET ENOUGH PEOPLE TO BUY OUR PRODUCT OR USE OUR SERVICE  SPENT MONEY INSTEAD OF SAVING IT  SOMEONE STOLE OUR PROFITS  OTHER _______________ | 1  2  3  4  5 |  | PAYPROB  PAYPROB_S |
| Q1629 | **Are you responsible for repaying any other loans including personal, family, or business loans in addition to the loan we just discussed?** | NO  YES | 0  1 |  | OTHLOAN |
| Q1630 | What is the total amount that you owe for repayment of all outstanding debts? | TSH________________ |  |  | TOTDEBT |
| **General Finance and Expenditures** | | | | | |
| Q1631 | Did you do any of the following things with your money in the past 30 days?  BOUGHT FOOD  BOUGHT CLOTHING  SPENT IT ON MY FAMILY’S NEEDS  SCHOOL FEES  SAVED THE MONEY  PAID FOR ENTERTAINMENT  BOUGHT ALCOHOL  BOUGHT CIGARETTES  BOUGHT DRUGS  GAVE MONEY FOR SEX  SPENT ON GIRL/BOYFRIEND/  CELL PHONE OR AIRTIME VOUCHER  GAVE SOME MONEY TO THE CAMP  OTHER _______________ (EXPLAIN) | NO  YES | 0  1 |  | PROFEXP1  PROFEXP2  PROFEXP3  PROFEXP4  PROFEXP5  PROFEXP6  PROFEXP7  PROFEXP8  PROFEXP9  PROFEXP10  PROFEXP11  PROFEXP12  PROFEXP13  PROFEXP14 |
| Q1632 | What would you estimate your total savings to be at this time? | TSH________________ |  |  | SAVING |
| Q1633 | How much do other individuals currently owe you? | TSH________________ |  |  | OWEDAMT |

Section 17: Closing

***Interviewer:***

→ *We have come to the end of our discussion. Thank you very much for sharing your thoughts with me. The information you have provided has been very helpful.*
